# Influence of Eat, Sleep, and Console on Infants Pharmacologically Treated for Opioid Withdrawal: A Post Hoc Subgroup Analysis of the ESC-NOW Randomized Clinical Trial

**DOI:** 10.1001/jamapediatrics.2024.0544

**Published:** 2024-04-15

**Authors:** Lori A. Devlin, Zhuopei Hu, Stephanie L. Merhar, Songthip T. Ounpraseuth, Alan E. Simon, Jeannette Y. Lee, Abhik Das, Margaret M. Crawford, Rachel G. Greenberg, P. Brian Smith, Rosemary D. Higgins, Michele C. Walsh, Ward Rice, David A. Paul, Jessie R. Maxwell, Camille M. Fung, Tanner Wright, Julie Ross, Jennifer M. McAllister, Moira Crowley, Sophie K. Shaikh, Lori Christ, Jaime Brown, Julie Riccio, Kara Wong Ramsey, Erica F. Braswell, Lauren Tucker, Karen McAlmon, Krishna Dummula, Julie Weiner, Jessica R. White, Sarah Newman, Jessica N. Snowden, Leslie W. Young

**Affiliations:** 1 Department of Pediatrics, University of Louisville School of Medicine, Louisville, Kentucky; 2 Department of Biostatistics, University of Arkansas for Medical Sciences, Little Rock; 3 University of Cincinnati College of Medicine and Perinatal Institute, Division of Neonatology, Cincinnati Children’s Hospital Medical Center, Cincinnati, Ohio; 4 IDeA States Pediatric Clinical Trials Network (ISPCTN), Environmental Influences on Child Health Outcomes (ECHO) Program, National Institutes of Health, Rockville, Maryland; 5 Social, Statistical and Environmental Sciences Unit, RTI International, Research Triangle Park, North Carolina; 6 Duke Clinical Research Institute, Duke University School of Medicine, Durham, North Carolina; 7 Eunice Kennedy Shriver National Institute of Child Health and Human Development, Bethesda, Maryland; 8 Office of Research and Sponsored Programs, Florida Gulf Coast University, Fort Myers; 9 St Elizabeth Healthcare, Edgewood, Kentucky; 10 Division of Neonatology, Department of Pediatrics, ChristianaCare, Newark, Delaware; 11 University of New Mexico School of Medicine, Albuquerque; 12 Department of Pediatrics, Division of Neonatology, University of Utah School of Medicine, Salt Lake City; 13 Department of Pediatrics, University of South Florida, Tampa; 14 Medical University of South Carolina, Health Shawn Jenkins Children’s Hospital, Charleston; 15 Department of Pediatrics, Rainbow Babies & Children’s Hospital, Case Western Reserve University, Cleveland, Ohio; 16 Department of Pediatrics, Duke University, Durham, North Carolina; 17 Hospital of the University of Pennsylvania, Philadelphia; 18 Department of Pediatrics, Spartanburg Regional Medical Center, Spartanburg, South Carolina; 19 University of Rochester School of Medicine and Dentistry, Rochester, New York; 20 Kapiolani Medical Center for Women & Children, Honolulu, Hawaii; 21 Department of Pediatrics, Nationwide Children’s Hospital, The Ohio State University College of Medicine, Columbus; 22 Department of Pediatrics, University of Mississippi Medical Center, Jackson; 23 Winchester Hospital, Winchester, Massachusetts; 24 Department of Pediatrics, University of Kansas Medical Center, Kansas City, Missouri; 25 Children’s Mercy Hospital, Kansas City, Missouri; 26 Sanford Health, Sioux Falls, South Dakota; 27 University of Nebraska Medical Center, Omaha; 28 Department of Pediatrics, University of Arkansas for Medical Sciences, Little Rock; 29 Larner College of Medicine at the University of Vermont, Burlington; 30 National Center for Health Statistics, Centers for Disease Control and Prevention, Hyattsville, Maryland

## Abstract

**Importance:**

The function-based eat, sleep, console (ESC) care approach substantially reduces the proportion of infants who receive pharmacologic treatment for neonatal opioid withdrawal syndrome (NOWS). This reduction has led to concerns for increased postnatal opioid exposure in infants who receive pharmacologic treatment. However, the effect of the ESC care approach on hospital outcomes for infants pharmacologically treated for NOWS is currently unknown.

**Objective:**

To evaluate differences in opioid exposure and total length of hospital stay (LOS) for pharmacologically treated infants managed with the ESC care approach vs usual care with the Finnegan tool.

**Design, Setting, and Participants:**

This post hoc subgroup analysis involved infants pharmacologically treated in ESC-NOW, a stepped-wedge cluster randomized clinical trial conducted at 26 US hospitals. Hospitals maintained pretrial practices for pharmacologic treatment, including opioid type, scheduled opioid dosing, and use of adjuvant medications. Infants were born at 36 weeks’ gestation or later, had evidence of antenatal opioid exposure, and received opioid treatment for NOWS between September 2020 and March 2022. Data were analyzed from November 2022 to January 2024.

**Exposure:**

Opioid treatment for NOWS and the ESC care approach.

**Main Outcomes and Measures:**

For each outcome (total opioid exposure, peak opioid dose, time from birth to initiation of first opioid dose, length of opioid treatment, and LOS), we used generalized linear mixed models to adjust for the stepped-wedge design and maternal and infant characteristics.

**Results:**

In the ESC-NOW trial, 463 of 1305 infants were pharmacologically treated (143/603 [23.7%] in the ESC care approach group and 320/702 [45.6%] in the usual care group). Mean total opioid exposure was lower in the ESC care approach group with an absolute difference of 4.1 morphine milligram equivalents per kilogram (MME/kg) (95% CI, 1.3-7.0) when compared with usual care (4.8 MME/kg vs 8.9 MME/kg, respectively; *P* = .001). Mean time from birth to initiation of pharmacologic treatment was 22.4 hours (95% CI, 7.1-37.7) longer with the ESC care approach vs usual care (75.4 vs 53.0 hours, respectively; *P* = .002). No significant difference in mean peak opioid dose was observed between groups (ESC care approach, 0.147 MME/kg, vs usual care, 0.126 MME/kg). The mean length of treatment was 6.3 days shorter (95% CI, 3.0-9.6) in the ESC care approach group vs usual care group (11.8 vs 18.1 days, respectively; *P* < .001), and mean LOS was 6.2 days shorter (95% CI, 3.0-9.4) with the ESC care approach than with usual care (16.7 vs 22.9 days, respectively; *P* < .001).

**Conclusion and Relevance:**

When compared with usual care, the ESC care approach was associated with less opioid exposure and shorter LOS for infants pharmacologically treated for NOWS. The ESC care approach was not associated with a higher peak opioid dose, although pharmacologic treatment was typically initiated later.

**Trial Registration:**

ClinicalTrials.gov Identifier: NCT04057820

## Key Points

**Question:**

Does a function-based eat, sleep, console (ESC) care approach modify hospital outcomes for infants pharmacologically treated for neonatal opioid withdrawal?

**Findings:**

In this subgroup analysis from a randomized clinical trial including 463 infants, management with the ESC care approach was associated with a decrease in total opioid exposure, length of opioid treatment, and total length of hospital stay when compared with usual care. Management with the ESC care approach was not associated with a higher peak opioid dose, although pharmacologic treatment was initiated later.

**Meaning:**

The ESC care approach was associated with improved hospital outcomes in a diverse group of infants pharmacologically treated for opioid withdrawal.

## Teaser

This randomized clinical trial examines data for infants pharmacologically treated for neonatal opioid withdrawal in a subgroup analysis to evaluate differences in opioid exposure and length of hospital stay comparing a function-based care approach vs usual care.

## Introduction

As the opioid crisis continues across the United States, opioid use during pregnancy remains a significant public health concern. In 2020, antenatal opioid exposure led to neonatal opioid withdrawal for more than 20 000 US infants.^1^ Neonatal opioid withdrawal syndrome (NOWS) typically manifests with signs of withdrawal in the first few days after birth as placentally transferred opioids are cleared from the infant’s system. Clinical signs of NOWS include irritability, tremors, increased tone, poor sleep, and poor feeding tolerance.^2^ The complex and variable expression of NOWS^3^ supports close medical monitoring during the acute phase of opioid withdrawal.

The Finnegan Neonatal Abstinence Scoring Tool (FNAST)^4^ has been used to clinically assess withdrawal severity in infants with NOWS for more than 50 years. Multiple attempts have been made to simplify and modify this tool, which has been critiqued for its subjectivity, lack of interrater reliability, and tendency to overestimate the need for pharmacologic treatment.^5,6^ The eat, sleep, console (ESC) approach,^7,8^ developed as a contemporary alternative to the FNAST, focuses on the functional components of withdrawal: whether an infant can eat, sleep and be consoled. Generalizable data to support the efficacy and safety of one approach over the other were lacking until recent results from the ESC-NOW trial^9^ demonstrated that the ESC care approach substantially decreased the time until infants were medically ready for discharge and markedly decreased the proportion of infants receiving pharmacologic treatment when compared with usual care with the FNAST.^10^

The ESC care approach, guided by the ESC Care Tool,^8,10,11^ promotes the optimization of nonpharmacologic interventions as first-line treatment and empowers parents and other primary caregivers to participate in the care and medical decision-making for their infants.^7,8,10^ The benefits of early implementation of individualized nonpharmacologic care during the acute phase of withdrawal have been demonstrated.^12,13,14,15,16,17^ However, postnatal opioid treatment continues to be important in achieving physiologic stability for infants with moderate to severe signs of withdrawal that are not well controlled by nonpharmacologic care alone. Historically, early initiation of exogenous opioids, in combination with extended opioid tapers, raised concerns for prolonged postnatal opioid exposure in infants with NOWS.^5^ To address these concerns, quality improvement initiatives over the last decade have focused on optimizing the initiation and duration of postnatal opioid treatment. Results from single-center and regional quality improvement initiatives have shown a reduction in the cumulative dose and duration of opioid therapy in pharmacologically treated infants who are assessed and managed with the ESC approach.^5,7,8,18,19,20,21^ However, these findings have also raised concerns that this approach may lead to a preventable escalation of pharmacologic treatment that would ultimately increase cumulative postnatal opioid exposure and prolong hospitalization.^22^ To date, no large multicenter randomized clinical trials have examined the effect of the ESC care approach on infants pharmacologically treated for NOWS. Therefore, the effect of the ESC care approach on peak and cumulative postnatal opioid dosing in a geographically diverse group of infants is unknown.

To address this knowledge gap, a subgroup analysis of the ESC-NOW trial was conducted to evaluate associations between the ESC care approach and hospital outcomes for infants pharmacologically treated for NOWS. We hypothesized that use of the ESC care approach would be associated with a decrease in postnatal opioid exposure and length of hospital stay for these infants when compared with usual care with the FNAST.

## Methods

### Data Sources

Prospectively collected data from the in-hospital portion of the ESC-NOW trial were used for this post hoc subgroup analysis. The ESC-NOW trial is a multicenter stepped-wedge, cluster randomized trial conducted at 26 US sites participating in the Advancing Clinical Trials in Neonatal Opioid Withdrawal (ACT NOW) Collaborative.^23^ The University of Arkansas Medical Center served as the central institutional review board for the trial with reliance agreements for all sites. The in-hospital portion of the study discussed here was conducted under waiver of informed consent as approved by the institutional review board. Consolidated Standards of Reporting Trials (CONSORT) reporting guidelines were followed. Primary trial outcomes and the trial protocol have been previously published (Supplement 1).^9,10^

### Participants

Pharmacologically treated infants enrolled in the ESC-NOW trial were included in this analysis unless they were co-enrolled (n = 10) in the concurrently running ACT NOW Weaning clinical trial (NCT04214834) (Figure); outcomes for these infants were unknown at the time of analysis because of blinding for the length of opioid treatment in the Weaning trial. Infants were born at 36 weeks’ gestation or later and treated for NOWS at 1 of 26 study sites between September 2020 and March 2022.

**Figure.**
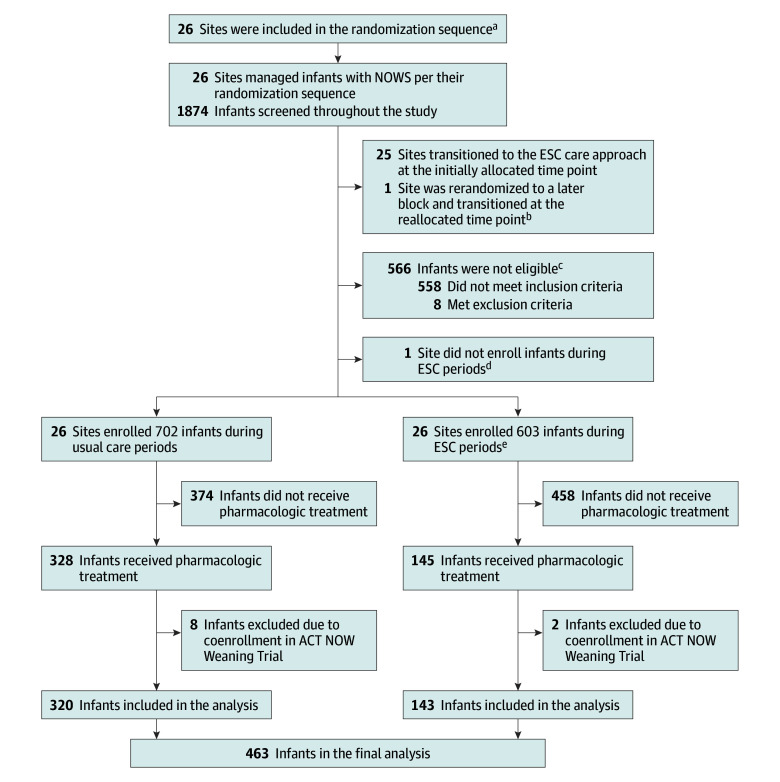
Flow Diagram ACT NOW indicates Advancing Clinical Trials in Neonatal Opioid Withdrawal collaborative; ESC, eat, sleep, console; NOWS, neonatal opioid withdrawal syndrome. ^a^Stratified by proportion of infants who received pharmacologic treatment, lowest third, middle third, and highest third. ^b^Site was in block 2 and unable to transition at the originally allocated time because of site-specific COVID-19–associated limitations or restrictions to training and research activities. ^c^Eligibility criteria are detailed elsewhere.^10^ ^d^Site was in the last block and did not have an eligible infant during the care period after transition. ^e^Three infants who were inadvertently enrolled during the transition period were excluded.

All participating study sites maintained their pretrial practices for pharmacologic treatment, including opioid type (ie, morphine, methadone, or buprenorphine), opioid dosing via opioid taper, and use of adjuvant medications. The lead study investigators reviewed pharmacologic treatment algorithms for each study site before the transition from usual care to the ESC care approach to ensure that the only modifications made were those needed to allow for algorithm use with the ESC Care Tool. This process helped enforce consistency in the approach to treatment at each site throughout the trial. Symptom-based (PRN) dosing was considered a distinct and separate intervention for the ESC-NOW trial, and thus modifications to local treatment algorithms did not include transition to PRN dosing. For infants assessed as needing pharmacologic treatment, a single opioid dose at the time of transfer to a higher level of care in the hospital was permissible, to support a smooth transition between care settings.

### Outcomes

Outcomes for this subgroup analysis included total postnatal opioid exposure (morphine milligram equivalents/kilogram [MME/kg]), total number of opioid doses (count), peak opioid dose (MME/kg), time from birth until initiation of first postnatal opioid dose (hours), length of opioid treatment (days), proportion of infants who received adjuvant therapy (%), and total length of hospital stay (days).

### Statistical Analyses

Data were summarized as mean (SD) for continuous variables and counts with percentages for categorical variables. A 2-sided *P* < .05 significance threshold was used for all analyses without correction for multiple outcomes.

We measured the effect of the ESC care approach on time until opioid treatment was initiated, total number of opioid doses, length of opioid treatment, and total length of hospital stay using generalized linear mixed models (GLMM) with negative binomial distribution. We used GLMM with gamma distribution to examine the effect of the ESC care approach on peak opioid dose and total postnatal opioid exposure (MME/kg) reporting group means, absolute mean difference, incidence rate ratio (IRR), and 95% CI. A conversion factor of 4 for methadone^24^ and 0.03 for buprenorphine^25^ was used to calculate morphine milligram equivalents.

For receipt of adjuvant therapy, we used mixed-effect Poisson regression with robust error variance, reporting adjusted relative risk ratio (RR) with 95% CI. All regression models accounted for the stepped-wedge design with intervention and time as fixed effects and sites as random effects along with the strata indicator (proportion of infants treated pharmacologically at each site before trial initiation according to lowest third, middle third, and highest third). Further adjustment for all baseline maternal and infant characteristics presented in Table 1 was also performed.

**Table 1.** Baseline Characteristics of Mothers and Infants

Characteristic	Group, No./total No. (%)	
	Usual care (n = 320)	ESC care approach (n = 143)
**Maternal**		
Race and ethnicity^a^		
Hispanic	53/313 (17)	10/138 (7)
Non-Hispanic Black	32/313 (10)	10/138 (7)
Non-Hispanic White	218/313 (70)	106/138 (77)
Other^b^	10/313 (3)	12/138 (9)
Adequate prenatal care^c^	163/307 (53)	62/139 (45)
MOUD use	242/311 (78)	101/139 (73)
MOUD type		
Buprenorphine	127/240 (53)	46/100 (46)
Methadone	113/240 (47)	53/100 (53)
Other	0	1/100 (1)
Polysubstance exposure^d^	219 (68)	100 (70)
RUCA^e^		
Metropolitan	257 (80)	129 (90)
Nonmetropolitan	63 (20)	14 (10)
**Infant**		
Sex		
Female	148 (46)	72 (50)
Male	172 (54)	71 (50)
Gestational age, mean (SD), wk	38.6 (1.3)	38.7 (1.4)
Inborn	265 (83)	114 (80)
Postnatal opioid type		
Morphine	240 (75)	101 (71)
Methadone	64 (20)	8 (6)
Buprenorphine	16 (5)	34 (24)

Abbreviations: ESC, eat, sleep, console; MOUD, medication for opioid use disorder; RUCA, rural-urban commuting area.

^a^ Race was obtained from the electronic medical record. Difference in ethnicity between the 2 groups was significant at *P* = .003.

^b^ Other refers to American Indian or Alaska Native, Native Hawaiian or Other Pacific Islander, Asian, and more than 1 race.

^c^ Adequate prenatal care: ≥3 visits and prenatal care before the initiation of the third trimester.

^d^ Polysubstance exposure refers to exposure to opioids in addition to any of the following: amphetamines, barbiturates, benzodiazepines, kratom, cocaine, gabapentin, marijuana, methamphetamines, phencyclidine, and selective serotonin reuptake inhibitors.

^e^ Difference between the 2 groups was significant at *P* = .008.

To assess the robustness of our findings, we conducted a sensitivity analysis to determine if the differences in outcomes noted in the initial analysis were consistent across study sites. Sensitivity analyses were solely intended to identify the presence of treatment heterogeneity by site in the intervention. Data were limited by sample size and distribution of the number of infants in each intervention group, and results were not intended to allow for site-specific inferences to be made.

The sensitivity and other statistical analyses are detailed in eMethods 1 and 2 in Supplement 2. All analyses were conducted using SAS version 9.4 (SAS Institute) and Stata/BE version 18.0 (StataCorp). Data were analyzed from November 2022 to January 2024.

## Results

Of the 1305 infants enrolled in the ESC-NOW trial, 463 infants received pharmacologic treatment as noted in the Figure. Of these infants, 320 (69.1%) were managed with usual care and 143 (30.9%) were managed with the ESC care approach. Demographic characteristics for pharmacologically treated infants and their mothers were similar between the usual care and ESC care approach groups except for differences in ethnicity and rurality (Table 1).

Total adjusted postnatal opioid exposure was less in the ESC care approach group with an absolute mean difference of 4.1 MME/kg (95% CI,1.3-7.0) when compared with usual care (4.8 MME/kg vs 8.9 MME/kg, respectively; *P* = .001) as shown in Table 2. Infants managed with the ESC care approach received a mean of 48.7 fewer (95% CI, 19.9-77.4) opioid doses (67.5 vs 116.1 for usual care; *P* < .001), and they had a 6.3-day decrease (95% CI, 3.0-9.6) in the mean number of opioid treatment days (11.8 vs 18.1 days for usual care; *P* < .001) (Table 2). The adjusted time from birth to initiation of pharmacologic treatment was 22.4 hours longer (95% CI, 7.1-37.7) for infants managed with the ESC care approach (75.4 vs 53.0 hours for usual care; *P* = .002). However, there was no significant difference in adjusted mean peak opioid doses between the ESC care approach and usual care groups (0.126 vs 0.147 MME/kg, respectively) (Table 2).

**Table 2.** Outcome Measures by Intervention Group

Outcome	Unadjusted analysis^a^		Adjusted analysis^b^			
	Usual care	ESC care approach	Usual care	ESC care approach	Absolute effect, difference (95% CI)	Estimated effect (95% CI)
Time until opioid treatment initiated, h^c^	52.6 (48.7 to 56.4)	71.6 (61.6 to 81.6)	53.0 (48.5 to 57.4)	75.4 (62.1 to 88.7)	22.4 (7.1 to 37.7)	1.42 (1.14 to 1.77)^d^
Peak opioid dose, MME/kg^c^	0.161 (0.107 to 0.214)	0.133 (0.085 to 0.180)	0.147 (0.127 to 0.168)	0.126 (0.105 to 0.146)	0.022 (−0.001 to 0.044)	0.85 (0.72 to 1.01)^e^
Total No. of opioid doses^c^	116.0 (73.5 to 158.5)	66.5 (40.6 to 92.4)	116.1 (81.1 to 151.1)	67.5 (45.3 to 89.6)	48.7 (19.9 to 77.4)	0.58 (0.43 to 0.78)^d^
Total postnatal opioid treatment, MME/kg^c^	6.6 (4.5 to 8.6)	3.9 (2.5 to 5.3)	8.9 (5.5 to 12.4)	4.8 (2.8 to 6.7)	4.1 (1.3 to 7.0)	0.54 (0.37 to 0.77)^e^
Length of opioid treatment, d^c^	17.1 (14.3 to 19.9)	11.1 (8.8 to 13.3)	18.1 (14.9 to 21.2)	11.8 (9.3 to 14.3)	6.3 (3.0 to 9.6)	0.65 (0.52 to 0.82)^d^
Length of hospital stay, d^c^	22.4 (19.9 to 24.8)	16.2 (13.9 to 18.4)	22.9 (20.5 to 25.3)	16.7 (14.4 to 19.0)	6.2 (3.0 to 9.4)	0.73 (0.62 to 0.86)^d^
Receipt of adjuvant therapy, %^f^	22.1 (9.3 to 35.0)	15.4 (5.5 to 25.3)	20.0 (6.3 to 33.7)	14.9 (7.3 to 22.6)	5.1 (−8.8 to 19.0)	0.74 (0.35 to 1.56)^g^

Abbreviations: ESC, eat, sleep, console; GLMM, generalized linear mixed model; IRR, incidence rate ratio; MME, morphine milligram equivalents; NOWS, neonatal opioid withdrawal syndrome.

^a^ Analysis was performed without demographic covariates but still accounted for the study design (ie, fixed period/time effect and random site effect) and randomization scheme stratification indicator (proportion of infants with NOWS treated pharmacologically at each site: lowest third, middle third, and highest third).

^b^ Model adjusted for sex, gestational age (weeks), inborn, race, adequate prenatal care, medication for opioid use disorder, polysubstance exposures, pharmacologic treatment medication types, Rural-Urban Commuting Area code, and period/time. Additionally, we adjusted for the randomization scheme stratification indicator (proportion of infants with NOWS treated pharmacologically at each site: lowest third, middle third, and highest third).

^c^ Reported as mean (95% CI).

^d^ Reported as IRR based on a GLMM with negative binomial distribution.

^e^ Reported as IRR based on a GLMM with gamma distribution.

^f^ Reported as estimated probability of use of adjuvant therapy with 95% CI.

^g^ Reported as a relative risk ratio based on mixed-effect Poisson regression model with robust error variance.

Assessment of heterogeneity of treatment effects across individual sites found that the interactions between the treatment and site fixed effect were not statistically significant for the outcomes of total postnatal opioid exposure, total number of opioid doses, peak opioid dose, time from birth to initiation of opioid treatment, and length of opioid treatment (eResults in Supplement 2). The proportion of infants who received a single dose of opioid therapy to support transitions between care settings was 4.2% in the ESC care approach group and 3.7% in the usual care group.

Morphine was the primary opioid used for pharmacologic treatment among infants in the ESC-NOW trial, while methadone and buprenorphine were used in a small proportion of enrolled infants (Table 1). There were no substantial differences in the type of primary opioid used for pharmacologic treatment between the ESC care approach and usual care groups. Opioid type was controlled for in the statistical model.

The mean adjusted total length of hospital stay for infants who received pharmacologic treatment was 6.2 days shorter (95% CI, 3.0-9.4) in the ESC care approach group than in the usual care group (16.7 vs 22.9 days, respectively; *P* < .001) (Table 2). There was no statistically significant difference in the adjusted proportion of infants who received adjuvant therapy between groups (14.9% for ESC care approach vs 20.0% for usual care) (Table 2). Heterogeneity of the treatment effect for total length of hospital stay and receipt of adjuvant therapy was observed across sites and is detailed in eTables 1 and 2 and eFigures 1 and 2 in Supplement 2.

## Discussion

In this subgroup analysis of pharmacologically treated infants who were enrolled in the ESC-NOW trial, we found that infants managed with the ESC care approach tended to receive fewer opioid doses, had a substantial mean reduction in their average cumulative opioid dose, and a marked decrease in their average length of opioid treatment when compared with infants managed with usual care. We also found that pharmacologically treated infants who were assessed with the ESC care approach had a substantially shorter mean total length of hospital stay. Though infants managed with the ESC care approach generally started opioid treatment later, they did not receive higher peak opioid doses or have a protracted duration of pharmacologic treatment. These findings should help allay concerns raised by some that management with the ESC care approach could result in escalated withdrawal severity. Our findings also demonstrate that a focus on the functional components of opioid withdrawal, the ability to eat, sleep, and be consoled, and reliance on nonpharmacologic treatment as first-line therapy for NOWS appear to appropriately assess and manage withdrawal severity and provide a clinically meaningful threshold for the initiation of pharmacologic treatment.

In the previously published primary analysis for the ESC-NOW trial, we found that infants managed with the ESC care approach had a 32–percentage point absolute reduction in the initiation of pharmacologic treatment when compared with those managed with usual care.^10^ This finding suggests that infants who received pharmacologic treatment in the ESC care approach group likely represented those with more substantial opioid withdrawal. Yet we still found a decrease in total postnatal opioid exposure in this population. These findings lend further support to the use of the ESC care approach for all infants with NOWS. Our results align with previous quality improvement initiatives that have also demonstrated a decrease in the duration and dose of postnatal opioid treatment when infants are managed with the ESC approach.^5,7,8,18,19,21^ However, unlike quality improvement initiatives during which implementation of the ESC care approach occurred in conjunction with other interventions, such as the introduction of PRN dosing and changes in opioid medications over time,^26^ our study directly assessed the associations between the ESC care approach and postnatal opioid exposure without the introduction of other practice changes.

ESC-NOW trial sites maintained the use of a site-specific opioid taper as a part of their pharmacologic treatment algorithms throughout the duration of the study; modifications to algorithms were limited to those needed for the transition to the ESC care approach.^9,10^ Each site maintained their preferred opioid, unit dosing, and dosing interval throughout the study. A small number of infants received a single opioid dose to facilitate transfer between clinical care settings; this number was comparable between the 2 intervention groups, which limits the influence of the practice on our outcomes. Although PRN dosing has been implemented in parallel with the ESC care approach in many quality improvement initiatives, the ESC-NOW study team deemed this approach to be a discrete and separate intervention from the ESC care approach and one that warranted an independent evaluation. The Optimizing Pharmacologic Treatment for Neonatal Opioid Withdrawal Syndrome (OPTimize NOW), a cluster randomized clinical trial (NCT05980260), has been designed to evaluate the effect of a symptom-based dosing approach on in-hospital and short-term outcomes for infants with NOWS. We anticipate that enrollment for this study will begin in early 2024.

The ESC care approach emphasizes and provides structure for the consistent use of nonpharmacologic care as the initial treatment for infants with NOWS. This focus on early nonpharmacologic interventions aligns with the 2020 recommendations from the American Academy of Pediatrics^27^ for the use of an individualized nonpharmacologic care approach as the first step in treatment for infants with NOWS. The American Academy of Pediatrics also acknowledges that pharmacologic treatment may be needed for infants with more severe withdrawal to decrease potential complications associated with NOWS, such as excessive weight loss and impaired state regulation. The judicious use of opioid treatment is key, but strong evidence to support which infants benefit from postnatal pharmacologic treatment and the optimal dosing approach is currently lacking. Some studies suggest that higher doses and longer durations of postnatal opioid exposure may be associated with worse cognitive outcomes^28,29,30,31^ while others suggest that neonatal neurobehavior is improved in pharmacologically treated infants.^32^ Our findings suggest that in a diverse group of infants who were pharmacologically treated for NOWS, the ESC care approach aptly assesses withdrawal severity and supports the management of acute opioid withdrawal with a decrease in pharmacologic treatment. However, further research is needed to develop markers that accurately identify infants with NOWS who truly need pharmacologic intervention to attain physiologic stability during the acute phase of withdrawal. Further clarification of the population of infants with NOWS who ultimately benefit from pharmacologic interventions will likely improve both short-term and longer-term neurodevelopmental and behavioral outcomes for infants with antenatal opioid exposure.^33^

Receipt of pharmacologic treatment is a primary driver of the length of stay for infants with NOWS. A recent evaluation of site-to-site variation in the hospital length of stay for infants managed with usual care for NOWS found that the mean length of hospital stay across 30 sites was 19.2 days longer for infants who received pharmacologic treatment than for those who did not.^34^ In this analysis, we found use of the ESC care approach was associated with a 6.2-day decrease in the mean length of hospital stay when compared with usual care. Prolonged hospital stays for infants who receive pharmacologic treatment for NOWS often result in separation of the mother/infant dyad, which may negatively impact maternal-infant bonding and attachment^35^ and increase emotional and financial stress for the families. Thus, safely reducing the length of hospital stay for infants who require pharmacologic therapy is critical to enhancing outcomes for infants with NOWS.

The findings from this subgroup analysis suggest that use of the ESC care approach is associated with improved short-term hospital outcomes for infants pharmacologically treated for NOWS. Yet the influence of decreased pharmacologic treatment on neurodevelopmental and behavioral outcomes in the first 2 years of life remains largely unknown. Developmental follow-up in a subpopulation of infants enrolled in the ESC-NOW trial is ongoing and includes measures of family and infant wellness during the first 2 years of age and behavioral and developmental assessments at 2 years of age. Findings from this work will further inform the use of the ESC care approach for infants with NOWS.

### Limitations

This study is limited by the patient populations cared for at each study site, which may not fully represent the clinical population of infants with NOWS across the United States, although this is the largest single prospective study focused on this population to date. Limitations in details of the prospectively collected data are noted elsewhere.^10^ All studies are limited by the quality of the clinical data entered and abstracted from the electronic medical record. A data quality framework that has been previously shown to decrease data abstraction error was used throughout this study.^36,37^

## Conclusions

In this subgroup analysis of data from the ESC-NOW trial, use of the function-based ESC care approach was associated with a decrease in postnatal opioid exposure and a shorter mean total length of stay among infants pharmacologically treated for NOWS. Although pharmacologic treatment was initiated later in the ESC care approach group, there was no significant difference in the peak opioid dose for infants managed with the ESC care approach when compared with those managed with usual care. Planned long-term follow-up will further inform use of the ESC care approach for the management of infants with NOWS.

## Notes

Supplement 1. Trial protocol

Supplement 2. **eMethods 1.** Heterogeneity of intervention effect across individual sites
eResults
**eTable 1. **Length of hospital stay: number of infants in each site by intervention groups **eFigure 1. **Length of hospital stay: unadjusted marginal mean difference of hospital length of stay between usual care and ESC across sites **eTable 2.** Receipt of adjuvant therapy: number of infants in each site by intervention group **eFigure 2.** Receipt of adjuvant therapy: differences in unadjusted average probability of receiving adjuvant therapy between usual care and ESC across sites **eMethods 2.** Statistical analysis outline

Supplement 3. Nonauthor collaborators

Supplement 4. Data sharing statement
