# Supplementary material for: Influence of Eat, Sleep, and Console on Infants Pharmacologically Treated for Opioid Withdrawal: A Post Hoc Subgroup Analysis of the ESC-NOW Randomized Clinical Trial
Source: JAMA Pediatr. 2024 Apr 15;178(6):525–32. doi: 10.1001/jamapediatrics.2024.0544 (PMC11019446; doi:10.1001/jamapediatrics.2024.0544)
Supplement: Supplement 1. — Trial protocol [file jamapediatr-e240544-s001.pdf]

Eating, Sleeping, Consoling for Neonatal Opioid Withdrawal (ESC-NOW): a Function-Based Assessment and Management Approach

UAMS IRB (cIRB) Protocol Number 239729

Version Number: 09

26 September 2022

ClinicalTrials.gov number: NCT04057820

NRN ID number: NICHD-ACT-NOW-0002

Operational Principal Investigator: Jessica Snowden, MD, University of Arkansas for Medical Sciences

Lead Study Investigators: Leslie Young, MD, University of Vermont
Lori Devlin, DO, MHA, University of Louisville
Stephanie Merhar, MD, University of Cincinnati

Subcommittee Members: Brian Smith, MD, MPH, MHS, Duke Clinical Research Institute
Alan Simon, MD, National Institute of Health
Jeannette Lee, PhD, UAMS
Songthip Ounpraseuth, PhD, UAMS
Andrew Bremer, MD, NICHD
Brenda Poindexter, MD, NICHD NRN
Abhik Das, PhD, RTI

Funded by: National Institutes of Health

Summary of Key Changes from Version 01 to Version 02:

Affected Section(s)	Summary of Revisions Made (01 to 02)	Rationale
Overall	Study changed from a 1-year study to a 2-year study. Additional tests and contact times added. Time points for assessments were modified for some assessments.	Long-term follow-up was added, and study was revised to be consisted with 2 parallel Neonatal Opioid Withdrawal Syndrome (NOWS) protocols.
Section 1.5 Study Intervention/ Methods	Updated time points and assessments to be consistent with the details provided later in the protocol.	Long-term follow-up was added, and study was revised to be consisted with 2 parallel Neonatal Opioid Withdrawal Syndrome (NOWS) protocols.
Table 2. Study Objectives and Endpoints Obtained under Waiver of Consent	Revised time points for critical safety outcomes.	Study is now a 2-year study.
Table 3. Study Objectives and Endpoints Obtained under Provision of Consent	Added the following assessments:<br>• Growth, weight, length, head circumference measurements<br>• Bayley Scales of Infant and Toddler Development, 4th Ed.<br>• Adverse Childhood Event (ACE) questionnaire<br>Deleted the following assessment:<br>• Parent's Evaluation of Development Status (PEDS)	Long-term follow-up was added, and study was revised to be consisted with 2 parallel NOWS protocols.
4.1.2.3; Additional Infant-level Exclusion …	Added exclusion for cases in which infant's caregiver does not speak English.	Some of the assessments are not validated in language(s) other than English.
Table 4. Study Event Schedule	Added:<br>• New contact time points when baby is 9, 18, and 24 months of age<br>• Requirement to get periodic updates of participant's contact information<br>• 3-month time point (additions to previous list):<br>o Acute/urgent care and/or ER visits and hospital readmissions<br>o Non-accidental trauma and death<br>• 24-month time point (new time point):<br>New to study:<br>o Adverse Childhood Experiences Questionnaire<br>o Bayley's Exam	Table updated to match changes made to protocol for long term follow-up.

Affected Section(s)	Summary of Revisions Made (01 to 02)	Rationale
	Additional time point for:<br>○ Weight, length, and head circumference<br>○ Non-accidental trauma and death (infant)<br>○ Caregiver questionnaire<br>○ Brief symptom inventory<br>• Clarification that the window for time points is ±3 weeks for time points ≤ 12 months and is ±6 weeks for time points > 12 months.<br><br><u>Deleted</u>:<br>Deleted from study:<br>• Questionnaire deleted (from study):<br>○ Parent's Evaluation of Developmental Status: Developmental Milestones Assessment Level (PEDS:DM-AL)<br>Deleted from specific time point:<br>• 1-month post discharge time-point deletions are:<br>○ Brief Infant Sleep Questionnaire<br>○ Brief Symptom Inventory (Caregivers)<br>○ Maternal Postnatal Attachment Questionnaire<br>○ Parenting Sense of Competence Scale (PSOC) (Caregiver)<br>• 12-months of age time-point deletion:<br>○ Non-accidental trauma and death (infant)	
4.2.2.2; Consent for Assessment of Long-term outcomes (para. 1)	Added that participants will be asked to bring their baby for an in-person follow-up visit at 24 months	New test (Bayley's) added for long-term follow-up
4.2.3; Randomization procedures (para. 2, last line, completing sentence "The protocol study team will …")	Replaced the following text:<br>*"blind participating sites to the their randomly allocated time period until the point at which the site will need to begin planning and training activities in anticipation of site training and implementation of the ESC approach.*<br>With:<br>*notify sites of their allocated block following randomization*	Correction to method.
4.2.6; Post-hospital procedures	Added 24 months of age as a time point for safety outcome(s), for administering several questionnaires, and for conducting the Bayley's exam. Also noted that research personnel are to maintain contact with participants at regular intervals.	Study period increased from 1 year to 2 years. New assessment (Bayley's) added to study.

Affected Section(s)	Summary of Revisions Made (01 to 02)	Rationale
4.2.8; Blinding/Masking	Replaced the following text:<br>*However, it will not be possible to blind responses to questionnaires that are performed with the assistance of research personnel at the participating sites."*<br>With:<br>*For questionnaires completed by phone, each site should develop a site-specific protocol to preserve blinding of those administering the questionnaires*	Method correction.
4.3.2.1; [Secondary Outcomes] Obtained under Waiver of Consent and gathered …	Item 12: [critical safety outcomes]<br>Primary sentence and bullet 1 – added that critical safety outcomes would be evaluated at 24 months (in addition to other time points)<br>Bullet 2: Added: *"These infants may also fail to develop a bond with their primary caregiver(s) during the first months of life, which may further increase the risk for non-accidental trauma and death during the first two years of life."*	Long-term follow-up was added, and study was revised to be consisted with 2 parallel NOWS protocols.
4.3.2.2. [Secondary Outcomes] Obtained for the Subpopulation who provide …	Primary sentence: Changed from "12" to "24" months (for last time point)<br>Item 2, primary bullet 1 and sub-bullets: New text added, specifically, anthropometric growth data will be collected.<br>Item 2, primary bullet 2, sub-bullet 2: deleted "1-month post hospital discharge" as time point for BISQ.<br>Item 2, primary bullets 5 and 6: added 24 month assessment time points	Long-term follow-up was added, and study was revised to be consisted with 2 parallel NOWS protocols.
4.3.2.2 (continued)	Deleted:<br>• A composite measure of critical safety outcomes based on the presence or absence of non-accidental trauma and death will again be assessed at 12 months of age<br>▪ Hypothesis: Infants managed using the ESC care approach will be safe during the first 12 months of age.<br>▪ Infants with undertreated signs of withdrawal may be at increased risk for failing to develop a bond with their primary caregiver(s) in the first months of life, which increases the risk for non-accidental trauma and death during the first two years of age.	Study was changed from a 1-year study to a 2-year study.
4.3.2.2 (continued)	Item 3, bulleted 2<br>Regarding Brief Symptom Inventory time points:<br>o Deleted "1-month post hospital discharge"<br>o Added "24 months [of age]"	Study time points / assessment times changed due to change from 1-yr to 2-yr study and to make study consistent with other NOWS protocols

Affected Section(s)	Summary of Revisions Made (01 to 02)	Rationale
4.3.2.2. (continued)	Items 4 and 5: Deleted time point at 1-month post hospital discharge for MPAQ and PSOC assessments, respectively.	Study time points / assessment times changed due to change from 1-yr to 2-yr study and to make study consistent with other NOWS protocols
4.3.2.2<br><br>(continued)	Added item 7: Assessment using Adverse Childhood Experience (ACE) Questionnaire	New assessment added due to longer follow-up and need to have consistency between NOWS protocols.
4.3.2.3; Obtained for the Subpopulation who Provide …	Added<br><br>Assessment with the Bayley Scales of Infant and Toddler Development, 4th Ed. (New text: all of bullet 2 and associated sub-bullet.)<br><br>Deleted:<br><br>*The protocol study team will assess infant development with the PEDS: Developmental Milestones Assessment Level (PEDS: DM-AL) at 12 months of age. The caregiver will complete the questionnaire and it will be sent to a central location for review by the protocol study team.*<br><br>*The PEDS: DM-AL is a validated surveillance and screening tool for children 0-8 years of age, designed for developmental follow-up. The tool enables a detailed look at each developmental domain (e.g. expressive language, receptive language, fine motor, gross motor and social-emotional). Researchers note that the tool has a high internal consistency, test-retest reliability and a high level of concordance between caregiver report and professional or paraprofessional assessment. The tool is also noted to have a sensitivity (85%) and specificity (89%) for infants between 11 and 13 months of age.*[1]	New assessment added due to longer follow-up and need to have consistency between NOWS protocols.
4.4; Potential Risks and Benefits to Participants	Added:<br><br>*Participants recognized to have neurodevelopmental impairment on the Bayley-4 exam will be referred to their primary care providers for follow-up. The study team will communicate and share the report with the caregiver(s) and primary care providers if requested by the participants' caregiver(s) and consent is obtained.*	New assessment added due to longer follow-up and need to have consistency between NOWS protocols.
4.5.2.2.; Retention	Added reimbursement plan for participants' time. Revised method of reimbursement to make it flexible	Addresses UAMS IRB contingency. Flexibility added to method of reimbursement because not all sites have the same payment mechanisms available to them.

Affected Section(s)	Summary of Revisions Made (01 to 02)	Rationale
5.1.4.1; [Analysis of Long-term Outcome Endpoints] Infant Wellness after discharge …	Added 2 entire paragraphs for analysis of child's weight, length, and head circumference.	New analysis information needed because assessment added for 24 months of age.
5.1.4.1; [Analysis of Long-term Outcome Endpoints]	Deleted "1 month post discharge" as a time point for BISQ analysis.	Time points of some assessments changed due to longer follow-up and need to have consistency between NOWS protocols.
5.1.4.2;<br>5.1.4.3;<br>And 5.1.4.5 [Analysis of Long-term Outcome Endpoints] BSI, MPAQ, and PSOC respectively	Corrected time points (deleted 1 month post discharge)	Time points of some assessments changed due to longer follow-up and need to have consistency between NOWS protocols.
5.1.4.7 (Bayley's) and 5.1.4.8 (ACE)	New sections.	New assessments added due to longer follow up and need to have consistency between NOWS protocols.
Deletion from 5.1.4	**<u>Parents' Evaluation of Development Status: Development Milestones Assessment Level (PEDS: DM-AL) at 12-Months of Age</u>**<br><br>The analysis team will calculate descriptive statistics (mean ± SD) for each domain in the PEDS: DM-AL (i.e., expressive language, receptive language, fine motor, gross motor and social-emotional) separately for each treatment group. To compare the PEDS: DM-AL composite scores between two intervention groups, we will perform a linear mixed-effects model with a fixed effect for the intervention group and a random effect for study site. We will report point estimates for the group mean difference along with a 95% CI, and the team will repeat this analytical approach for each of the PEDS: DM-AL domains.	Assessment is no longer going to be done for this protocol.
5.2; Samples size and power estimates	**<u>Added last paragraph.</u>**	Method addition.
Through-out	Corrected minor grammar and typographical errors, including adding abbreviations to list of abbreviations.	Simple corrections

Summary of Key Changes from Version 02 to Version 03:

Affected Section(s)	Summary of Revisions Made (02 to 03)	Rationale
Section 5.2; Sample Size and Power Estimates	In the paragraph immediately following Table 5:<br>• Noted that the 24 sites is the minimum number of sites and that there could be up to 28 sites.<br>• Added "During any single study period (see Table 1), a site may enroll no more than 16 infants."	Procedural update.
New section. Section 6.	Added new section, Section 6, Data Management, to specify the role of RTI International.	This is a cIRB-requested change to provide details on RTI International's role.

Summary of Key Changes from Version 03 to Version 04:

Affected Section(s)	Summary of Revisions Made (03 to 04)	Rationale
Table titles	Updated.	Updated.
Section 1.5, Study Intervention / Methods AND Section 4.2.6 Post-hospital Procedures	Second full paragraph of section 1.5 and 1st full paragraph of section 4.2.6:<br>Clarification of term "medical records," specifically noting that the term includes both primary and linked records	Clarification
Section 4.1.2.3., Additional Infant-level Exclusion Criteria (…) AND Section 4.4, Potential Risks and Benefits to Participants	Revised to indicate that primary caregiver must both speak and read English.	Clarification.
Section 4.2.1., Screening	End of last paragraph, added:<br>"Sites may enroll up to 16 infants per period; screen fails are not included in this total. Only infants enrolled in the study may be approached for consent and included in the long-term follow-up study.	Budgetary restrictions necessitate limiting participants.
Figure 1., Screening and Enrollment Procedures	Added to end of footnote:<br>"To optimize recruitment it will be permissible to obtain initial consent up to one month after discharge.	Protocol change.
Section 4.2.4, Study Intervention and Comparison	Middle of 1st paragraph, added that training can also occur by teleconference	Added acceptable training format.
Section 4.2.4.2, Transition period	Training and Implementation, bullet 5:<br>Changed "20" individuals to "10" individuals and added the ability to use written scenarios (as well as video scenarios)	Protocol change/logistics.

Affected Section(s)	Summary of Revisions Made (03 to 04)	Rationale
Section 4.3.2.1. title	Revised sub-section title to specify that "medical records" includes primary on-site records and linked medical records	Clarification.
Section 4.5.2.1, Recruitment	Second paragraph:<br>• Replaced "video" with "informational brochure" in 2nd to last sentence.<br>• Added last sentence to paragraph, stating that consent may be obtained up to one month after discharge.	Protocol correction & addition.
Section 4.5.2.2., Retention	Middle of 1st paragraph:<br>Included sentence specifying contact information may be obtained from participant's medical record if the participant cannot be reached.	Protocol addition.

Summary of Key Changes from Version 04 to Version 05:

Affected Section(s)	Summary of Revisions Made (04 to 05)	Rationale
List of Abbreviations	Updated to add abbreviations referenced in protocol revisions and deleted BSI (Brief Symptom Inventory)	Consistency with revised assessments.
Tables 3 and 4	Updated for assessments/measures referenced in protocol revision. Specifically, added BITSEA assessment and PROMIS assessment and deleted BSI (Brief Symptom Inventory). Also removed references to Bayley sections on social-emotional and on adaptive behavior	Consistency with revised assessments.
Section 1.4.2.2 and 4.1.2.2	Updated infant level exclusion criteria to include diagnosis of COVID-19, which may interfere with non-pharmacologic care and affect study outcomes.	Perinatal management of potential COVID-19 patients are rapidly changing and may impact study outcomes during the current pandemic.
Section 4.1.2.3	Clarified that participants must be able to write, as well as speak and read, English to participate.	Clarifications based on study procedures requiring more than only the ability to speak and read English.
Section 4.2.2.2	Consent for Assessment of Long-term Outcomes.<br>Added second paragraph to allow for remote consenting.	Changed due to (a) COVID-19 pandemic, and (b) relative availability of participants consenting after discharge.
Section 4.3.2.2	Replaced description of Brief Symptom Inventory (BSI) with description of Patient Reported Outcome Measurement System (PROMIS) Short Forms.	Protocol team chose alternate tool for mental health measures for feasibility concerns and to align with other concurrent neonatal opioid studies.

Affected Section(s)	Summary of Revisions Made (04 to 05)	Rationale
Section 4.3.2.3	Revised protocol to indicate that Bayley-4 scales will be used for Cognitive, Language, and Motor development and BITSEA (Brief Infant-Toddler Social and Emotional Assessment) will be used to assess social-emotional concerns.	Protocol team chose alternate tool for developmental measures for feasibility concerns and to align with other concurrent neonatal opioid studies.
Section 4.4	Revised protocol to indicate triggers for maternal intervention based on PROMIS scores for severe depression.	Consistency with revised measures.
Section 5.1.4.2 and 5.1.4.8	Revised analysis description to reflect analysis of PROMIS and BITSEA as detailed above.	Consistency with revised measures.

Summary of Key Changes from Version 05 to Version 06:

Affected Section(s)	Summary of Revisions Made (05 to 06)	Rationale
Section 4.2.2.1. Waiver of Consent	To last paragraph in section:<br>• Added option to post handout materials.	Additional flexibility for sites.

Summary of Key Changes from Version 06 to Version 07:

Affected Section(s)	Summary of Revisions Made (06 to 07)	Rationale
3.3.2.4. and throughout document when referencing training material location	• Changed reference FROM “MOP” TO “Training and Implementation Manual” for location of materials related to ESC training.	Change in location of materials.
4.2.4.1. Usual Institutional Care Periods	• Added “Periods” to title of section	Clarification.
4.2.4.2. Transition Periods	• Replaced “protocol study” team with “site research” team<br>• Last bullet under “education” subsection: deleted “through pre/post assessments. Post assessments will require 80% correct responses for completion. Participants will be able to retake each of the lessons until he/she achieves a correct response rate of 80%.<br>• Second bullet under “training and implementation” section: deleted “review of instructional manual” as part of the education and training requirement.<br>• Fourth bullet under “training and implementation: subsection:	Protocol clarifications and internal consistency; study procedure changes

Affected Section(s)	Summary of Revisions Made (06 to 07)	Rationale
	○ Changed "co-assess" TO "assess"<br>○ Deleted requirement to co-assess/assess "with gold-star raters"<br>• Second to last bullet under "training and implementation" subsection: Added "training milestones" to the criteria to assess before moving to the ESC intervention period.<br>• Last bullet:<br>○ changed "webinars" TO "coaching calls"<br>○ deleted requirement for calls to be biweekly	
4.2.4.3. (title change)	• Changed section title FROM "ESC Care Approach" TO "ESC Intervention Periods"	Consistency with other study periods
4.2.4.3. ESC Intervention Periods	• Bullet 11:<br>○ Deleted "The protocol study team will provide sites with a protocol. The protocol study team will permit (following review and approval) site level modifications of the protocol to align it with the site's preferred practice.<br>• Second to last bullet:<br>○ Replaced "protocol study" team with "site research" team<br>○ Replaced "study periods" with "ESC intervention periods."<br>○ Deleted numerical (i.e., 10 individuals) requirement for assessing using the ESC IRR tool.<br>○ Changed methods available for the gold-star raters to assess clinical team:<br>▪ Added "a NOWS infant<br>▪ Deleted "scenarios on the training platform"<br>○ Deleted reference to "a centralized training platform" and replaced with reference to "Training and Implementation Manual."<br>• Last bullet:<br>○ Removed multiple references to the use of an electronic platform for training/evaluation.<br>○ Changed the last sentence FROM: The site research team "will enter data into the electronic application and will send it directly to a central repository where the protocol study team will analyze the data and identify fidelity issues that the site research team can address in a timely fashion." TO The site research team "will keep complete ESC Implementation Process Evaluation forms with other study documentation.	Protocol correction and clarification; add flexibility

Affected Section(s)	Summary of Revisions Made (06 to 07)	Rationale
4.2.5, Protocol Adherence	• Third to last bullet: Deleted "as assessed through the education and training platform.<br>• Second to last bullet: Changed FROM "Initial IRR for the clinical team…" TO "Continual IRR fidelity assessment for the clinical team …"	Corrected to match process.
5.4.2, Site Training	• Allow for training of site champions via video conference in addition to other method(s).	Additional method added.
5.5.2. Data Monitoring and Safety	• Added (to 2nd sentence) that the DSMC review will when sites "have completed one full period using the ESC Care Approach."	Clarification

Summary of Key Changes from Version 07 to Version 08:

Affected Section(s)	Summary of Revisions Made (07 to 08)	Rationale
Section 4.2: Schedule of Activities (footnotes)	• Changed window for 24-month time point. Changed from ± 6 weeks to ± 8 weeks.	The ± 8 weeks allows for the Bailey test (part of 24-mo time point) to be done per standard procedure.

Summary of Key Changes from Version 08 to Version 09:

Affected Section(s)	Summary of Revisions Made (08 to 09)	Rationale
4.5.2.2; Retention	• For primary consent (parent/guardian + infant consent) Increased total amount of 24-month time point reimbursement FROM $100 to $150. This is to increase reimbursement for in-person visit from $50 to $100.<br>• Added payments tables for participants who sign infant-only consent and who sign caregiver-only consent	• Increase amount for in person visit from $50 to $100 to provide more equitable payment<br>• Payment information provided in infant-only consent and caregiver-only consent, and provided in CLARA, but not previously included in protocol

Contents

Section 1. Abstract ... 17
1.1. Primary Hypothesis ... 17
1.2. Secondary Hypothesis ... 17
1.3. Study Design Type ... 17
1.4. Eligibility Criteria ... 18
1.4.1. Inclusion Criteria ... 18
1.4.2. Exclusion Criteria ... 18
1.5. Study Intervention/Methods ... 19
1.5.1. Study Objectives and Endpoints ... 20

Section 2. Conflict of Interest Disclosures ... 22
2.1. Financial Conflicts of Interest of the Institutions and Investigators ... 22
2.2. Plan for Managing Identified Financial Conflicts of Interests ... 22

Section 3. Statement of Problem ... 23
3.1. Primary Hypothesis ... 23
3.2. Secondary Hypothesis ... 23
3.3. Background and Rationale ... 23
3.3.1. Public Health Impact ... 23
3.3.2. Background ... 23

Section 4. Methods ... 28
4.1. Study Population ... 28
4.1.1. Inclusion Criteria ... 28
4.1.2. Exclusion Criteria ... 28
4.2. Detailed Study Procedures ... 30
4.2.1. Screening ... 32
4.2.2. Consent Procedures ... 34
4.2.3. Randomization Procedures ... 36
4.2.4. Study Intervention and Comparison ... 38
4.2.5. Protocol Adherence and Compliance Monitoring ... 43

4.2.6. Post-hospital Procedures 43
4.2.7. Data Quality Assurance 44
4.2.8. Blinding/Masking 45
4.3. Study Objectives and Endpoints 45
4.3.1. Primary Outcome 45
4.3.2. Secondary Outcomes 45
4.4. Potential Risks and Benefits to Participants 52
4.5. Recruitment and Retention 54
4.5.1. Site Recruitment and Retention 54
4.5.2. Infant and Parent/Caregiver Recruitment and Retention 54
Section 5. Analytical Plan 58
5.1. Statistical Analysis Plan 58
5.1.1. General Approach 58
5.1.2. Analysis of the Primary Efficacy Endpoints 58
5.1.3. Analysis of the Secondary Endpoints Obtained Under Waiver of Consent 59
5.1.4. Analysis of the Long-term Outcome Endpoints 62
5.2. Sample Size and Power Estimates 65
5.3. Available Population 67
5.4. Projected Recruitment time 68
5.4.1. Site Recruitment 68
5.4.2. Site Training and Implementation 68
5.5. Study Monitoring Plan 68
5.5.1. Adverse Events 68
5.5.2. Data Monitoring and Safety 70
Section 6. DaTA Management 71
Section 7. Publication and Data Sharing Policy 72
Section 8. References 73

List of Tables

Table 1 Stepped-wedge Cluster Randomized Controlled Trial with Transition Period 17
Table 2 Study Objectives and Endpoints: Obtained under waiver of consent 20
Table 3 Study Objectives and Endpoints: Obtained under provision of consent[a] 21
Table 4 Study Event Schedule 30
Table 5 Sample Size Estimates 66
Table 6 Sample Size Estimates for the IBQ-R for the Consented Subpopulation 67
Table 7 Expected Rates of Safety Outcomes 71

List of Figures

Figure 1 Screening and Enrollment Procedures 33
Figure 2 Randomization Flow Diagram 38

List of Abbreviations

ACE	Adverse Childhood Experience
AE	Adverse Event
Bayley-4	Bayley Scales of Infant and Toddler Development, 4th Edition
BISQ	Brief Infant Sleep Questionnaire
BITSEA	Brief Infant-Toddler Social and Emotional Assessment
BMI	Body Mass Index
CAC	Cluster Autocorrelation Coefficient
CI	Confidence Interval
COVID-19	Severe acute respiratory syndrome coronavirus-2
Cox PH	Cox Proportional Hazards
CQ	Caregiver Questionnaire
DCC	Data Coordinating Center
DSMC	Data and Safety Monitoring Committee
EDC	Electronic Data Capture
EHR	Electronic Health Record
ER	Emergency Room
ESC	Eating, Sleeping, Consoling
FCOI	Financial Conflict of Interest
FES	Family Environmental Scale
FNAST	Finnegan Neonatal Abstinence Scoring Tool
GLMM	Generalized Linear Mixed Model
GSI	Global Severity Index
HIPAA	Health Insurance Portability and Accountability Act
IBQ	Infant Behavior Questionnaire
IBQ-R	Infant Behavior Questionnaire – Revised
ICC	Intracluster Correlation Coefficient
IRB	Institutional Review Board
IRR	Inter-Rater Reliability
ISPCTN	IDeA States Pediatric Clinical Trials Network
LOS	Length of Stay

MOP	Manual of Procedures
MPAQ	Maternal Postnatal Attachment Questionnaire
NIH	National Institutes of Health
NRN	Neonatal Research Network
NOWS	Neonatal Opioid Withdrawal Syndrome
PI	Principal Investigator
PROMIS	Patient Reported Outcome Measurement Information System
PSOC	Parenting Sense of Competence
QC	Quality Control
QI	Quality Improvement
SAE	Serious Adverse Event
SD	Standard Deviation
WHO	World Health Organization

SECTION 1. ABSTRACT

1.1. PRIMARY HYPOTHESIS

Among infants evaluated for neonatal opioid withdrawal syndrome (NOWS), the Eating, Sleeping, Consoling (ESC) care approach will reduce the length of time until infants are medically ready for discharge by an average of 4 days, as compared to usual institutional care with the Finnegan Neonatal Abstinence Scoring Tool (FNAST) or modification thereof.

1.2. SECONDARY HYPOTHESIS

Among infants evaluated for NOWS, use of the ESC care approach will result in an improvement in infant neurobehavioral functioning and family well-being, when compared to usual institutional care with the FNAST or modification thereof.

1.3. STUDY DESIGN TYPE

In this stepped-wedge cluster randomized controlled trial with transition period, the protocol study team will compare the ESC care approach to usual institutional care with the FNAST or modification thereof. Randomization will occur at the site level. The protocol study team will randomize approximately 24 sites into 8 blocks. Each block will transition from usual care to the ESC care approach for the evaluation and management of all infants with NOWS at various time intervals (see Table 1). Sites will use the care approach randomly assigned to their block during each study period for the evaluation and management of all infants with NOWS cared for at the site. During the initial birth hospitalization, the site research team will collect data under waiver of consent for infants who meet eligibility criteria (see Section 4.1). The number of infants enrolled per period at each site will vary throughout the study, due to fluctuations in the number of infants managed for NOWS at each site during each period. However, the goal is for each site to enroll at least 4 infants per period. The site research team will obtain informed consent from the legal guardian(s) to obtain long-term outcomes for eligible infants and caregivers. Site research team members may obtain this consent at any point during the hospital stay for infants who meet the trial's inclusion criteria.

Table 1 Stepped-wedge Cluster Randomized Controlled Trial with Transition Period

	Period 1**	Period 2	Period 3	Period 4	Period 5	Period 6	Period 7	Period 8	Period 9	Period 10
**Block 1***	Usual Care	Transition	ESC	ESC	ESC	ESC	ESC	ESC	ESC	ESC
**Block 2**	Usual Care	Usual Care	Transition	ESC	ESC	ESC	ESC	ESC	ESC	ESC
**Block 3**	Usual Care	Usual Care	Usual Care	Transition	ESC	ESC	ESC	ESC	ESC	ESC
**Block 4**	Usual Care	Usual Care	Usual Care	Usual Care	Transition	ESC	ESC	ESC	ESC	ESC
**Block 5**	Usual Care	Usual Care	Usual Care	Usual Care	Usual Care	Transition	ESC	ESC	ESC	ESC

	Period 1**	Period 2	Period 3	Period 4	Period 5	Period 6	Period 7	Period 8	Period 9	Period 10
**Block 6**	Usual Care	Usual Care	Usual Care	Usual Care	Usual Care	Usual Care	Transition	ESC	ESC	ESC
**Block 7**	Usual Care	Usual Care	Usual Care	Usual Care	Usual Care	Usual Care	Usual Care	Transition	ESC	ESC
**Block 8**	Usual Care	Usual Care	Usual Care	Usual Care	Usual Care	Usual Care	Usual Care	Usual Care	Transition	ESC

* Each block will consist of 3 sites
** Each period will be 2 months in duration, except for the transition period, which will be 3 months, and the intervention periods bordering the transition, which will be 1.5 months/6 weeks in duration

1.4. ELIGIBILITY CRITERIA

1.4.1. Inclusion Criteria

1.4.1.1. <u>Site Level</u>

1. The site is willing, able, and has nurse management and administrative commitment to transition to the ESC care approach at the randomly allocated time
2. The site currently uses the FNAST or modification thereof for the assessment of withdrawal severity for infants with NOWS
3. The site currently provides opioid replacement therapy (i.e., morphine, methadone, or buprenorphine) as part of their pharmacologic management of infants with NOWS

1.4.1.2. <u>Infant Level</u>

1. The infant is being managed for NOWS at an eligible site (i.e., receiving non-pharmacologic care, assessments for withdrawal severity, +/- pharmacologic care)
2. The infant is ≥ 36 weeks' gestation
3. The infant satisfies at least 1 of the following criteria:
    a. Maternal history of prenatal opioid use
    b. Maternal toxicology screen positive for opioids during the second and/or third trimester of pregnancy
    c. Infant toxicology screen positive for opioids during the initial hospital stay

1.4.2. Exclusion Criteria

1.4.2.1. <u>Site Level</u>

1. The site currently manages < 20 opioid exposed infants annually
2. The site routinely discharges/transfers infants from the hospital on opioid replacement therapy (i.e., morphine, methadone, or buprenorphine). We define routine discharge/transfer as ≥10% of infants who receive opioid replacement therapy for NOWS at the site

1.4.2.2. Infant Level

1. Infant has major birth defect(s)
2. Infant has neonatal encephalopathy (inclusive of hypoxic ischemic encephalopathy), a metabolic disorder, stroke, intracranial hemorrhage, or meningitis diagnosed by 60 hours of life
3. Infant was receiving respiratory support (any positive pressure or oxygen therapy) unrelated to pharmacologic treatment for NOWS at 60 hours of life
4. Infant was receiving antimicrobial(s) at 60 hours of life
5. Infant has received any major surgical intervention in the first 60 hours of life
6. Postnatal opioid exposure other than for treatment of NOWS in the first 60 hours of life
7. Outborn infants transferred at >60 hours of life or treated with opioids for NOWS at the transferring hospital
8. Infant's biological mother or primary caregiver is positive or under investigation for COVID-19 at 60 hours of life

1.5. STUDY INTERVENTION/METHODS

The protocol study team will randomize sites into study blocks with each block transitioning from usual care to the ESC care approach at a randomly allocated transition period. The protocol study team will compare short- and long-term outcomes for infants managed with the ESC care approach compared to usual institutional care. During the usual care periods, sites will evaluate and manage infants using the usual institutional practices and protocols for the assessment of withdrawal severity and management of infants with NOWS. After a site's transition to the ESC care approach, each site will use the ESC Care Tool for the assessment of withdrawal severity and management of infants with NOWS (see Manual of Procedures [MOP]). If needed, during both the usual care and ESC interventions, site preference will determine the opioid replacement therapy, as well as the adjuvant therapy used. After completion of inpatient monitoring and management for NOWS, the clinical team members will discharge infants based on their usual practice. The site research team will review the medical record and data collection forms to assess the time (days) from birth until the infant was medically ready for discharge. We define medically ready for discharge as the point at which the infant meets ALL of the following criteria:

- ≥ 96 hours life
- Off of opioid replacement therapy (e.g. morphine, methadone, or buprenorphine) for ≥ 48 hours
- Taking 100% of feeds by mouth for ≥ 24 hours
- ≥ 24 hours from initiation of the maximum caloric density infant received during the initial hospital admission
- Receiving no respiratory support for ≥ 24 hours

The site research team will review the primary and any linked medical records of all eligible infants (under waiver of consent) for the composite safety outcome and the critical safety outcome during the first months of life.

Primary caregivers who provide informed consent will complete multiple questionnaires between discharge and 24 months of age and will return at the end of the study for an in-person visit for assessment of infant neurodevelopment and growth (see Table 4, Study Event Schedule). The questionnaires will assess infant wellness, neurobehavioral functioning and development, postnatal attachment and bonding, caregiver well-being, and family environment. If the primary caregiver for the infant changes during the study period, the new caregiver will provide consent and complete the questionnaires, and the site research team will note the change in caregiver.

1.5.1. Study Objectives and Endpoints

Table 2 outlines the primary and secondary objectives and endpoints for the short-term outcomes, obtained under waiver of consent, and Table 3 outlines the secondary objectives and endpoints for the long-term outcomes, obtained under provision of consent.

Table 2 Study Objectives and Endpoints: Obtained under waiver of consent

**Primary Objective**	**Primary Endpoint:**
To determine if the ESC care approach will reduce the time until infants being managed for NOWS are medically ready for discharge.	Time from birth until medically ready for discharge (days).
**Secondary Objectives**	**Secondary Endpoints:**
To determine if the ESC care approach will decrease the proportion of infants who receive opioid replacement therapy (i.e., morphine, methadone, or buprenorphine) for NOWS prior to hospital discharge.	Receipt of opioid replacement therapy (yes/no).
To determine if the ESC care approach will decrease total postnatal opioid exposure as compared to usual care.	Total dose of opioid replacement therapy infant received (total units, units/kg, and morphine equivalents [mg/kg]).
To assess the impact of the ESC care approach on timing of pharmacologic therapy initiation.	Hour of life opioid replacement initiated.
To assess if use of the ESC care approach will decrease the proportion of infants requiring adjuvant therapy (clonidine or phenobarbital) prior to hospital discharge.	Receipt of adjuvant therapy (yes/no).
To assess the impact of the ESC care approach on infant feeding and weight gain.	Maximum percent weight loss during initial birth hospitalization: $\left[\frac{birthweight\ (g) - weight\ nadir (g)}{birthweight\ (g)}\right] x\ 100 = \max percent\ weight\ loss$
To determine if use of the ESC care approach will increase the proportion of infants who receive maternal breastmilk at the time of hospital discharge.	Type of enteral feedings (exclusive maternal breastmilk, combination of formula and maternal breastmilk, exclusive formula feeding) at time of hospital discharge.
To determine if use of the ESC care approach will increase the proportion of mothers who directly breastfeed at the time of discharge.	Direct breastfeeding within 24 hours of hospital discharge (yes/no)
To assess if the ESC care approach will decrease the length of hospital stay.	Time from birth until infants being managed for NOWS are discharged from the hospital
To assess safety of ESC care approach.	Inpatient composite safety outcome (present/absent)<br>- Seizures

	- Accidental trauma<br>- Respiratory insufficiency due to opioid therapy<br>Outpatient composite 3-month safety outcome (present/absent)<br>– Acute/urgent care and/or emergency room (ER) visits<br>– Hospital readmissions<br>Critical safety outcome at discharge, 3 and 24 months (present/absent)<br>- Non-accidental trauma and death

Table 3 Study Objectives and Endpoints: Obtained under provision of consent[a]

Secondary Objectives	Secondary Endpoints: (See MOP for each tool/questionnaire)
To determine if the ESC care approach, compared to usual care, will improve infant neurobehavioral functioning.	Infant Behavior Questionnaire (IBQ)*
To assess the impact of the ESC care approach on infant wellness after discharge and until 24 months of age compared to usual care.	- Growth: weight (kg), length (cm), head circumference (cm), and weight for length percentile on the World Health Organization (WHO) growth curves. Anthropometric z-scores and Body Mass Index (BMI)-z at 24 months of age<br>- Sleep: Brief Infant Sleep Questionnaire (BISQ) Questionnaire<br>- Enteral feeds (exclusive maternal breastmilk, combination of maternal breastmilk and formula or exclusive formula) (Caregiver Questionnaire) (CQ)<br>- Any direct breastfeeding (CQ)<br>- Acute/urgent care and/or ER visits (total number of occurrences) (CQ)<br>- Readmissions (number of occurrences) (CQ)<br>- Non-accidental trauma and death (presence or absence)<br>- Adverse Childhood Experiences (ACE) questionnaire
To assess if the ESC care approach, compared to usual care, will improve maternal/caregiver wellbeing.	Patient Reported Outcome Measurement Information System (PROMIS) Short Forms
To assess if the ESC care approach, compared to usual care, improves infant-caregiver attachment and bonding.	Maternal Postnatal Attachment Questionnaire (MPAQ)
To assess if the ESC care approach, compared to usual care will improve maternal/caregiver sense of competency.	Parenting Sense of Competence (PSOC) Scale
To assess if the ESC care approach, compared to usual care, will improve the family environment.	Family Environmental Scale (FES) - Relationship Dimension Form R
To assess if the ESC care approach, compared to usual care will improve infant development.	Bayley Scales of Infant and Toddler Development, Fourth Edition (Bayley 4): Cognitive, Language, and Motor,<br>Brief Infant-Toddler Social and Emotional Assessment (BITSEA)

[a] Assessed at various time points between discharge and 24 months of age (see Study Event Schedule, Table 4)
* Secondary outcome of primary interest for the long-term outcomes portion of the study (see Section 4.3.2.2)

SECTION 2. CONFLICT OF INTEREST DISCLOSURES

2.1. FINANCIAL CONFLICTS OF INTEREST OF THE INSTITUTIONS AND INVESTIGATORS

The study investigators will have no financial conflicts of interest (FCOIs) related to the study outlined in this protocol.

2.2. PLAN FOR MANAGING IDENTIFIED FINANCIAL CONFLICTS OF INTERESTS

Any potential or perceived conflicts of interest, including FCOIs, per Title 42, Code of Federal Regulations, Part 50, Subpart F (50.604 Responsibilities of institutions regarding investigator FCOIs), as amended, requires institutional officials (and all subrecipients) to notify the grants officer of any FCOIs prior to expenditure of any funds and within 60 days of any subsequently identified FCOI. Institutional officials should also notify the National Institute of Child Health and Human Development, ECHO, and the IDeA States Pediatric Clinical Trials Network (ISPCTN) Leadership Committee at the same time regarding the COI management plan following institutional guidelines of each participating center.

SECTION 3. STATEMENT OF PROBLEM

3.1. PRIMARY HYPOTHESIS

Among infants evaluated for NOWS, the ESC care approach will reduce the length of time until infants are medically ready for discharge by an average of 4 days, compared to usual institutional care with the FNAST or modification thereof.

3.2. SECONDARY HYPOTHESIS

Among infants evaluated for NOWS, use of the ESC care approach will result in an improvement in infant neurobehavioral functioning and family well-being, when compared to usual institutional care with the FNAST or modification thereof.

3.3. BACKGROUND AND RATIONALE

3.3.1. Public Health Impact

Increased opioid use has resulted in a dramatic increase in the number of infants born with *in utero* opioid exposure requiring management for NOWS.[2, 3, 4, 5] Despite the significance of this problem, numerous critical gaps remain in our knowledge with respect to the best practices for identification and management of infants with NOWS, as well as our understanding of the outcomes of these infants.[6, 7] The opioid epidemic particularly impacts rural and underserved communities represented by the ISPCTN and participating Neonatal Research Network (NRN) sites, which makes our networks well poised to address these critical gaps and improve the care of infants with NOWS.

3.3.2. Background

3.3.2.1. <u>Scope of the Problem</u>

The medical and non-medical use of opioids has increased substantially in women of childbearing age during the last decade.[8] In the United States, medical professionals wrote and dispensed 259 million opioid prescriptions in 2012 alone, an average of 82.5 opioid prescriptions for every 100 persons.[9] Approximately 28% of privately insured and 39% of Medicaid-enrolled women between 15 and 44 years of age filled an opioid prescription annually between 2008 and 2012.[10] Every 3 minutes, a woman seeks care in an emergency department for prescription opioid misuse. In addition, illicit opioid abuse is also increasing dramatically.[8] Nearly 600,000 Americans reported a substance-use disorder involving heroin in 2015, with the strongest risk factor for heroin use being a history of prescription opioid misuse.[11, 4] The national rate of opioid use disorders in new mothers has quadrupled between 1999 and 2014, increasing from 1.5 to 6.5 per 1000 deliveries.[12, 13]

The increased use and misuse of opioids during pregnancy has directly resulted in a 5-fold increase in the incidence of NOWS between 2004 and 2014.[14] A retrospective analysis of a National Inpatient Sample showed that, among infants covered by Medicaid, the incidence of NOWS increased from 2.8 to 14.4 per 1000 births during this same period.[14] Additionally, analysis of an administrative database of 23 hospitals from 2013-2016 demonstrated a continued increase in the incidence of NOWS to 20 per 1000 live births.[15] Significant regional variation in the incidence of NOWS has been noted, with the

highest rates seen in the Northeast and Southeast regions of the United States.[2] Researchers have found an increased incidence of infants with NOWS born to mothers who have high rates of long-term unemployment or who live in mental health shortage areas.[16] Rural areas are disproportionately affected by NOWS, with twice the rate of growth in the number of hospital deliveries complicated by maternal opioid abuse in rural communities compared with the rate of growth in urban communities between 2004-2013.[17] The proportion of infants with NOWS born into rural communities increased from 12.9% in 2003 to 21.2% in 2013.[17] Therefore, improving care for infants with NOWS will particularly impact the rural areas served by many ISPCTN and NRN sites. Additionally, compared with their urban peers, rural infants affected by perinatal opioid misuse are more likely to come from lower-income families who have public insurance.[17] Nationally, state Medicaid programs enroll 60% of mothers with perinatal substance use and more than 80% of infants with NOWS.[2, 3]

3.3.2.2. Recognition and Assessment of Neonatal Opioid Withdrawal Syndrome

Some infants with *in utero* opioid exposure may have mild signs of NOWS that do not significantly impact the infant's ability to feed, sleep, and function, while others may have more severe signs that require pharmacologic therapy to avoid negative effects on growth and development.[18] Physicians use observer-rated scales in clinical practice to quantify the severity of withdrawal and to guide pharmacotherapy.[5] Yet, current scales have not undergone rigorous instrument development and validation.[19, 20] Ninety-five percent of institutions in the United States use the FNAST, with its various modifications.[21] Preliminary data from the ACT NOW Current Experience Study, a chart review conducted at 25 sites within the ISPCTN and 5 sites within the NRN, found that all 30 participating sites used the FNAST or a modification of the FNAST for the assessment of infants with NOWS as part of usual institutional care. Loretta Finnegan developed the FNAST in 1975, and medical personnel currently use this and several modified versions. The tool was initially found to have an inter-rater reliability (IRR) of 0.82 (0.75-0.96), but it has not been subsequently validated for the evaluation of infants with NOWS, although researchers have studied normative values in newborns unexposed to maternal substances.[22] Researchers and clinicians remain concerned about the length of the tool,[23, 24] its inherent subjectivity,[25] and the need to disturb infants for formal assessments.[26] In addition, investigators have concerns that the FNAST and modifications of the FNAST may overestimate the need for pharmacologic therapy, as the formal score incorporates all signs of withdrawal, including those that may not be clinically significant. This overestimation has been linked to increased length of hospital stay and hospital costs.[27]

The ESC Care Tool is an alternative assessment and management tool developed and subsequently implemented at several sites as part of quality improvement (QI) initiatives based on the original ESC approach developed by Grossman and colleagues at Yale.[26] The ESC Care Tool uses a non-invasive, simplified, function-based assessment that evaluates the infant based on his/her ability to eat, sleep, and be consoled. The tool's design provides continued emphases on the role of the family/caregiver in the assessment and care provided for their infants and on non-pharmacologic care as the first-line treatment for infants with NOWS. If an infant is able to feed effectively within 10 minutes of showing hunger (breast-feed well x 10 minutes or take 10 mL [or age-appropriate volume] by alternative feeding method), to sleep undisturbed for 1 hour or longer, and is able to be consoled within 10 minutes, pharmacologic treatment is not initiated or escalated. If the care team assesses that the infant is having difficulties in one of these areas related to NOWS, the care team first attempts to

optimize non-pharmacologic interventions. If these attempts are unsuccessful, the care team will initiate or escalate pharmacologic therapy.

3.3.2.3. Initial Eating, Sleeping, Consoling Approach

The ESC approach, an approach that emphasizes parental involvement, simplifies the assessment of infants with NOWS, and focuses interventions on non-pharmacologic therapies, began its evolution at Yale-New Haven Children's Hospital over a 5-year period of QI work. During this time, the proportion of infants prenatally exposed to methadone who received pharmacologic treatment for NOWS decreased significantly from 98% (54 out of 55 infants) in the baseline period (January 2008-February 2010) to 14% (6 out of 44 infants) in the post-intervention period (May 2015-June 2016), $P < 0.001$. The average length of stay (LOS) for these infants also decreased significantly from 22 to 6 days ($P < 0.001$).[26] There were no reported seizures during the initial birth hospitalization or need for readmission within 30 days of discharge related to signs of withdrawal for the post-intervention group. Although the results of this QI work appear quite impressive, it is unclear how generalizable this work is, as the pre-intervention rate of pharmacologic treatment was much higher than national estimates at 98% of methadone-exposed infants.[5] Additionally many infants with NOWS are exposed to opioids other than methadone (e.g., buprenorphine and illicit opioids).

On direct comparison, Yale-New Haven's ESC approach, studied as a QI measure, appears to trigger the initiation of opioid replacement therapy for significantly fewer infants than use of the FNAST approach. The Yale group, following their transition to ESC-based assessments, completed a retrospective comparison of treatment decisions for 50 consecutive opioid exposed infants (March 2014-Aug 2015).[27] These infants had FNAST scores recorded every 2 to 6 hours, but clinical personnel managed these infants based on their ESC assessments alone. Management decisions based on the ESC assessment resulted in morphine initiation for 6 infants (12%), compared with 31 infants (62%) who medical professionals would have treated using the FNAST ($P < 0.001$). Additionally, using the ESC-based assessments, medical personnel initiated or increased morphine on 8 patient days (3%), compared with 76 patient days (26%) predicted using the FNAST ($P < 0.001$).[27] Again, the study reported no readmissions or adverse events (AEs).

3.3.2.4. Eating, Sleeping, Consoling Care Tool Development

Other groups have subsequently worked to standardize implementation of the assessment and management components of the ESC care approach, through the development and testing of a formal ESC Care Tool. Initial evaluation of the assessment component of the ESC Care Tool, using standardized training and simulated case scenarios, has demonstrated high inter- and intra-rater reliability.[28] Training in the use of the ESC Care Tool and the overall care approach, with standardized training materials, continues to be evaluated and improved, allowing for feasible implementation in even small community hospitals. Faculty at Children's Hospital at Dartmouth-Hitchcock Medical Center, Boston Medical Center, and Yale-New Haven Children's Hospital collaborated to develop training materials, including ESC Care Tool with definitions (see *Training & Implementation Manual*), Newborn Care Diary (see *Training & Implementation Manual*), ESC training video, and written and videotaped case scenarios with answer key. Sites within The Northern New England Perinatal Quality Improvement Network are currently using these materials to facilitate training as part of a network-wide QI initiative.

Physicians at one of the institutions involved in these development efforts, recently published on their QI results following implementation of the ESC care approach. This institution utilized a pilot version of the ESC Care Tool and showed more modest but consistent findings to those at Yale. The researchers found a decrease in pharmacologic treatment from 87% to 40% and a reduction in LOS from 17 to 11 days, with no AEs noted.[29]

3.3.2.5. Further Study

Although outcomes following implementation of the ESC care approach, inclusive of the ESC Care Tool, appear promising and initial accounts suggest that it is safe, we need to rigorously study this care approach to show safety, efficacy, and generalizability of its use in the care of infants with NOWS. Reports on the ESC care approach to date have been from hospitals where the majority of the mothers are compliant with medication-assisted treatment and are highly motivated to care for their infants. Furthermore, the potential effects of the care provided, using the ESC care approach, on infant and family well-being after discharge are unknown and important to assess.[6, 30] In the proposed trial, comparison of the short- and long-term outcomes for infants managed with the ESC care approach versus those managed with usual care will move us closer to an evidence-based approach for the evaluation and management of infants with NOWS, thus meeting a top research priority in the field.[6, 7]

Justification of Hypotheses

- We hypothesize that use of the ESC care approach for the evaluation and management of infants with NOWS will safely reduce the average length of time until infants are medically ready for discharge, compared with usual care with the FNAST or modification thereof. We selected the primary outcome, average length of time until infants are medically ready for discharge, due to the potential for infants to remain in the hospital beyond this point because of social factors and the previously described potential impact of and link between a reduction in hospital stay and the following:
  - Improved maternal and infant attachment/bonding[31, 32]
  - Decreased hospital complications
  - Increased benefit to society in reduced healthcare costs[3, 14, 33]

Additionally, we hypothesize that use of the ESC care approach will have minimal to no impact on infant safety, while resulting in the following outcomes:

- Reduction in the need for initiation of opioid replacement therapy (i.e., morphine, methadone, or buprenorphine)
- Decrease in the total postnatal opioid exposure
- Improvement in the timeliness to initiation of opioid replacement therapy, when required
- Decrease in the need for adjuvant therapy
- Increase in the proportion of infants who directly breastfeed
- Increase in the proportion of infants receiving their mothers’ own breastmilk

We also hypothesize that use of the ESC care approach will improve postnatal attachment and bonding, and will enhance infant well-being and neurobehavioral functioning and development compared to usual care. Further, we hypothesize that use of the ESC care approach will enhance

maternal well-being and the family environment after discharge. An important component of the ESC care approach is the reported fostering of a collaborative relationship between the primary caregiver(s) and the inpatient clinical team through the co-assessment of the infant's severity of withdrawal and shared treatment plan development. Interviews conducted with families as part of the QI implementation of the ESC Care Tool consistently suggest that this element may reduce the social and emotional impact of the infant's hospitalization on the family. However, while many families expressed feeling like they were an integral part of their infants' care team and reported decreased anxiety and reduction in stigma during the initial birth hospitalization,[34] these families were poised to actively participate in the care of their infants, and such results may not be consistent across all families/caregivers. Thus, we must consider that families/caregivers who are not as well poised to actively participate in the care of their infants may experience more stress if their infants are discharged home earlier.

Our assessment of key markers of infant and family well-being in the subpopulation of infants whose caregiver(s) provide informed consent will allow for further insight into safety. This will also provide an opportunity to examine not only often-assessed intermediate outcome variables (time until medically ready for discharge and need for opioid replacement therapy), but also to examine longer-term outcomes, such as infant neurobehavioral functioning and development, maternal-infant attachment and bonding, and family well-being and functioning.

Justification of Study Design

The protocol study team selected a stepped-wedge cluster design due to three main considerations:

1. Transition to the ESC care approach requires a significant cultural shift in the management of infants with NOWS. This type of cultural change is most effective when applied at the level of the population covered by the hospital and not on a subset of random infants with NOWS within the hospital, thus making a cluster study design important.
2. Interim analysis of the ACT NOW Current Experience study allowed for estimation of an intracluster correlation coefficient (ICC) based on the LOS outcome measure. Using LOS as a proxy for our primary outcome, time until infant is medically ready for discharge, the number of sites required to adequately power the trial based on a parallel cluster design with estimated ICC=0.25 would be prohibitive. The stepped wedge design makes the study feasible by allowing each site to serve as its own control in a pre/post analysis and thus, the variation between sites is of less statistical significance.
3. Additionally, the results of QI projects have inspired many healthcare providers to consider transition to the ESC care approach. A brief questionnaire, sent to investigators at the available study sites, demonstrated an increased willingness to participate in the trial if we integrated a transition to the ESC care approach into the study design.

For these reasons, the protocol study team has designed a stepped-wedge cluster randomized trial with the intervention applied at a cluster level and applied to all participating sites by the end of the 20-month study period, with the timing of transition to ESC randomized. This study design also allows for differentiation between the effect of the intervention and unanticipated time-related confounders.

SECTION 4. METHODS

4.1. STUDY POPULATION

4.1.1. Inclusion Criteria

4.1.1.1. Site Level

1. The site is willing, able, and has nurse management and administrative commitment to transition to the ESC care approach at the randomly allocated time
2. The site currently uses the FNAST or modification thereof for the assessment of withdrawal severity for infants with NOWS
3. The site currently provides opioid replacement therapy (i.e., morphine, methadone, or buprenorphine) for the pharmacologic management of infants with NOWS

4.1.1.2. Infant Level

1. The infant is being managed for NOWS at an eligible site (i.e., receiving non-pharmacologic care, assessments for withdrawal severity, +/- pharmacologic care)
2. The infant is ≥ 36 weeks' gestation
3. The infant satisfies at least 1 of the following criteria:
    a. Maternal history of prenatal opioid use
    b. Maternal toxicology screen positive for opioids during the second and/or third trimester of pregnancy
    c. Infant toxicology screen positive for opioids during the initial hospital stay

4.1.2. Exclusion Criteria

4.1.2.1. Site Level

1. The site currently manages < 20 opioid-exposed infants annually
2. The site routinely discharges/transfers infants from the hospital on opioid replacement therapy (i.e., morphine, methadone, or buprenorphine). We define routine discharge/transfer as ≥10% of infants who receive opioid replacement therapy for NOWS at the site

4.1.2.2. Infant Level

1. Infant has major birth defect(s)
2. Infant has neonatal encephalopathy (inclusive of hypoxic ischemic encephalopathy), a metabolic disorder, stroke, intracranial hemorrhage, or meningitis diagnosed by 60 hours of life
3. Infant was receiving respiratory support (any positive pressure or oxygen therapy) unrelated to pharmacologic treatment for NOWS at 60 hours of life
4. Infant was receiving antimicrobial(s) at 60 hours of life
5. Infant has received any major surgical intervention in the first 60 hours of life
6. Postnatal opioid exposure other than for treatment of NOWS in the first 60 hours of life

7. Outborn infants transferred at >60 hours of life or treated with opioids for NOWS at the transferring hospital
8. Infant's biological mother or primary caregiver is positive or under investigation for COVID-19 at 60 hours of life

4.1.2.3. Additional Infant-level Exclusion Criteria for Consented Portion of the Study

1. Infant's primary caregiver does not speak, read or write English.

4.2. DETAILED STUDY PROCEDURES

Table 4 outlines study procedures from the initial hospital stay through 24 months.

Table 4 Study Event Schedule

Evaluation/Procedures	Hospital Stay	Hospital Discharge	1 month (post discharge)	3 months of age*	6 months of age*	9 months of age*	12 months of age*	18 months of age*	24 months of age*
Maternal and infant medical history	X								
Neonatal opioid withdrawal scoring/assessments	X								
Date/time of initiation and number of doses of opioid replacement therapy administered (Infant)	X								
Date/time of initiation dose and number of doses of adjuvant therapy administered (Infant)	X								
Date/time when medically ready for discharge (Infant)	X								
Date/time discharged (Infant)		X							
Weight, length and head circumference (Infant)	X	X							O
Feeding type (Infant)	X	X							
Seizures, accidental trauma (i.e., dropped infants) and respiratory insufficiency (Infant)	X	X							
Serious Adverse Events (Infant)	X	X							
Acute/urgent care and/or ER visits and hospital readmissions				X					
Non-accidental trauma & death (Infant)		X		X					X

Evaluation/Procedures	Hospital Stay	Hospital Discharge	1 month (post discharge)	3 months of age*	6 months of age*	9 months of age*	12 months of age*	18 months of age*	24 months of age*
Infant Behavioral Questionnaire (IBQ) – very short form (Infant)				O			O		
Caregiver questionnaire (CQ) (enteral feeds, acute/urgent care and/or ER visits and readmissions) (Infant)			O	O	O		O		O
Brief Infant Sleep Questionnaire (BISQ) (Infant)				O			O		
Patient Reported Outcome Measurement Information System (PROMIS) Short Forms (Caregiver(s))		O			O				O
Maternal Postnatal Attachment Questionnaire (MPAQ) (Caregiver(s))		O			O				
Parenting Sense of Competence Scale (PSOC) (Caregiver(s))		O			O				
Adverse Childhood Experiences (ACE) questionnaire									O
Family Environmental Scale (FES) – Relationship Dimension Form R (Caregiver(s))				O					
Bayley Scales of Infant and Toddler Development, Fourth Edition (Bayley-4): Cognitive, Language, Motor,									O
Brief Infant-Toddler Social and Emotional Assessment (BITSEA)									O
Contact information update	O	O	O	O	O	O	O	O	
	(X) Evaluations/procedures assessed under waiver of consent<br>(O) Evaluations/procedures performed with informed consent<br>*Procedures occurring at ≤12 months of age may occur within ±3 weeks of stated time point.<br>Procedures occurring at 18 months of age may occur within ±6 weeks of stated time point;<br>Procedures at 24 months of age may occur within ±8 weeks of stated time point.								

4.2.1. Screening

The protocol study team will screen interested sites for eligibility, and will randomize eligible sites into one of 8 blocks, as illustrated in Table 1. With this study design, all infants with NOWS cared for at a site, will be evaluated and managed using the care approach that the site is assigned to during the study period. Therefore, individual infant screening will not be required before initiation of this study protocol. The process will be as follows (see Figure 1), after birth the inpatient clinical team will assess infants as at risk for NOWS and initiate management for NOWS based on the site's usual methods of identification. The initiation of clinical management for infants with NOWS will not be impacted by the study intervention. The site research team will identify potential participants for the trial based on their eligibility following review of the medical record after delivery. The site research team may obtain informed consent for infant and caregiver participation in the long-term outcomes portion of the study at any point during the hospital stay for infants who meet the trial's inclusion criteria. The site research team will evaluate for exclusion criteria (Section 4.1) after the infant's first 60 hours of life. The site research team will note infants as screen fails who meet any of the exclusion criteria, and will not collect additional data for these infants. Sites may enroll up to 16 infants per period; screen fails are not included in this total. Only infants enrolled in the study may be approached for consent and included in the long-term follow-up study.

Figure 1 Screening and Enrollment Procedures

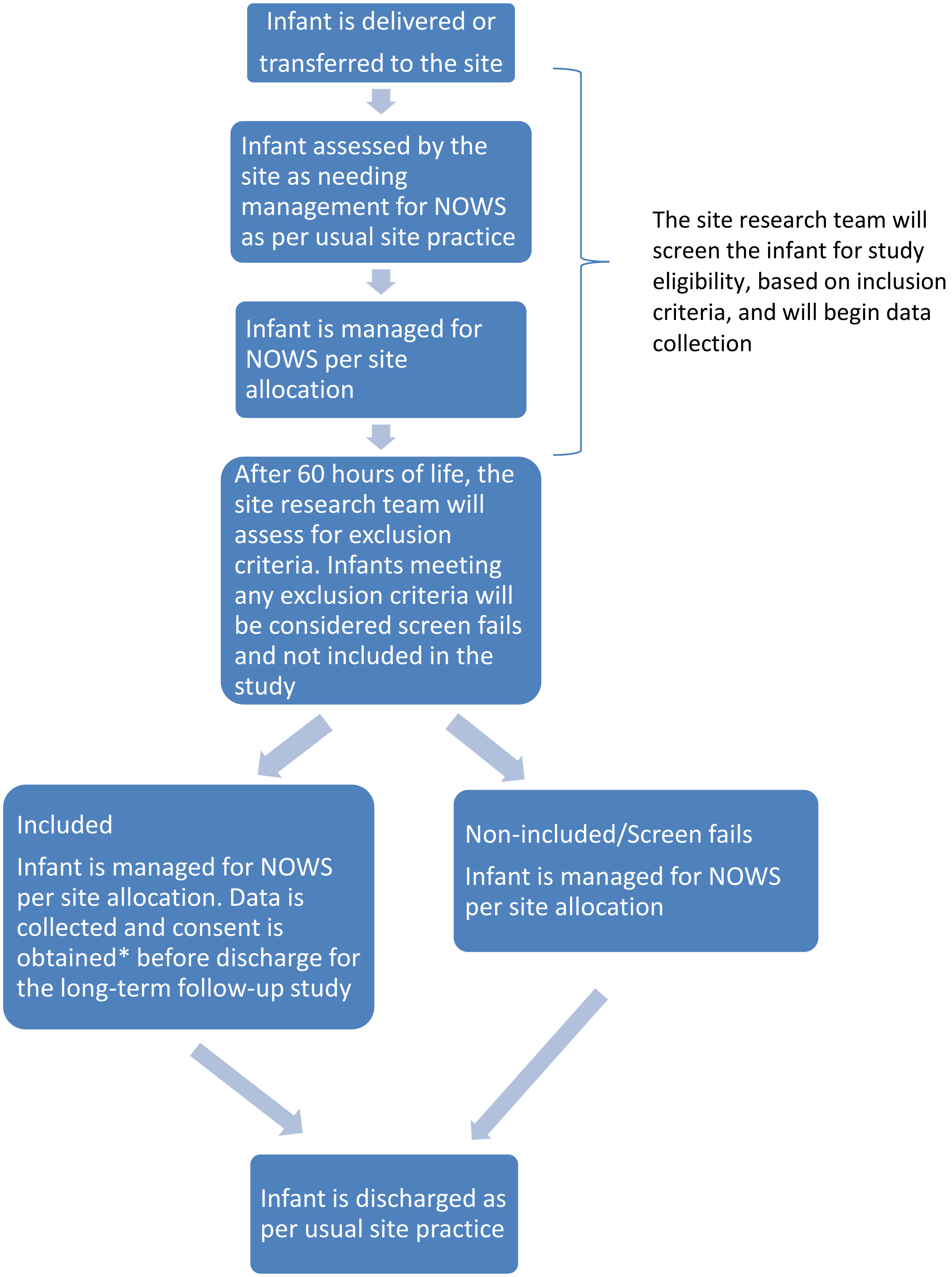

*The site research team may obtain consent for the long-term follow-up portion of the study at any point during the hospital stay for infants who meet the trial's inclusion criteria. To optimize recruitment, it will be permissible to obtain initial consent up to one month after discharge.

4.2.2. Consent Procedures

4.2.2.1. Waiver of Consent

Since this study is a stepped-wedge cluster randomized controlled trial, the intervention will take place on a site-wide basis and sites will transition their practice for all infants with NOWS cared for at the site during the study period. Thus, we will request a waiver of consent from the central institutional review board (IRB) at the University of Arkansas for Medical Sciences for the primary outcome and previously outlined short-term secondary outcomes. There is debate in the clinical trial and ethics literature about the issue of individual consent for cluster-randomized trials. However, in general, a study may proceed without individual consent if conditions for a waiver of consent are satisfied and participants (or legally authorized representatives) are provided with a description of the intervention to which their cluster has been randomized.[35]

As stated in Code of Federal Regulations [45 CFR 46.116 (d)], an IRB may approve a consent procedure that does not include, or that alters, some or all of the elements of informed consent set forth in this section, or waive the requirements to obtain informed consent, provided the IRB finds and documents that all of the following conditions are met:

1. The research involves no more than minimal risk to the participants;
2. The waiver or alteration will not adversely affect the rights and welfare of the participants;
3. The research could not practicably be carried out without the waiver or alteration; and
4. Whenever appropriate, the study team will provide participants with additional pertinent information after participation.

The justification for a waiver of informed consent from caregiver(s) for the previously outlined short-term outcomes (see Table 2) meets the above criteria per the following:

1. *The research involves no more than minimal risk to the participants.*

   Both usual care using the FNAST and the ESC care approaches are currently used at sites across the country, and the optimal care approach for the management of infants with NOWS is unknown. Additionally, there are no study procedures or study interventions within this protocol that would qualify as more than minimal risk, based on federal regulations, for either intervention group.

2. *The waiver or alteration will not adversely affect the rights and welfare of the participants*.

   As the best management for infants with NOWS is unknown, there is no universally accepted standard of care, and both care approaches are currently being used at sites across the country. Therefore, participants receiving care via either model should not have their rights and welfare adversely affected.

3. *The site research team could not practicably carry out this trial without the waiver or alteration.*

   Carrying out this trial and obtaining generalizable results would not be feasible if obtaining informed consent were required. Obtaining informed consent from legally authorized representatives of infants in this population is difficult due to multiple factors. The interventions conducted in this study begin shortly after birth and recruitment during this vulnerable period can be extremely difficult. Researchers have experienced this difficulty in a

number of trials that have failed to successfully recruit this population shortly after birth.[36, 37, 38] Seeking consent shortly after delivery may not only result in recruitment failure, but may result in achieving consent only among a less generalizable group of "responders", which may introduce bias and diminish generalizability. This could be particularly problematic in this trial where the success of the intervention may be particularly susceptible to caregiver effort and engagement.

Seeking consent later in the hospital stay for the long-term follow-up portion of the study will allow for relationship and trust building between the consenting member of the site research team and the primary caregiver(s). This will likely allow for improved consent rates and improved generalizability. If consenting members of the site research team sought early consent and only a group of "responders" were consented, it is unclear whether this would have a differential impact across the study interventions. Additionally, the intervention is instituted at the site level and will represent a culture change. The site will use the assigned approach to care for all infants with NOWS during the trial period. Therefore, if consent were required, obtaining consent would not alter the care approach used for the infant. Thus, the benefits afforded by using a waiver of consent outweigh the risks to the infant receiving the same management. If the clinical team used two different care models at the same time and at the same site, patient safety could be at risk and care potentially compromised due to the use of inconsistent care practices at the site.

4. *Whenever appropriate the study team will provide participants with additional pertinent information after participation.*

   Throughout the study, the site research team will provide participants with additional pertinent information when appropriate. The protocol study team will develop a handout that the site research team will (a) give to the caregiver(s) of all infants with NOWS cared for at the site throughout the study period and/or (b) post (as an enlarged copy) in appropriate location(s) readily visible to caregivers. This will fulfill the suggested framework[35] of participants being "provided with a detailed description of the interventions to which their cluster has been randomized."

4.2.2.2. <u>Consent for Assessment of Long-term Outcomes</u>

Members of the site research team will work with families/caregivers to obtain informed consent for: 1) parent/caregiver questionnaires that will assess caregiver well-being (e.g., parenting stress, attachment and bonding, depression, anxiety, etc.) and infant well-being (e.g., diet, sleep, neurobehavioral functioning, etc.), and 2) in-person follow-up visit at 24 months to assess neurodevelopmental outcomes and growth measures. Consent will contain basic information on recognition and support (consistent with regulatory requirements at each site) for mental health issues including suicidality among caregivers. The consent will also contain basic information on notification of child protective services (consistent with state law) should researchers or members of the clinical team have suspicion of child neglect or abuse. The site research team will obtain written, informed consent from primary caregiver(s) (e.g., biological parents, adoptive parents, or state-appointed guardians) prior to administration of the first questionnaire.

Per section, 4.5.2.1., *Recruitment*, participants may be consented up to 1 month after discharge. To facilitate this process, and due to the ongoing COVID-19 pandemic, remote consenting will be allowed.

All communications will be done via HIPAA-compliant methods such as telephone, personal delivery of documents, US postal service, REDCap or other compliant electronic platform. The remote consent process will parallel the consent processed used for in-person consenting. The only difference will be the method(s) of communication. The study team will ensure that, as with in-person consenting, the participant is given sufficient opportunity to ask questions, is able to understand the nature of this study and what participation entails, and is provided a copy of the final, completed consent signed by all parties involved, including the research team member who obtained consent and, when applicable, the site investigator. This final, signed consent will be provided via a HIPAA-compliant method or a method that the participant has agreed to in writing. The site research team members working on the consenting process will ensure that any participant who is consenting remotely has the authority to consent.

4.2.2.3. Detailing Barriers to Consent and Participation

The site research team will ask non-consenting parents/caregivers to answer questions specific to perceived or actual barriers to participation and their choice not to be involved in the long-term outcome portion of the study. The site research team will inform non-consenting parents/caregivers about the purpose of these questions and that they are not required to answer them. The site research team will record responses without linking identifiers. The protocol study team will not permit an amendment of the consent form for previously non-consenting parents/caregivers that wish to consent following these questions. The protocol study team will use the data collected to improve site-specific and study-wide recruitment strategies for this trial and to inform future trials in this field.

4.2.3. Randomization Procedures

This is a stepped-wedge cluster randomized design with a transition period wherein we will randomize participating study sites, rather than individual infants. All sites will implement the ESC care approach at some point during the trial; the random elements are two-fold: 1) randomization into the blocks, and 2) randomization of blocks to the time point at which each block implements the ESC care approach, the so-called “step” of the stepped-wedge design.

A statistician at the independent Data Coordinating Center (DCC) for the trial will generate a randomization list using SAS 9.4 (SAS Institute Inc., Cary, NC). The protocol study team will use the proportion of infants with NOWS treated pharmacologically at each site as the variable to stratify randomization (i.e., lowest 3rd, middle 3rd, highest 3rd). The protocol study team will identify this proportion using the results of the ACT NOW Current Experience Protocol, a retrospective data collection that details the inpatient identification, assessment, and management of infants with NOWS at the ISPCTN and participating NRN sites. The protocol study team will conduct a brief survey to obtain similar estimates from other interested sites. The protocol study team will randomize sites in each stratum into one of 8 blocks (Figure 2). Once the protocol study team randomizes each site into blocks, computer-generated random numbers from a uniform distribution will determine the order in which the block of sites step into the transition and implementation period for the ESC care approach. The DCC will hold the randomization list. Due to the nature of a stepped-wedge cluster randomized controlled trial, the protocol study team can only enforce limited blinding. The protocol study team will notify sites of their allocated block following randomization.

Integration of the ESC care approach is a complex process and training of the hospital staff will be time-intensive. Thus, we expect to observe no quantifiable effects on the outcomes of interest during the transition period. Hence, this study has a planned transition period. The site research team will collect data on primary and secondary outcomes for all study periods, excluding the transition period. As this trial has sufficient power, we have planned for the transition period to be 3 months in duration to allow for adequate time for training and implementation at the sites. To maintain this, the preceding usual care period and the initial ESC period will each be 1.5 months/6 weeks in duration.

Figure 2 Randomization Flow Diagram

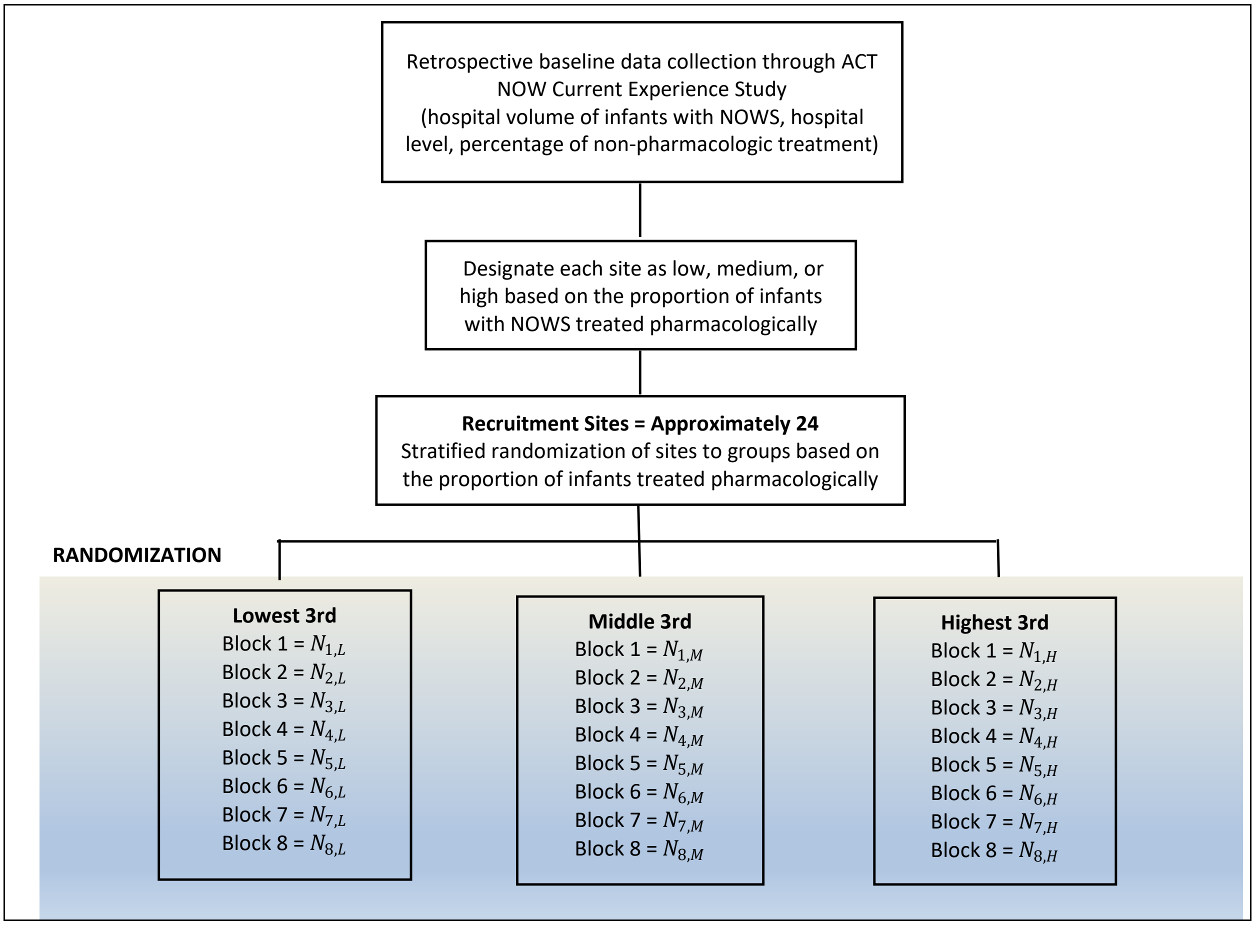

4.2.4. Study Intervention and Comparison

All sites will provide usual institutional care, including the use of the FNAST, or modification thereof, for the evaluation and management of infants with NOWS during Period 1 (see Table 1). After the first period, the sites in Block 1 (3 - 4 sites) will move into the transition period. During the transition period, sites will participate in education and training modules conducted through a centralized training platform. The protocol study team will standardize education and training across sites during each block's designated transition period. Educational modules will include an introduction and overview of the ESC care approach, education on trauma informed care and bias, and a general review on caring for infants with NOWS and the importance of non-pharmacologic care. Training will occur in a train-the-trainer format, and will include off-site or teleconference ESC training for a core group of site champions with subsequent on-site training of clinical personnel. Following the transition period, sites within Block 1 will move into the first ESC period and each of the other blocks will move into their next designated intervention period. The site research team will collect data during all intervention periods of the study and make comparisons between these interventions (usual care versus ESC care approach).

4.2.4.1. Usual Institutional Care Periods

Intervention

- Following delivery or transfer, the care team will initiate care for NOWS per usual practice. The clinical team will use institutional practices and protocols to guide non-pharmacologic care, to assess the infant using the FNAST or modification thereof, and to guide pharmacologic care.
- If needed, the clinical team will initiate pharmacologic treatment per the site's usual practice and/or treatment protocol, and escalation, weaning, and discontinuation of pharmacologic care will be per the site's usual care. Opioid replacement therapy given (morphine, methadone, or buprenorphine) will be per site preference, as will adjuvant therapy used (clonidine or phenobarbital).
- The clinical team will monitor each infant requiring opioid replacement therapy for signs of escalating withdrawal symptoms following discontinuation of this treatment and will consider discharge per the site's usual practice.
- The clinical team will use FNAST or modification thereof to assess infants after birth and consider discharge for infants who do not require pharmacologic treatment per the site's usual practice.
- The DCC will develop a monitoring plan for each site's compliance with usual care during this period.
- Infants with antenatal opioid exposure born or transferred to the site during the usual institutional care intervention will be managed per this care approach throughout their admission (this includes infants who remain admitted when the site enters the transition period), and the site research team will collect their data and use it for the study analysis.

4.2.4.2. Transition Periods

Intervention

Education

- All research and clinical nurses, advanced practice providers, and physicians who care for infants with NOWS at each participating site will complete educational modules. These modules will include an introduction and overview of the ESC care approach, education on trauma informed care and bias, and a general review specific to caring for infants with NOWS. The latter will include an emphasis on the importance of non-pharmacologic care, as well as on the importance of differentiating the etiology of symptoms common to NOWS.
- The protocol study team will assess completion of these modules.

Training and Implementation

- The protocol study team will conduct all training in a train-the-trainer format supported by nationally known clinical experts.
- The protocol study team will train a core group of site champions, which may include research and clinical nurses and physicians, in the use of the ESC care approach during the designated transition period. Education and training on the optimal use of the ESC care approach will

include an introduction and overview of the ESC care approach, review of the ESC Care Tool with definitions and Newborn Care Diary, ESC training video, and review of written and videotaped case scenarios. The site champions will access the components of this training through the educational platform, and the protocol study team will track completion through pre/post assessments. The protocol study team will provide an electronic copy of the ESC Training and Implementation Manual for each site in anticipation of entry into the transition period.

- After the training, the core group of site champions will view and score cases until each member of the group consistently attains 100% reliability on standardized patient assessment cases (6/6 items). We define this as three consecutive assessments with 100% reliability as compared to national experts in the field. Once consistently achieving 100% reliability, the protocol study team will consider these individuals the "gold-star raters."
- This core group will train all other clinical personnel who care for infants with NOWS at their site, including, but not limited to, nurses, advanced practice providers, and physicians in all areas where these infants receive care. These areas may include, but are not limited to, the well-baby nursery, pediatric unit, and neonatal intensive care unit. After the training, clinical personnel will view and assess cases using the ESC IRR tool (see Training & Implementation Manual) until clinical personnel consistently achieve 80% agreement (5/6 items). Once a member of the clinical team reaches 80% IRR, the site research team will clear the trainee for independent assessment. If the trainee consistently achieves 100% reliability, the site research team will consider him/her to be a “gold-star rater”, and may ask the trainee to function in this capacity. Site staffing levels will determine the number of “gold-star raters" at a site, with the goal of having one “gold-star rater” available on each shift. The protocol study team will require clinical personnel who are unable to attain 80% reliability to complete supplementary training. Clinical personnel hired after the initial training will complete the educational modules and ESC training at the site inclusive of assessing cases using the ESC IRR tool to demonstrate 80% agreement.
- To ensure fidelity of the assessments, the site research team will assess the reliability of “gold-star raters” at each site during the implementation phase of the transition period (see Training & Implementation Manual). The protocol study team anticipates each “gold-star rater” will maintain 100% reliability in scoring on patient assessment cases. The “gold-star raters” will then gauge reliability of the clinical team by assessing individuals during the implementation phase by using the ESC IRR tool and written or video case scenarios (see Training and Implementation Manual). For clinical personnel who fail to maintain the target of 80% reliability in scoring during the implementation phase of the transition period, the site research team will utilize just-in-time training through a centralized training platform until he/she achieves 80% reliability in assessments. When staffing allows, members of the care team who have reliability less than 80% should not be assigned to care for infants with NOWS until improved reliability is demonstrated through the just-in-time training process.
- Infants with antenatal opioid exposure born or transferred to the site during the transition period but before the site has implemented ESC, will be managed with usual institutional care. Once a site implements ESC, the site will manage all infants born or transferred to the site with

the ESC care approach. For those infants receiving ongoing care for NOWS at the time of ESC implementation, the protocol study team will leave the care approach used for the continued care of these infants to the discretion of the clinical team.

- Infants born or transferred to the site during the transition period will not have their data collected and these infants will not be included in the study analysis.
- Clinical leads from each discipline (i.e., nursing and medicine) and the site research team will assess for completeness of training milestones and the ESC care approach implementation prior to the site's formal movement into the ESC intervention period.
- ESC experts will conduct coaching calls for each block of sites through the transition and initial intervention period(s). These coaching calls will provide continued support to the sites during this initial period of implementation, and ESC experts will continue to conduct these coaching calls or educational meetings on a monthly basis throughout the subsequent ESC intervention period(s).

4.2.4.3. ESC Intervention Periods

Intervention

- After delivery or transfer to the site, the care team will initiate non-pharmacologic care for NOWS, as detailed in the ESC training materials, and non-pharmacologic care will remain in place for the full duration of the infant's management for NOWS.
- Non-pharmacologic care can include: primary caregiver(s) involvement (rooming-in if possible), promoting breastfeeding (for eligible infants based on the institution's established breastfeeding guideline), encouraging on-demand feeding, enhancement of low light and minimal noise exposure, supporting clustered care (doing assessments, vitals, and all other care around feeding, to promote sleep), swaddling, and skin-to-skin care by primary caregiver(s) or holding by family/staff volunteers.
- Not all sites will be able to offer all forms of non-pharmacologic care and not all infants will be able to receive all non-pharmacologic interventions available at the site. Acknowledging this, the clinical team will make every attempt to optimize the non-pharmacologic care provided to each infant.
- The clinical team will encourage primary caregiver(s) to participate in the care and evaluation of their infants. The clinical team will also encourage the primary caregiver(s) to record the infant's feedings (timing and duration, and/or volume), sleeping (quality and quantity), and ability to be consoled, in the Newborn Care Diary, a component of the ESC care approach.
- The clinical team, in collaboration with the primary caregiver(s), will use the ESC Care Tool to assess the infant with respect to the ESC items (eating, sleeping and consoling) by approximately 4 to 6 hours of life (if risk for NOWS is known) or upon identification of the need for NOWS management.
- The clinical team will perform ESC care assessments every 2 to 4 hours after feedings, clustering other infant and maternal care (i.e., vital signs) at the same time. These assessments will include a collaborative review with the primary caregiver(s) (when available) of the ESC items

since the last assessment, using the Newborn Care Diary. If the primary caregiver(s) are not available, the clinical team who participated in the care of the infant during the assessment period will complete the assessment.

- If during an assessment the infant has a "Yes" for any ESC item or obtains a score of "3" for "Consoling Support Needed" on the ESC Care Tool, the primary caregiver(s) and clinical team will conduct a "Parent/Caregiver huddle" to determine: 1) if the "Yes" is due to NOWS and 2) which non-pharmacologic care interventions the care team can optimize further. The "Parent/Caregiver Huddle" could include, but is not limited to, the parent/caregiver and the bedside nurse.
- If the care team can optimize non-pharmacologic interventions, they will do so and will continue to assess the infant.
- If it is unclear if the infant's difficulties with eating, sleeping, or consoling are due to NOWS, the care team will indicate a "Yes" on the ESC Care Tool and will continue to monitor the infant closely while optimizing all non-pharmacologic care interventions.
- If the infant has a second consecutive "Yes" for any ESC item (or "3" for "Consoling Support Needed") on the ESC Care Tool (or other significant concerns are present), despite maximal non-pharmacologic care, the care team will conduct a "Full-Care Team Huddle" to determine if: 1) the "Yes" is due to NOWS and 2) the infant needs pharmacologic treatment. A "Full-Care Team Huddle" could include, but is not limited to, the parent/caregiver and the bedside nurse, in addition to the physician and/or advanced practice providers caring for the infant.
- The clinical team will initiate pharmacologic treatment if the infant scores "Yes" due to NOWS on an ESC item or scores a "3" for "Consoling Support Needed" on the ESC Care Tool despite optimization of non-pharmacologic care. If an infant requires pharmacologic treatment, sites will initiate a treatment protocol to guide care. A treatment protocol should include dose initiation, escalation, and weaning parameters. Opioid replacement therapy given (morphine, methadone, or buprenorphine) will be per site preference, as will adjuvant therapy (clonidine or phenobarbital).
- The clinical team will monitor each infant requiring opioid replacement therapy for signs of escalating withdrawal symptoms following discontinuation of this treatment and will consider discharge per the site's usual practice.
- The clinical team will use the ESC Care Tool to monitor infants following birth and consider discharge for infants who do not require pharmacologic treatment per the site's usual practice.
- To ensure fidelity of the assessments, the site research team will randomly assess the reliability of "gold-star raters" at each site throughout the ESC intervention periods. The protocol study team anticipates that each "gold-star rater" will maintain 100% reliability in scoring. The "gold-star raters" will then assess reliability of the clinical team once per ESC intervention period, using the ESC IRR tool and a NOWS infant, written cases, or video cases. The protocol study team anticipates that each member of the clinical team will maintain 80% reliability in scoring. If a member of the clinical team fails to meet this target during the assessment, the site research team will utilize just-in-time training (see Training and Implementation Manual) until

the clinical team member achieves 80% reliability. The protocol study team would ask that members of the care team with reliability less than 80% not be assigned to care for infants with NOWS until improved reliability is demonstrated through the just-in-time training process.

- To ensure fidelity of ESC implementation, "gold-star raters" will discretely evaluate, in real time, how nurses implement the ESC care approach using the ESC Implementation Process Evaluation form (see Training and Implementation Manual). The protocol study team will use this tool to evaluate how consistent nurses are in their evaluation of infant symptoms, recommendations for care team huddles, as described by the ESC Care Tool, and implementation of the ESC Care Tool (inclusive of non-pharmacologic care interventions). The site research team will keep complete ESC Implementation Process Evaluation forms with other study documentation.

4.2.5. Protocol Adherence and Compliance Monitoring

The DCC will monitor protocol deviations per site in relation to the number of participants enrolled and visits conducted. All sites will receive re-education via regularly scheduled teleconferences to help other sites prevent similar deviations. If a particular deviation is recurrent at one site or across the sites, the DCC may implement operational tools, such as additional reminders, source document worksheets, and/or checklists, to reduce the likelihood of deviations. The DCC will review protocol deviations throughout the study, and it may schedule additional on-site visits, as needed, to review regulatory documents, data points, key issues, etc. or to retrain site staff to improve processes and provide additional education.

Strategies to improve or monitor adherence to the study protocol will include the following:

- Monthly recruitment reports of infants screened, enrolled, and consented (accrual figures)
- Screen fails will be reviewed by the protocol study team to assess for bias in inclusion/exclusion decisions
- Monthly reports detailing data received at the data center, data consistency, missing data, performance measures, and adherence to the study protocol (with appropriate measures taken to preserve the blinding of study personnel and investigators)
- Supplementary blinded reports requested by the study investigators or subcommittee that do not disclose allocation-group–specific outcomes (primary, secondary, or any safety outcomes)

The DCC will generate the aforementioned reports.

Additionally, the protocol study team will monitor protocol adherence through collection of the following data:

- Completion of modules and training by the research and clinical teams.
- Continual IRR fidelity assessments for the clinical team (reevaluated each period).
- Assessed adherence to the assigned care approach.

4.2.6. Post-hospital Procedures

The site research team will assess for the outpatient composite safety outcome at approximately 3 months of age as well as the critical safety outcome through review of the medical records (including the site's primary and any linked electronic medical record systems) and media review for all infants

enrolled in the study at approximately 3 and 24 months of age. Primary caregiver(s) for infants for whom the protocol study team obtained informed consent will receive questionnaires via electronic application or via phone interview, if caregiver(s) have limited access to cellular/internet service or prefer this modality of communication. Caregiver(s) will complete these questionnaires at discharge, 1-month post discharge, and 3 months, 6 months, 12 months, and 24 months of age. These questionnaires will gather information on infant neurobehavioral functioning, infant wellness, primary caregiver(s) well-being, family environment, and caregiver-infant interactions, as detailed in Section 4.3.2.2. In addition, there will be an in-person follow-up visit with neurodevelopmental assessment and anthropometric measures at 24 months of age. The site research team will maintain contact in between study assessments at regular intervals, as detailed in Table 4. As there will likely be differences between the populations who provide consent for follow-up and those who do not, we will collect socioeconomic data (insurance and maternal educational status), marital status, and maternal receipt of medication-assisted treatment for all populations to examine for possible bias.

4.2.7. Data Quality Assurance

To assure the quality of the data collected, the protocol study team will provide training specific to accuracy of data acquisition for the research coordinators at each site. The protocol study team will design data collection forms, which a subset of sites will subsequently pilot to minimize the potential for errors. Additionally, the protocol study team will allocate sufficient funds to allow for quality data collection. The site research team will re-abstract a subsample of their own charts and assess the error rate. Re-abstraction will focus on critical data elements related to the primary and secondary objectives of the protocol. The protocol study team will base the number of charts a site re-abstracts, for each 6-month interval, on the number of patients enrolled in the study during the 6-month period at each site as shown below:

No. of patients enrolled in a 6-month period	No. of charts to be re-abstracted
0	0
1-14	1
15-24	2
25-34	3
35-44	4
45-54	5
55-64	6

The DCC will provide sites with the randomly selected subject IDs for re-abstraction. The site research team will identify an independent site quality control (QC) abstractor who will re-abstract and enter data into the electronic data capture system (EDC) only for the QC process and will not abstract study data while QC activities are taking place. The DCC will generate a discrepancy report comparing study data abstracted by the site with the source information abstracted by the independent abstractor. The site manager will hold a QC Review Meeting with the independent site QC abstractor, research coordinator, and site abstractor(s) to review the discrepancies and identify errors. Together they will

discuss and document the corrective action for each error identified. The DCC will create manual queries in the EDC to make any necessary corrections to the data that QC Review members identify. The protocol study team will provide hospitals that have an error rate above the predefined threshold with additional training, a hospital-specific assessment of the data collection process, and suggestions for process improvement. The protocol study team will track hospitals by their error rates. The protocol study team will share practices of those hospitals with exceptionally low error rates with hospitals working to improve their own process. The protocol study team will review error rates and re-abstraction data during monthly team calls. If errors exceed the predefined threshold on 2 consecutive reviews, a remediation plan will be requested and shared with the study sponsor.

Sites that have an error rate above the predefined threshold will receive additional training, a site-specific assessment of the data collection process and suggestions for process improvement. The protocol study team will highlight sites with exceptionally low error rates, and these sites will share aspects of their data collection process with sites working to improve their own process.

4.2.8. Blinding/Masking

The protocol study team will assure blinding of the electronically performed follow-up questionnaires through the use of a centralized computer scoring system. For questionnaires completed by phone, each site should develop a site-specific protocol to preserve blinding of those administering the questionnaires. The protocol study team will note the method of questionnaire completion.

4.3. STUDY OBJECTIVES AND ENDPOINTS

4.3.1. Primary Outcome

The primary outcome is the time from birth until infants are medically ready for discharge. We define medically ready for discharge as when the infant meets ALL of the following criteria:

1. ≥ 96 hours of life
2. Off opioid replacement therapy (e.g. morphine, methadone, or buprenorphine) for ≥ 48 hours
3. Taking 100% of feeds by mouth for ≥ 24 hours
4. ≥ 24 hours from initiation of the maximum caloric density infant received during the initial hospital admission
5. Receiving no respiratory support for ≥ 24 hours

- Hypothesis: Among infants evaluated for NOWS, the ESC care approach will reduce the length of time until infants are medically ready for discharge by an average of 4 days, compared to usual institutional care with the FNAST or modification thereof.

4.3.2. Secondary Outcomes

4.3.2.1. Obtained Under Waiver of Consent and Gathered by Authorized Site Research Personnel from the On-Site Medical Records, Linked Medical Records and Research Forms

1. Receipt of opioid replacement therapy (morphine, methadone, or buprenorphine) for NOWS prior to hospital discharge

- Hypothesis: The use of the ESC care approach will decrease the proportion of infants who receive opioid replacement therapy.
- This is a yes/no outcome, and it will enable us to determine the percentage of infants receiving opioid replacement therapy in each intervention group.

2. Total postnatal opioid exposure prior to hospital discharge
    - Hypothesis: The use of the ESC care approach will decrease total opioid exposure, compared to usual care.
    - Each dose of opioid replacement therapy (total units and units/kg and morphine equivalents [mg/kg]) that infants received throughout the initial birth hospitalization will be collected to determine total postnatal opioid exposure.
3. Hour of life opioid replacement initiated
    - Hypothesis: The use of the ESC care approach will not delay the initiation of pharmacologic therapy.
    - Use of the ESC Care Tool for the assessment of infants may delay the initiation of pharmacologic therapy and thus infants may be at an advanced state of withdrawal and more difficult to "capture". Alternatively, there is some evidence to suggest[27] that use of the ESC Care Tool ultimately allows for more timely recognition of infants requiring pharmacologic therapy, compared to usual care using the FNAST.
4. Receipt of adjuvant therapy (clonidine or phenobarbital) prior to hospital discharge
    - Hypothesis: The use of the ESC care approach will decrease the proportion of infants who receive adjuvant therapy.
    - This is a yes/no outcome, and it will allow us to determine the percentage of infants receiving adjuvant therapy.
5. Maximum percent weight loss during the initial birth hospitalization
    - Hypothesis: Use of the ESC care approach will not result in more excessive weight loss than usual care.
    - Poor feeding and excessive weight loss are signs of suboptimal control of NOWS. Birth weight and daily weights (g) will be collected throughout the initial birth hospitalization to determine the impact of NOWS on growth, and the maximum percent weight loss will be calculated as:

$$\left[\frac{birthweight\ (g) - weight\ nadir(g)}{birthweight\ (g)}\right] x\ 100 = \max percent\ weight\ loss$$

6. Type of enteral feedings (exclusive maternal breastmilk, combination of formula and maternal breastmilk, exclusive formula feeding) at time of hospital discharge
    - Hypothesis: Use of the ESC care approach will increase the proportion of infants who receive maternal breastmilk at the time of discharge from the initial birth hospitalization.

- Studies have shown that the receipt of maternal breastmilk decreases withdrawal signs in infants in a dose-dependent fashion.[39, 40]
- The site research team will assess and collect the type of enteral feeding at the time of discharge from the initial birth hospitalization.

7. Direct breastfeeding at the time of hospital discharge
   - Hypothesis: Use of the ESC care approach will increase the proportion of mothers who directly breastfeed at the time of discharge from the initial birth hospitalization.
   - The site research team will assess and collect direct breastfeeding occurrences within 24 hours of the time of discharge from the initial birth hospitalization.
8. Length of hospital stay
   - Hypothesis: Infants managed with ESC will have a decrease in the LOS.
   - The site research team will report the LOS in addition to the length of time until infants are medically ready for discharge. The differences in these measures will allow the protocol study team to assess the impact of social factors on the length of hospitalization.
9. A composite measure of infant safety during the initial birth hospitalization (seizures, accidental trauma [i.e., dropped infants], and respiratory insufficiency due to opioid therapy, including documented apnea or need for respiratory support [positive pressure or supplemental oxygen])
   - Hypothesis: Infants managed using the ESC care approach will be safe during the initial birth hospitalization.
   - Use of the ESC care approach may delay initiation of pharmacologic therapy, which could result in an increase in withdrawal-related seizures. Therefore, monitoring for the presence or absence of seizures will help to build the safety profile for ESC.
   - Increased primary caregiver(s) involvement is thought to result from the ESC care approach. In this case, parent/caregiver skin-to-skin time and holding may increase, which could increase the risk of infants being dropped if primary caregiver(s) are fatigued and/or chemically impaired.
   - Use of the ESC care approach may delay initiation of pharmacologic therapy, which could result in the infant receiving a higher dose of opioid replacement therapy. Higher doses of opioids may increase the risk of respiratory insufficiency. Therefore, monitoring for respiratory insufficiency will help to build the safety profile for ESC.
10. A composite measure of critical infant safety outcomes during the initial birth hospitalization (non-accidental trauma and death)
   - Hypothesis: Infants managed using the ESC care approach will be safe during the initial birth hospitalization.
   - Use of the ESC care approach encourages parents/caregivers to provide extensive non-pharmacologic care and rooming-in. This may increase stress and fatigue and

lead to undesired caregiver-infant interactions. Inclusion of a critical composite safety outcome inclusive of non-accidental trauma and death will help to build the safety profile for ESC

11. A composite measure of infant safety during the first 3 months of life based on the presence or absence of acute/urgent care and/or ER visits and hospital readmissions
    - Hypothesis: Infants managed using the ESC care approach will be safe during the first 3 months of life.
    - Discharge of an infant earlier from the initial hospitalization and/or increased primary caregiver involvement during the initial hospitalization may increase the stress and fatigue experienced by the caregiver(s) and lead to increased risk for poor outcomes, and increased healthcare utilization.
12. A composite measure of critical safety outcomes based on the presence or absence of non-accidental trauma and death at discharge and during the first 3 and 24 months of life
    - Hypothesis: Infants managed using the ESC care approach will be safe during the first 3 and 24 months of life.
    - Infants with undertreated signs of withdrawal may be at increased risk for non-accidental trauma and death due to the potential for increased primary caregiver stress and fatigue during the hospital admission and following discharge. These infants may also fail to develop a bond with their primary caregiver(s) during the first months of life, which may further increase the risk for non-accidental trauma and death during the first two years of life.

4.3.2.2. Obtained for the Subpopulation who Provide Informed Consent and Acquired through Questionnaires

Assessed at various time points between discharge and 24 months of age (see Table 4).

1. Infant neurobehavioral functioning following discharge
    - Hypothesis: Infants managed using the ESC care approach will have improved infant neurobehavioral functioning when compared to usual care.
    - Assessed with Infant Behavior Questionnaire - Revised (IBQ-R) very short form at 3 and 12 months of age. The caregiver will complete the survey and it will be sent to a central location for review by the protocol study team
        - The IBQ–R is a well-established caregiver report measure of neurobehavioral functioning through assessment of temperament for infants between 3 and 12 months of age.[41] The questionnaire has demonstrated good internal consistency, reliability, and validity.[42, 43, 44, 45] The IBQ-R consists of 191 items and takes approximately 1 hour to complete which makes it impractical for this study. The very short form consists of 37 questions that measure surgency, negative affect, and effortful control of the infant caregiver. This form takes approximately 12 minutes to complete.[46] The very short form has been shown to have reliability and stability that are similar to the IBQ–R and other temperament measures.[46]

2. Infant wellness following discharge as independently assessed by:

- Anthropometric growth (weight, height, head circumference)
  - Hypothesis: Use of the ESC care approach will not impact growth long-term when compared to usual care.
  - Assessed with percentile measurements of weight, length, head circumference (HC), and weight for length on WHO growth curves. The research team will assess weight, length, head circumference and weight for length at hospital discharge and 24 months of age. The study team will calculate anthropometric z-scores at these time points, and will assess BMI at 24 months of age and calculate BMI-z.
- Sleep
  - Hypothesis: The infant's sleep will improve after use of the ESC care approach compared to usual care.
  - Assessed with the Brief Infant Sleep Questionnaire (BISQ)[47] at 3 and 12 months of age. The caregiver will complete the survey and it will be sent to a central location for review by the protocol study team.
- Enteral feeds during the first 6 months of life (exclusive maternal breastmilk, combination of maternal breastmilk and formula, or exclusive formula feeding)
  - Hypothesis: Use of the ESC care approach will increase the proportion of infants who receive maternal breastmilk following discharge compared to usual care.
  - Assessed with the Caregiver Questionnaire (CQ) at 1-month post hospital discharge, and 3, and 6 months of age. The caregiver will complete the questionnaire and it will be sent to a central location for review by the protocol study team.
- Direct breastfeeding during the first 6 months of life
  - Hypothesis: Use of the ESC care approach will increase the proportion of mothers who directly breastfeed following discharge compared to usual care.
  - Assessed with the CQ at 1-month post hospital discharge, and 3, and 6 months of age. The caregiver will complete the questionnaire and it will be sent to a central location for review by the protocol study team.
- Number of ER visits and/or acute/urgent care visits
  - Hypothesis: Use of the ESC care approach will not result in an increase in the number of ER or acute/urgent care visits compared to usual care.
  - Assessed at 1-month post hospital discharge, and 3, 6, 12, and 24 months of age via completion of the CQ and submission for review by a protocol study team. The site research team will also assess the site's electronic health record (EHR) and include any visits not reported, if observed.
- Readmissions
  - Hypothesis: Use of the ESC care approach will not result in an increase in the number of readmissions following initial hospital discharge compared to usual care.

- Assessed at 1-month post hospital discharge, and 3, 6, 12, and 24 months of age via completion of the CQ and reviewed by the protocol study team. The site research team will also assess the sites' EHR and include any visits not reported, if observed.

3. Maternal/caregiver well-being

- Hypothesis: Use of the ESC care approach will improve maternal/caregiver well-being compared to usual care.
- Assessed with Patient Reported Outcomes Measurement Information System (PROMIS) short forms at discharge, 6 months and 24 months. [48] Standardized short forms examining mental health, specifically the areas of anxiety (PROMIS Short Form v1.0 - Anxiety - 8a 31May2019), depression (PROMIS_SF_v1.0_-_ED-Depression_8a_5-31-2019), anger (PROMIS Short Form v1.1 - Anger - 5a 27Apr2016), life meaning and purpose (PROMIS Short Form v1.0 - Meaning and Purpose - 8a 18Jul2017), and social support (PROMIS v2.0 - Emotional Support Short Form 4a 23June2016) will be completed by the primary caregiver and will be sent to a central location for review by the protocol study team.
- The standardized short form for each of the PROMIS Measures consists of between four to eight 5-point Likert scale questions. The PROMIS Depression Short form has been validated in the postpartum period and has been found to be strongly correlated with the Edinburgh Postnatal Depression Scale, the most extensively studied measure of depression in the postpartum period. [49, 50] In addition, the PROMIS anxiety measure has been correlated with the Mood and Anxiety Questionnaire (MASQ) and has been shown to be a valid measurement tool for anxiety in the post-partum period in a sample of parents whose infants were hospitalized in the NICU. [50] Administration takes approximately 10 minutes and includes a total of 33 questions.

4. Infant-caregiver bonding and attachment

- Hypothesis: Use of the ESC care approach will result in improved infant-caregiver bonding and attachment, compared to usual care.
- The protocol study team will assess with the Maternal Postnatal Attachment Questionnaire (MPAQ) at discharge and 6 months of age. The caregiver will complete the questionnaire and it will be sent to a central location for review by the protocol study team.
- Primary caregiver-infant interactions will be assessed with the MPAQ,[51] a 19-item questionnaire that assesses quality of bonding, absence of hostility, and pleasure in interaction. The MPAQ requires approximately 5 minutes to complete, and researchers have validated the tool for postpartum women with substance-abuse problems.[52]
- The focus of the MPAQ is primarily upon the caregiver(s) subjective experiences in relation to their infant in the first year of life.[53] Established risk quartiles exist, and the protocol study team will note caregiver(s) for entry and exit into these high-risk quartiles at each time point.

5. Parenting efficacy
   - Hypothesis: Use of the ESC care approach will result in improved caregiver sense of competency in caring for their infants compared to usual care.
   - The protocol study team will assess with the Parenting Sense of Competence (PSOC) Scale at discharge and 6 months of age. The caregiver will complete the questionnaire and it will be sent to a central location for review by the protocol study team.
   - The PSOC is a self-reporting instrument that measures and assesses parent self-efficacy. It is a 17-item publicly available scale that measures satisfaction (degree of liking a person has for their role as a parent) and efficacy (an individual's perceived competence in their role as a parent).
   - Researchers have used this tool to assess the impact of parenting efficacy on the likelihood of out-of-home placement and loss of custody in mothers with mental health and substance use disorders.[54]
6. Family environment
   - Hypothesis: Use of the ESC care approach will enhance family environment when compared to usual care.
   - The protocol study team will assess with Family Environmental Scale (FES) - Relationship Dimension - Form R at 3 months of age. The caregiver will complete the questionnaire and it will be sent to a central location for review by the protocol study team.
   - The Relationship dimension of the FES consists of the Cohesion, Expressiveness, and Conflict subscales. Form R for each subscale is composed of 9 true-false items.
   - The relationship dimension assesses the degree of commitment, help, and support that family members provide each other, the extent to which family members are encouraged to act openly and to express their feelings directly, and the amount of openly expressed anger, aggression, and conflict among family members.[55]
   - Researchers frequently use the FES to assess the home environment and it has been found to have strong psychometric properties.[55]
7. Influence of maternal childhood experiences on infant outcomes
   - Hypothesis: Maternal history of adverse childhood experiences will be associated with worse infant behavioral functioning and developmental outcomes.
   - The protocol study team will assess adverse childhood experiences using the Adverse Childhood Experience (ACE) Questionnaire at 24 months of age.
     - The ACE[56] is a self-report measure used to capture specific childhood experiences correlating with future social risk factors and negative health outcomes.

4.3.2.3. Obtained for the Subpopulation who Provide Informed Consent and Acquired through an In-person Visit at 24 months of age

1. Infant development
   - Hypothesis: Use of the ESC care approach will improve infant development, compared to usual care.
   - The protocol study team will assess infant development with the Bayley Scales of Infant and Toddler Development, Fourth Edition (Bayley-4): Cognitive, Language, and Motor, at 24 months of age. The Bayley-4 will be administered by a trained examiner blinded to neonatal history who has undergone extensive training and been certified to perform exams.
     - The Bayley Scales are recognized internationally as one of the most comprehensive tools to assess developmental outcomes in children. With the Bayley-4, it is even possible to obtain detailed information from non-verbal children as to their functioning. Children are assessed with 3 key developmental domains: cognition, language, and motor. Reliability and validity of the previous version of the instrument have been well established.[57]
   - The protocol study team will assess infant behavioral development with the Brief Infant-Toddler Social and Emotional Assessment (BITSEA) at 24 months of age. The BITSEA is a standardized and normed referenced instrument designed to assess for social-emotional, behavior concerns, and social competence in infants-toddlers. [58]
     - The 42-item parent rating form is a shorted version of the Infant-Toddler Social and Emotional Assessment. Scores include ratings of internalizing behaviors, externalizing behaviors, executive function (dysregulation), psychosocial competence, social relatedness; maladaptive, and atypical behavior. The behavioral indices included in the BITSEA have been observed to correspond with neurodevelopmental functioning among infants at risk for neurodevelopmental problems.

4.4. POTENTIAL RISKS AND BENEFITS TO PARTICIPANTS

Under the proposed study design, the protocol study team will randomize each site into blocks with each block transitioning from usual care to ESC at a randomly allocated time. At any given time during the study enrollment period, all infants managed for NOWS at a site will receive care consistent with the care approach assigned by the protocol study team. Sites throughout the country are currently using both care approaches described in this study for the evaluation and management of NOWS. Use of either care approach will not expose infants in this study to risk beyond that of usual/accepted clinical care.

Involvement in the study will not increase the risk to the family of legal ramifications associated with the *in utero* opioid exposure of their infants, as only infants who have been identified by the site as at risk for NOWS and for whom management for NOWS has begun, will be screened for enrollment in the trial. There will be no additional toxicology screening (maternal or infant) performed beyond what

medical professionals would typically obtain as part of usual institutional care at the site. Thus, there will be no additional information garnered with respect to substance use during pregnancy due to one's involvement in the trial.

The protocol study team will assess primary caregiver well-being (e.g. parenting stress, attachment and bonding, depression, anxiety, etc.) as well as infant well-being, neurobehavioral functioning, and development during the follow-up portion of the study.

The protocol study team will assess primary caregiver wellness with 5 PROMIS Measures (see 4.3.2.2). It is possible that these questionnaires may reveal that the primary caregiver is experiencing psychological distress potentially requiring support. The study team has determined that a standardized scoring threshold for the PROMIS Depression Measure will be used to identify these individuals. As thresholds specific to postpartum women with opioid dependency have yet to be established and given that severe depression (a t-score >70, or 2 standard deviations above the mean for the normative population is the threshold for severe depressive symptoms. [59] [60]) is most likely to impact family well-being, a score of >70 was chosen for this threshold.

If a primary caregiver has a t-score >70 on the PROMIS Depression measure, the primary caregiver will be provided with national hotline support numbers within the electronic questionnaire platform. In addition, after the questionnaire is completed in REDCap an email will be automatically generated and sent to the study coordinator and PI. Each site will develop a plan to provide support for the primary caregivers at risk and connect them with local mental health resources in response to those emails. The protocol study team will collect a copy of this plan from each site.

SAMHSA NATIONAL HELP LINE – 1-800-662-4357 (HELP)

- https://www.samhsa.gov/find-help/national-helpline
- SAMHSA's National Helpline is a free, confidential, 24/7, 365-day-a-year treatment referral and information service (in English and Spanish) for individuals and families facing mental and/or substance use disorders.

NATIONAL DOMESTIC VIOLENCE HOTLINE - 1–800–799–7233 (SAFE)

- https://www.thehotline.org
- Advocates are available 24/7/365 to talk confidentially with anyone experiencing domestic violence, seeking resources or information, or questioning unhealthy aspects of their relationship.

NATIONAL SUICIDE PREVENTION LIFELINE – 1-800-273-8255

- https://suicidepreventionlifeline.org
- The National Suicide Prevention Lifeline is a national network of local crisis centers that provides free and confidential emotional support to people in suicidal crisis or emotional distress 24 hours a day, 7 days a week.

Additionally, a response plan will be in place at each site for questions specific to incidental findings of or suspicions for child abuse and/or neglect.

Participants recognized to have neurodevelopmental impairment on the Bayley-4 exam will be referred to their primary care providers for follow-up. The study team will communicate and share the report with the caregiver(s) and primary care providers if requested by the participants' caregiver(s) and consent is obtained.

The infants in the study may not benefit directly from participation. There may be a benefit to the infant of information garnered from the developmental screening portion of the study. By virtue of inclusion in a research study, participants are at risk of loss of confidentiality of medical-record information because participants will have their medical records reviewed by research personnel. The protocol study team will institute measures to protect the privacy of medical information, including the coding of all HIPAA (Health Insurance Portability and Accountability Act) identifiers in medical records, limitation of access to the medical records to research personnel, and removal of any individual identifiers in reports and publications generated from the study. Research personnel will keep any hard copies of research records in a locked cabinet and will destroy these records after the study is complete and the protocol study team publishes the results. In this study, infants themselves are the primary research focus, thus justifying the inclusion of children. The protocol study team will not exclude a subject based on race, ethnicity, or gender. However, some of the study questionnaires that will be used have not been validated in languages other than English. Thus, the population for the consented portion of the study will be limited to infants of English-speaking, reading and writing caregivers. Due to the demographic distribution of NOWS, the proportion of low socioeconomic-status infants will likely be higher than in the general population.

4.5. RECRUITMENT AND RETENTION

4.5.1. Site Recruitment and Retention

The protocol study team began to optimize the potential for recruitment during initial protocol development, through an assessment of potential ISPCTN and NRN sites' willingness to participate in and enthusiasm for various study designs. The study design chosen for this protocol incorporates the feedback from these sites. During the site assessment process, the protocol study team will expect each site to commit in writing to the site's participation in and completion of the trial with maintenance of the site's allocated intervention for the duration of the study. The protocol study team will facilitate retention of sites through the focused allocation of funds to support participation, through assessment of needs, provision of support, and troubleshooting at each site, as needed.

4.5.2. Infant and Parent/Caregiver Recruitment and Retention

The site research teams will need to obtain participant consent for the long-term follow-up portion of the study. Historically, enrollment of infants with NOWS in clinical trials that seek to improve their care has been challenging. In response to this, the protocol study team plans to utilize a robust recruitment and retention plan developed to support and optimize the participation of this population in the follow-up portion of this study.

4.5.2.1. <u>Recruitment</u>

The single most important element of the recruitment strategy is to establish trust with the primary caregiver(s) and provide an introduction to the research plan prior to delivery. The prenatal consultation is most likely the first time that the family will meet the site PI or designee and is an ideal

time to introduce the trial. The consultation is the opportunity for the provider to gain trust with the family and reaffirm a partnership with the family. The consultation will include establishing a foundation of knowledge about NOWS, outlining gaps in current national care, and a detailed description of the research approach.

In anticipation that prenatal consultations will not be feasible for all patients, effective dissemination of information regarding the clinical trial will be exceptionally important. The protocol study team will provide an informational pamphlet to all parent/caregiver(s) of infants receiving care for NOWS at participating sites soon after delivery. The consenting member of the site research team will begin trust building with the parent/caregiver(s) in anticipation of the consenting process. The site research team will present information about the study in person, and/or via an informational brochure developed by the protocol study team and distributed to the sites. To further optimize recruitment, if informed consent is not able to be obtained during the initial hospitalization, it is permissible to obtain consent up to one month after discharge.

The protocol study team will assess site recruitment for long-term follow-up each month following site enrollment. If the protocol study team assesses the site as below target, the study team will evaluate the site's processes for recruitment, and the site will receive additional training and/or modifications to the recruitment approach as suggested by a recruitment and retention expert from the protocol study team.

Additionally, the protocol study team will assess for barriers to participation, perceived or actual, of non-consenters (see Section 4.2.2) and utilize their responses to further improve site-specific and study-wide recruitment strategies.

4.5.2.2. Retention

The protocol study team will optimize infant and parent/caregiver(s) retention soon after a site obtains consent, by sending a note of thanks to the parent/caregiver(s) and acknowledging the importance of their contribution to the future care we provide these infants. The site research team will further optimize retention via text messaging for reminders, and access to questionnaires through a centrally located electronic platform. The site research team will use the electronic health record to update a participant's contact information as needed, in the event that the contact information provided by the participant is not sufficient. The site research team can conduct questionnaires via phone interview if the caregiver(s) has limited access to cellular/internet service or prefer this modality of communication. If a participant answers questions for questionnaires or comes to an in-person visit, the parent/caregiver(s) will receive compensation for their time. This compensation will be provided at – or very near – the time the participant finishes that contact time.

Participants will be reimbursed for their time according to the following tables.

For those parents/guardians signing the primary consent (parent/guardian + infant):

Contact Time	Participated in	Reimbursement/ Compensation Amount
Hospital Discharge	Answering Questionnaires	$50.
1-month post discharge (of baby from hospital)	Answering Questionnaires	$50.
Baby 3 months of age	Answering Questionnaires	$50.
Baby 6 months of age	Answering Questionnaires	$50.
Baby 12 months of age	Answering Questionnaires	$50.
Baby 24 months of age	Bringing baby in for in-person Bayley's exam and answering questionnaires	$150.*

* If the parent/guardian participant answers the 24-month questionnaires but does not bring their child in for the 24-month in-person visit, the parent/guardian will receive only $50.00 for the 24-month time-point.

For those parents/guardians signing the infant-only consent:

Contact Time	Participated in	Reimbursement/ Compensation Amount
Hospital Discharge	n/a	n/a
1-month post discharge (of baby from hospital)	Answering Infant/child-specific Questionnaires	$50.
Baby 3 months of age	Answering Infant/child-specific Questionnaires	$25.
Baby 6 months of age	Answering Infant/child-specific Questionnaires	$25.
Baby 12 months of age	Answering Infant/child-specific Questionnaires	$50.
Baby 24 months of age	Bringing baby in for in-person Bayley's exam and answering Infant/child questionnaires	$130.*

* If the parent/guardian participant answers the 24-month infant/child-only questionnaires but does not bring their child in for the 24-month in-person visit, the parent/guardian will receive only $30 for the 24-month time-point.

For those parents/guardians/caregiver signing the caregiver-only consent:

Contact Time	Participated in	Reimbursement/ Compensation Amount
Hospital Discharge	Answering caregiver-specific Questionnaires	$50.
1-month post discharge (of baby from hospital)	n/a	n/a
Baby 3 months of age	Answering caregiver-specific Questionnaires	$25.
Baby 6 months of age	Answering caregiver-specific Questionnaires	$25.
Baby 12 months of age	n/a	n/a
Baby 24 months of age	Answering caregiver-specific Questionnaires	$20.

The mechanism of payment (gift card, check, etc.) will be site specific and will be according to each site's mechanism for making such payments.

The site research team will provide text reminders to the parent/caregiver(s) to optimize timely completion of the questionnaires. Additionally, the site research team at each site will include a retention coordinator, and the protocol study team will allocate funds to support this role.

Additionally, the protocol study team will explore other methods to optimize both recruitment and retention. This could include, but is not limited to, discussions with stakeholders and parent/caregiver(s) from the community who have had infants treated for NOWS and understand the importance of being able to successfully complete this trial.

SECTION 5. ANALYTICAL PLAN

5.1. STATISTICAL ANALYSIS PLAN

5.1.1. General Approach

The material of this section is the basis for the statistical analysis plan of this study. The protocol study team may revise the plan during the study to accommodate clinical trial protocol amendments and to make changes to adapt to unexpected issues in study execution and data that affect planned analyses. The protocol study team will conduct all statistical analyses following the statistical principles for clinical trials as specified in International Council on Harmonization Topic E9. The protocol study team will describe and justify any deviations from the planned analyses in the final integrated clinical study report. The protocol study team will present overall and study site-specific data and summary tables.

The protocol study team will present the characteristics of infants and mothers by intervention groups (usual care versus ESC care approach) and their outcomes for each site. We do not expect significant differences in the demographics of the study population during the 20-month study period. Each site covers a different population mix, and while each hospital will contribute both usual care and ESC participants, they will do so in different proportions depending on when the protocol study team randomizes the hospital to the intervention. This will contribute greatly to any demographic differences between the usual institutional care and ESC groups. Whilst we do not intend to test for demographic differences between the usual institutional care and ESC groups for the full cohort, we will adjust the analyses for the covariates described because of potential imbalance across sites and across steps. We will present numerical variables as means [standard deviation (SD)] or medians (interquartile range), depending on their distribution, and categorical variables as counts and percentages.

We will use the principles of intention-to-treat for all statistical analyses related to primary and secondary endpoints.

5.1.2. Analysis of the Primary Efficacy Endpoints

For the primary efficacy variable, we will test the following null hypothesis:

H_0: There is no treatment difference in average length of time until medically ready for discharge between usual care and the ESC care approach.

Versus

H_1: There is a treatment difference in average length of time until medically ready for discharge between usual care and the ESC care approach.

We will consider the length of time until medically ready for discharge measure a count measure and has the potential to follow a skewed distribution. Initially, we will assess the distributional assumption. We will evaluate the associations of potential confounders (e.g., gestational age, birth weight, race/ethnicity, hospital volume, rural/urban indicator) at both the participant and site level with the intervention. An additional potential confounder that we will evaluate will be the presence of other ongoing clinical trials in our trial sites that might impact the outcome of this study, including the "Prospective Randomized Blinded Trial to Shorten Pharmacologic Treatment of Newborns with Neonatal Opioid Withdrawal Syndrome (NOWS)".

We will use a generalized linear mixed-effects model (GLMM) to compare the expected length of time until medically ready for discharge between the two treatment interventions (usual care and ESC care approach). Specifically, we will use a GLMM with a negative binomial distribution and log-link to account for potential over-dispersion, as an infant level analysis, and accounting for correlations between observations in the same hospital by including hospital in the model as a random effect. We will report point estimates for the group mean difference along with a 95% confidence interval (CI). The model-building approach for our primary outcome will follow four analyses steps: 1) an unadjusted before/after of the effect of the ESC care approach (ignoring period/time effect); 2) the time period (i.e., steps) to examine if any potential intervention effect relates only to the intervention or also to an independent effect of calendar time; 3) an adjustment for infant-level and maternal characteristics and potential hospital-level confounders, such as hospital volume and rural/urban indicator; 4) the possible interaction between period and intervention effect. The impact of the ESC care approach on the primary outcome could potentially change over time, as the improvement in outcome could increase with time as the staff gains experience. However, the impact could also decrease after an initial improvement as the level of initial enthusiasm decreases. We aim to explore this question through the inclusion of an interaction between period/time and intervention effect in Model 4.

In certain circumstances, medical personnel may discharge an infant prior to being medically ready for discharge as defined in our protocol (e.g., sent home on opioids such as methadone, morphine, or buprenorphine). Therefore, to compare the 2 interventions based on the primary outcome, we will censor these infants. Since one can view the time until medically ready for discharge as a time-to-event outcome, we will use the log-rank test adjusted for a cluster randomized design to compare the median time the infant is medically ready for discharge between the intervention groups.[61] Additionally, we will use a Cox proportional hazards (Cox PH) model with the Lin and Wei robust sandwich estimate of the variance-covariance matrix, to account for clustering, to adjust for infant and maternal demographics. We expect the amount of censoring to be minimal, therefore the results from the Cox PH model will serve as a sensitivity analysis for our primary analysis based on a GLMM with log-link.

5.1.3. Analysis of the Secondary Endpoints Obtained Under Waiver of Consent

5.1.3.1. Receipt of Opioid Replacement Therapy (Morphine, Methadone, or Buprenorphine) for Neonatal Opioid Withdrawal Syndrome Prior to Hospital Discharge

The analysis team will compare the proportion of receipt of opioid replacement therapy for NOWS prior to hospital discharge between the intervention groups using a GLMM with a logistic link function. We will follow the same four modeling strategies described for the primary outcome. We will also present odds ratio estimate of receipt of opioid replacement therapy for NOWS for the intervention effect (ESC versus usual institutional care) with 95% CI.

5.1.3.2. Total Opioid Exposure Prior to Hospital Discharge

The analysis team will provide the median and range of the total opioid exposure prior to hospital discharge for each treatment group. For the unadjusted analysis, the team will compare the median opioid exposure of the treatment groups using the Wilcoxon rank-sum test for clustered data proposed by Rosner, Glynn, and Lee (2003).[62] Their test statistic extends the Wilcoxon rank-sum test under the assumptions that all participants from the same cluster belong to the same treatment group, that observations within any cluster are exchangeable, and that the intracluster dependence does not vary

across treatment groups. Additionally, the team will use median mixed regression to account for the potential skewness of maximum dose of opioid replacement therapy and of clustered data and allow adjustment for covariates. The team will use the same four model building sequence described for the primary outcome except that the team will replace GLMM with a median mixed regression model.

5.1.3.3. Hour of Life Opioid Replacement Initiated

The analysis team will provide median and range for the hour of life when opioid replacement was initiated, and will do so separately for each treatment group. We anticipate that most of the infants will not receive opioid replacement, therefore we will use a hurdle model to model the expected hour of life until medical personnel initiate opioid replacement (i.e., count data) while handling excess zeros and over dispersion. More specifically, the team will fit the first part of the model with a binary logit model, which models whether an infant receives opioid replacement or not. In the second part, the team will utilize a negative binomial mixed model to account for the stepped-wedge design and adjust for potential infant and maternal demographics.

5.1.3.4. Receipt of Adjuvant Therapy (Clonidine or Phenobarbital) Prior to Hospital Discharge

The analysis team will compare the proportion of receipt of adjuvant therapy between the treatment groups using a GLMM with a logistic link function. We will follow the same four modeling strategies described for the primary outcome. We will present odds ratio estimate of receipt of adjuvant therapy for the intervention effect (ESC versus usual institutional care) with 95% CI.

5.1.3.5. Maximum Percent Weight Loss during Birth Hospitalization

The analysis team will provide the mean and SD of percent weight loss during birth hospitalization separately for each treatment group. The team will use a GLMM with an identity link function to compare average percent weight loss between the ESC care approach versus usual institutional care. The analysis team will report point estimates for the group mean difference along with a 95% CI. The team will use the same four model building sequence described for the primary outcome.

5.1.3.6. Type of Enteral Feedings (exclusive maternal breastmilk/breastfeeding, combination of maternal breastmilk and formula, exclusive formula feeding) at Time of Hospital Discharge

The analysis team will compare the proportion of infants receiving any maternal breastmilk (i.e., exclusive breastmilk/breastfeeding or combination) at discharge between the treatment groups using a GLMM with a logistic link function. We will follow the same four modeling strategies described for the primary outcome, and we will present the odds ratio estimate of receiving any maternal breastmilk for the intervention effect (ESC versus usual institutional care) with 95% CI.

5.1.3.7. Breastfeeding at the Time of Hospital Discharge

The analysis team will compare the proportion of breastfeeding at the time of hospital discharge between the treatment groups using a GLMM with a logistic link function. We will follow the same four modeling strategies described for the primary outcome. We will present odds ratio estimate of breastfeeding at the time of hospital discharge for the intervention effect (ESC versus usual institutional care) with 95% CI.

5.1.3.8. Length of Hospital Stay

Similar to the primary outcome (i.e., length of time until medically ready for discharge measure), we will consider LOS a count measure. Therefore, we will complete the analysis of LOS using a GLMM with log link assuming a negative binomial distribution to account for over-dispersion. The protocol study team will report point estimates for the group mean difference along with a 95% CI. Similar to the primary analysis, we will start with an unadjusted analysis and conclude with a model that includes possible interaction between period and intervention effect.

5.1.3.9. Composite Measure of Infant Safety during Birth Hospitalization (seizures, accidental trauma [i.e., dropped infants], respiratory insufficiency due to opioid therapy, including documented apnea or need for respiratory support [positive pressure or supplemental oxygen])

We will be monitoring for the presence or absence of safety indicators such as seizures, accidental trauma, and respiratory insufficiency due to opioid therapy. To assess the safety concerns of the ESC care approach, we will create a binary composite measure of inpatient infant safety. The binary composite measure will have a value of 1 if there is a presence for any inpatient infant safety indicator and 0 otherwise. We will compare the proportion of positive inpatient safety concerns between the treatment groups using a GLMM with a logistic link function. We will follow the same four modeling strategies described for the primary outcome, and we will present odds ratio estimate of inpatient safety concerns for the intervention effect (ESC versus usual institutional care) with 95% CI.

5.1.3.10. Composite Measure of Infant Safety during the First 3 Months of Life (acute/urgent care and/or ER visits and readmissions)

To assess the safety concerns of the ESC care approach, we will create a second composite measure consisting of outpatient infant safety indicators. We will base this outpatient composite measure on the presence or absence of acute/urgent care and/or ER visits, or readmissions during the first 3 months of life. Similar to the inpatient composite safety measure found in Section 5.1.3.9, we will compare the proportion of positive outpatient safety concerns between the treatment groups using a GLMM with a logistic link function. We will follow the same four modeling strategies described for the primary outcome, and we will present odds ratio estimate of outpatient safety concerns for the intervention effect (ESC versus usual institutional care) with 95% CI.

5.1.3.11. Composite Measure of Critical Safety Outcomes during the First 24 Months of Life (non-accidental trauma and death)

The analysis team will compare the proportion of non-accidental trauma and death between the treatment groups using a GLMM with a logistic link function. We will follow the same four modeling strategies described for the primary outcome, and we will present odds ratio estimate of non-accidental trauma and death for the intervention effect (ESC versus usual institutional care) with 95% CI.

5.1.4. Analysis of the Long-term Outcome Endpoints

5.1.4.1. Infant Wellness after Discharge under Provision of Consent

Growth Assessed with Respect to Weight, Length, Head Circumference, and Weight-for-length Normalized to World Health Organization Growth Curves

We will calculate anthropometric z-scores at each assessment period for the purpose of analysis based on age- and gender-specific WHO norms. The analysis team will provide the mean and SD of infants' weights (z-scores) separately for each treatment group. The team will use a GLMM with appropriate link function (i.e., identity link for continuous outcome) to evaluate the effect of ESC on weight (z-scores). The model will examine the how the treatment means differ (i.e., main treatment effect), how treatment means change over time (i.e., main time effect), and how differences between treatment means change over time (i.e., treatment-by-time effect). The team will carry out assessment across 2 time points: hospital discharge and 24 months of age. The GLMM analytical approach allows us to analyze correlated data obtained repeatedly from the same participant and account for the ICC among participants nested within with same clinical site. To account for potential imbalance in key demographic and site-level characteristics, the analysis team will utilize both unadjusted and adjusted GLMMs. Initially, the unadjusted GLMM will include the fixed categorical effects of intervention, time, and intervention-by-time interaction and the random-site effect. We will calculate the point estimates and their respective CIs for the changes in infants' weights for each intervention group and for the difference in the estimated change between intervention groups. Additionally, the team will present the p-value of the difference in point estimates between intervention groups.

The analysis team will examine the impact of the ESC care approach on length, head circumference, and infant weight for length (z-scores) using the same analytical methods described for weight (z-scores). Additionally, the team will provide the mean and SD of infant BMI-z at 24 months for each treatment group. The team will use a GLMM with an identity to compare average BMI-z between the groups, and the team will report point estimates for the group mean difference along with a 95% CI.

Sleep Assessed with the Brief Infant Sleep Questionnaire (BISQ)

The analysis team will provide the mean and SD of the BISQ survey separately for each treatment group. The team will use a generalized linear mixed model (GLMM) with appropriate link function (i.e., identity link for continuous outcome) to evaluate the effect of ESC on infant sleep duration. The model will examine how the treatment means differ (i.e., main treatment effect), how treatment means change over time (i.e., main time effect), and how differences between treatment means change over time (i.e., treatment-by-time effect). The team will carry out assessment at 3 months and 12 months of age. The GLMM analytical approach allows us to analyze correlated data obtained repeatedly from the same infant and account for the intracluster correlation coefficient among infants nested within with same clinical site. To account for potential imbalance in key demographic and site-level characteristics, the analysis team will utilize both unadjusted and adjusted GLMMs. Initially, the unadjusted GLMM will include the fixed categorical effects of intervention, time, and intervention-by-time interaction and the random-site effect. We will calculate the point estimates and their respective CIs for the changes in infants' BISQ scores for each intervention group and for the difference in the estimated change between intervention groups. Additionally, the team will present the p-value of the difference in point estimates between intervention groups.

Enteral Feeds during the First 6 Months of Life

We will measure enteral feeds on a nominal scale (i.e., exclusive maternal breastmilk, combination of maternal breastmilk and formula, or exclusive formula feeding). The analysis team will tabulate count and relative frequency for each level and for each treatment group. To evaluate the association between enteral feeds with intervention, the team will use a mixed-effects multinomial logistic regression model to account for the longitudinal cluster study design and potential participant and site-level covariates.

Breastfeeding during the First 6 Months of Life

The analysis team will report the proportion of direct breastfeeding for each treatment group during each of the assessment periods (1 month post-discharge, and 3 months and 6 months of age). To evaluate the association between breastfeeding with intervention, the team will use a mixed-effects logistic regression model to account for the longitudinal cluster study design and potential participant and site-level covariates. We will present odds ratio estimate of breastfeeding at each assessment period for the intervention effect (ESC versus usual institutional care) with 95% CI.

Number of Emergency Room Visits and/or Acute/Urgent Care Visits

The analysis team will examine the impact of the ESC care approach on the reduction of ER visits and/or acute/urgent care visits using the same analytical steps described for the primary outcome. Given that the outcome measure is count (number of ER visits, integers ≥ 1), we expect that Poisson regression analysis, adjusted for clustering at hospital will be appropriate. However, if the distribution should be approximate to normal or if the team observes over-dispersion, we will consider linear mixed-effect regression or negative binomial models. Again, the team will use the same four model building sequence described for the primary outcome. Specifically, we will start with an unadjusted model and conclude with a model that will include possible interaction between period and intervention effect.

Readmissions

The analysis team will compare the proportion readmissions between the treatment groups using a GLMM with a logistic link function. We will follow the same four modeling strategies described for the primary outcome, and we will present odds ratio estimate of readmissions for the intervention effect (ESC versus usual institutional care) with 95% CI.

5.1.4.2. Patient-Reported Outcomes Measurement Information System (PROMIS) Short Forms

The analysis team will measure primary caregiver(s)' well-being with PROMIS Short Forms. The team will convert raw scores to T-scores and report descriptive statistics (mean ± SD) for each of the five domains (i.e., emotional support, meaning and purpose, anger, anxiety, and depression) separately for each treatment group. To compare each domain composite scores between the ESC care approach and usual care, the team will use a GLMM model with identity link with a fixed effect for the intervention group, time, and group-by-time and a random effect for study site. Assessment periods will include at discharge, and at 6 months and 24 months. The team will report point estimates for the group mean difference along with a 95% CI. Again, this analytical approach will be repeated for each of the 5 PROMIS domains.

5.1.4.3. Maternal Postnatal Attachment Questionnaire

The analysis team will examine the impact of the ESC care approach on the composite score of the MPAQ and its three subscales (quality, absence of hostility towards infant, and pleasure). Since these measures are continuous, the team will apply the GLMM with the normal link function. In addition, the model will examine the ESC intervention impact at hospital discharge and 6 months of age.

5.1.4.4. Family Environment Scale (FES) at 3 Months

Initially, we will base the overall assessment of the FES using the relationship dimension on a composite score of the 30 true-false items found on form R (i.e., range of 0-30). The analysis team will provide the mean and SD of the composite FES scores separately for each treatment group, and the team will use a GLMM with an identity link function to compare average FES scores between the ESC care approach and usual care. We will report point estimates for the group mean difference along with a 95% CI. The team will use the same four model building sequence described for the primary outcome. Additionally, we will repeat the analytical for each relationship dimension, namely, Cohesion, Expressiveness, and Conflict subscales.

5.1.4.5. Parenting Sense of Competence Scale (PSOC)

The analysis team will report descriptive statistics (mean ± SD) for the composite PSOC score separately for each treatment group. The team will compare the PSOC composite scores assessed at hospital discharge and 6-months of age using a GLMM model with identity link with a fixed effect for the intervention group, time, and group-by-time and a random effect for study site. We will calculate the point estimates and their respective CIs for the changes in PSOC scores for each intervention group and for the difference in the estimated change between intervention groups. Additionally, the team will present the p-value of the difference in point estimates between intervention groups.

5.1.4.6. Infant Behavior Questionnaire (IBQ-R) Revised Very Short Form at 3 and 12 Months of Age

The analysis team will report descriptive statistics (mean ± SD) for each domain of the IBQ-R (i.e., positive affectivity/surgency, negative emotionality, and orienting/regulatory capacity) separately for each treatment group. The team will compare the IBQ-R composite scores for each domain using separate GLMM models with identity link with a fixed effect for the intervention group, time, and group-by-time and a random effect for study site. The team will report point estimates for the group mean difference along with a 95% CI for each domain.

5.1.4.7. Bayley Scales of Infant and Toddler Development, Fourth Edition (Bayley-4): Cognitive, Language, and Motor at 24-Months of Age

The analysis team will calculate descriptive statistics (mean ± SD, medians, percentiles) for each domain in the Bayley-4 separately for each treatment group. To compare the scores between the ESC and usual care groups, we will perform a linear mixed-effects model with a fixed effect for the intervention group and a random effect for study site. We will report point estimates for the group mean difference along with a 95% CI, and the team will repeat this analytical approach for each of the Bayley-4 domains.

5.1.4.8. Brief Infant-Toddler Social and Emotional Assessment (BITSEA) at 24-Months of Age

The analysis team will calculate descriptive statistics (mean ± SD, medians, percentiles) for BITSEA problem scale and BITSEA competence scale separately for each treatment group. To compare the

scores between the ESC and usual care groups among the two BITSEA scales, we will perform separate linear mixed-effects model with a fixed effect for the intervention group and a random effect for study site. We will report point estimates for the group mean difference along with a 95% CI.

5.1.4.9. Influence of Maternal Childhood Experiences on Infant Outcomes

The analysis team will calculate descriptive statistics (mean ± SD, medians, percentiles) for the ACE Questionnaire separately for each treatment group. To examine the relationship between the ACE Questionnaire with the IBQ-R scores and Bayley-4, the analysis team will compute separate marginal Pearson correlation coefficients,[63] which is an analog of the standard Pearson correlation coefficient for clustered data. If significant, we will perform a sensitivity analysis in which we include the ACE Questionnaire scores as a covariate in the final analytic models for Bayley-4 and IBQ-R scores.

5.1.4.10. Interim Analysis

In a stepped-wedge randomized controlled trial, interim analyses for outcomes carried out early in the trial will have a large imbalance between numbers of observations exposed to usual care and intervention conditions. The imbalance will likely have power implications and will make a power analysis infeasible. The clustered natures of the data will also impact the analysis.[64, 65] Therefore, the protocol study team will not conduct an interim analysis on the primary outcome for the purpose of study termination due to inferiority or superiority of the ESC care approach. The protocol study team will conduct an interim analysis for the long-term follow-up portion of the study to assess for futility due to under-recruitment. The projected informed consent rate for long-term follow-up is 30-40%. After each block of two periods (approximately 6 months), the protocol study team will compare the informed consent rate with the projected informed consent rate. If the actual informed consent rate over a block of two periods is below 30%, then the protocol study team will monitor the informed consent rate for another block of two periods. If the cumulative informed consent rate remains below 30%, then the protocol study team will ask the Data and Safety Monitoring Committee (DSMC) to review accrual trajectories and to determine, with the protocol study team, if measures can be taken to improve the accrual rate. The DSMC will consider whether to stop accrual to the long-term follow-up portion of the study due to an insufficient informed consent rate. Additionally, the DSMC will monitor the study for safety concerns (see Section 5.5.2).

5.2. SAMPLE SIZE AND POWER ESTIMATES

We based the sample size estimate (Table 5) on the primary outcome, which is the comparison of the average length of time until the infant is medically ready for discharge between groups (ESC care approach versus usual care). In much of the literature, researchers tend to report overall length of inpatient hospital stay (LOS). The average reported LOS for infants managed for NOWS is approximately 18 days (SD=8)[29]; we expect a reduction of 4 days with use of the ESC care approach. For this study, we used preliminary data from the ACT NOW Current Experience Study to obtain the mean and standard deviation estimate for LOS. For the purpose of our sample size justification, we used these estimates as a proxy for our estimates of the average length of time until the infant is medically ready for discharge. Based on the Current Experience Study, the average LOS is approximately 11 days (SD=11). Additionally, we derived an estimate of the ICC of 0.25 from this preliminary data analysis. Richard Hooper and colleagues[66] noted that most sample size justifications for stepped-wedge design studies follow a mixed-effects regression approach for cross-sectional stepped-wedge design, as described by Hussey and

Hughes,[67] which assumes that the within-period ICC and between-period ICC are equal. They define the cluster autocorrelation coefficient (CAC) as the ratio of the between-period ICC over the within-period ICC.

We calculated statistical power based on the methodology for stepped-wedge with transition period design proposed by Hooper et al using the R-Shiny app written by Hemming and Kasza.[66] Given that our primary outcome is a count measure, we used the ACT NOW Current Experience Study to obtain an estimate of the over-dispersion parameter (ϕ). McCullagh and Nelder suggested that the over-dispersion parameter estimate (ϕ) is simply a ratio of the deviance or the Pearson chi-square to its associated degrees-of-freedom.[68] Thus, a total sample size of 864 infants would achieve 90% power to detect a difference of 4 days between the groups with an estimated CAC of 0.8 and ϕ=10. This assumes an 8-step stepped-wedge with transition period design with 24 total sites. We will randomize each site into 1 of 8 blocks, and we expect each site to enroll an average of 4 infants during each period for 36 total infants per site during the study duration. Since we have no prior information regarding the CAC estimate, Table 5 provides the total sample size required assuming a CAC ranging between 0.6 to 0.8 and differences of 3 days, 3.5 days, and 4 days. Based on the ACT NOW Current Experience Study, the expected number of infants with NOWS delivered at participating sites annually will be approximately 1500-2000 infants. Therefore, our study will still be sufficiently powered (i.e., 85%) to detect a difference of 3 days between the groups with CAC=0.8. The power calculation assumes significance level of 5%, delivery of infants with NOWS equally distributed across hospital groupings, and analysis by Negative Binomial GLMM.

Table 5 Sample Size Estimates

		3 days		3.5 days		4 days	
Power	CAC	Cluster Size	N	Cluster Size	N	Cluster Size	N
**90%**	**0.8**	10	2160	6	1296	**4**	**864**
85%	0.8	8	1728	5	1080	3	648
80%	0.8	6	1296	4	864	3	648
90%	0.7	14	3024	7	1512	5	1080
85%	0.7	10	2160	6	1296	4	864
80%	0.7	8	1728	5	1080	3	648
90%	0.6	26	5616	9	1944	5	1080
85%	0.6	14	3024	6	1296	4	864
80%	0.6	10	2160	5	1080	3	648

CAC = cluster autocorrelation coefficient

To address the primary study hypothesis, the protocol study team will randomize a minimum of 24 sites, and a maximum of 28 sites to 1 of 8 blocks of a stepped wedge with transition design (Table 1), with each site enrolling an average of 36 infants. During any single study period (see Table 1), a site may enroll no more than 16 infants. Although we calculated the sample size for the overall trial using the power calculation for the primary hypothesis, we conducted the following power calculations to assure adequacy of sample size to show potential effect of the intervention on infant neurobehavioral functioning and development. To evaluate the impact of the ESC care approach on infant neurobehavioral function and development using measures such as the IBQ-R and Bayley-4, we must

obtain primary caregiver consent. We anticipate that not all participants will provide consent for the long-term outcome portion of the study. Table 6 provides an estimate of the effect size based on varying consent rates and CAC estimates with the study having 80% power. Again based on the ACT NOW Current Experience Study, we expect the number of infants with NOWS delivered at participating sites annually will be approximately 1500 infants. Thus, for a 17-month enrollment period, the protocol study team expects a total of 2125 infants with NOWS will be delivered at participating study sites. Assuming a 40% or 30% consent rate, this produces a total sample size of 850 (40% consent rate) or 638 (30% consent rate) infants. Cohen defined effect size as the mean differences, $\mu_1 - \mu_2$, divided by the standard deviation, σ, of either group.[69] However, Rosnow and Rosenthal noted that in practice, researchers commonly use the pooled SD (defined as the root mean square of the 2 SDs).[70] Effect sizes are generally classified as small (≤ 0.3), medium (~0.5), and large (≥ 0.75). For infant neurobehavioral functioning based on the IBQ-R, the study will have 80% power to detect an expected mean difference of 0.28 points in the Orienting/Regulatory Capacity domain, assuming a 30% consent rate and CAC=0.8, based on a mixed-effects model with a fixed treatment effect and random site effect with a significance level of 0.05. With a SD of 0.70, the detectable mean difference constitutes a moderate effect size. We based our estimated mean (5.0) and SD (0.70) for the Orienting/Regulatory Capacity domain using the summary statistics provided by Putnam and colleagues,[46] in which the authors provided summary data of the IBQ-R domains extracted from six standard form data samples.

Table 6 Sample Size Estimates for the IBQ-R for the Consented Subpopulation

Consent Rate	Total Sample Size	Effect Size (Δ)	Mean Difference	CAC	Power
40%	850	0.35	0.25	0.8	0.80
40%	850	0.38	0.27	0.7	0.80
40%	850	0.40	0.28	0.6	0.80
30%	638	0.40	0.28	0.8	0.80
30%	638	0.43	0.30	0.7	0.80
30%	638	0.45	0.32	0.6	0.80

CAC = cluster autocorrelation coefficient

For the neurodevelopmental outcome based on the Bayley-4, the study will have 80% power to detect an expected mean difference of 6 points, assuming a 30% consent rate and CAC=0.8, based on a mixed-effects model with a fixed treatment effect and random site effect with a significance level of 0.05. With a SD of 15, the detectable mean difference constitutes a moderate effect size.

5.3. AVAILABLE POPULATION

In December 2018, we completed data abstraction for the ACT NOW Current Experience Study. Twenty-five ISPCTN and 5 NRN sites participated in this study. We collected data for 1808 infants with opioid exposure across these networks. Of these infants, approximately 40% were treated pharmacologically during the 1-year target period of July 1, 2016, to June 30, 2017 (86% morphine, 13% methadone, <1% buprenorphine).

5.4. PROJECTED RECRUITMENT TIME

5.4.1. Site Recruitment

We will recruit 24 sites for this study. We will randomize these sites into 8 blocks. Initial assessment of site interest in study participation across the networks suggests an adequate number of sites to meet our site recruitment goal. The site's ability to initiate a change in practice within their organization will impact actual site recruitment. Recruitment of all sites will take an estimated 3 months. We will randomize sites into blocks once recruitment is complete.

5.4.2. Site Training and Implementation

Site training and implementation will take approximately 3 months, as we will first train a core group of site champions, followed by training of all site personnel by the core group. The protocol study team will train the site champions via video conference or off-site and as such, training may occur in parallel with the end of the final usual care period at the site. Once a site has achieved the training milestones (see Training and Implementation Manual), the site will clinically implement ESC. After this initial implementation, the site will step into the ESC period (see Table 1). Total enrollment period is 20 months with each site actively enrolling infants for 17 of the 20 months. If the site research team obtains consent for the long-term follow-up portion of the trial, the site research team will follow the infant for 24 months. Total length of the study will be approximately 44 months.

5.5. STUDY MONITORING PLAN

We will conduct clinical site monitoring to ensure that we protect the rights and well-being of study participants, that the reported trial data are accurate, complete, and verifiable, and that the conduct of the study complies with the currently approved protocol/amendment(s), with International Council for Harmonisation Good Clinical Practice, and with applicable regulatory requirements (for details specific to Protocol Adherence see Section 4.2.5)

- A member of the DCC clinical operations staff or their designee will monitor the study.
- The clinical monitoring team will plan and conduct an on-site visit at least once during the course of the study and more often if needed for cause.
- Details of clinical site monitoring are in the Clinical Monitoring Plan, which will be included in the MOP. The plan describes who will conduct the monitoring, at what frequency monitoring will occur, at what level of detail monitoring will be performed, and how monitoring reports will be distributed.

5.5.1. Adverse Events

5.5.1.1. <u>Definition of Adverse Events and Serious Adverse Events</u>

Adverse event (AE): AE means any untoward medical occurrence associated with the use of an intervention in humans, whether or not considered intervention related.

Serious Adverse Event (SAE): An AE is considered "serious" if, in the view of either the investigator or sponsor, it results in any of the following outcomes:

1. Death

2. Life-threatening AE (life-threatening means that the study participant was, in the opinion of the investigator or sponsor, at immediate risk of death from the reaction as it occurred and required intervention)
3. Persistent or significant incapacity or substantial disruption of the ability to conduct normal life functions
4. Inpatient hospitalization or prolongation of existing hospitalization
5. Important medical event that may not result in 1 of the above outcomes but may jeopardize the health of the study participant or require medical or surgical intervention to prevent 1 of the outcomes listed in the above definition of serious event

5.5.1.2. Classification of an Adverse Event

Severity of Event

For AEs, the site research team will use the following guidelines to describe severity. The site investigator will determine severity.

- **Mild** – Events require minimal or no treatment and do not interfere with the participant's daily activities.
- **Moderate** – Events result in a low level of inconvenience or concern with the therapeutic measures. Moderate events may cause some interference with functioning.
- **Severe** – Events interrupt a participant's usual daily activity and may require systemic drug therapy or other treatment. Severe events are usually potentially life threatening or incapacitating. Of note, the term "severe" does not necessarily equate to "serious."

Relationship to Study Intervention

The site research team will grade the degree of certainty about causality by using the categories below.

- **Related** – The AE is known to occur with the study procedures, there is a reasonable possibility that the study procedures caused the AE, or there is a temporal relationship between the study procedures and the event. Reasonable possibility means that there is evidence to suggest a causal relationship between the study procedures and the AE.
- **Not Related** – There is not a reasonable possibility that the study procedures caused the event, there is no temporal relationship between the study procedures and event onset, or an alternate etiology has been established.

Expected AEs

Expected AEs include - seizures, accidental trauma, severe weight loss (greater than 15% from birthweight) and respiratory insufficiency. Expected AEs that could occur during the follow-up portion of the study include acute/urgent care and or ER visits for worsening symptoms of NOWS. Hospital readmission to assess and manage symptoms of NOWS and non-accidental trauma may also occur. We note anticipated rates in Table 7.

5.5.1.3. Time Period and Frequency for Event Assessment and Follow Up

For this study, the protocol study team will collect the following AEs: 1) all expected AEs (seizures, accidental trauma, severe weight loss, and respiratory insufficiency), and 2) SAEs related to study procedures. The occurrence of an AE or SAE may come to the attention of study personnel during the hospital stay, by the clinical team with administration of questionnaires, or by the medical monitor upon reviewing data. The site research team will capture all AEs on the appropriate case report form. Information to be collected includes event description, date/time of onset, date/time of resolution, clinician's assessment of severity, relationship to study intervention and time of resolution/stabilization of the event. Site research teams must follow all AEs until the AE meets one of the following criteria: resolution, the condition stabilizes, the event is otherwise explained or is judged by the protocol study team to be no longer clinically significant, or the participant is lost to follow-up. The site research team will collect AEs during the initial hospitalization through hospital discharge.

5.5.2. Data Monitoring and Safety

The independent DSMC will have overall responsibility for interim data monitoring and operate based on the ISPCTN and NRN charter for the DSMC. The DSMC will formally review interim safety data in a sequential fashion using interim monitoring boundaries after approximately 25%, 50%, and 75% of the study sites (6, 12, and 18 sites, respectively) have transitioned to the ESC care approach and have completed one full period using the ESC care approach. Treatment groups will be compared statistically using the analysis planned for the final analyses for safety outcomes (as specified in Section 5.1.3).

Safety oversight will be under the direction of a DSMC. Safety outcomes include the components of the inpatient composite safety outcome and those of the outpatient composite safety outcome (see Table 7). The DSMC may request other outcomes at their discretion. Formal statistical testing for an imbalance of seizures, accidental trauma, or respiratory insufficiency due to opioid therapy, in either treatment group, will be based on a comparatively liberal Lan DeMets Pocock boundary at the three interim safety reviews to guard against any occurrence of false positives while at the same time allowing for stopping at reasonable levels of evidence. Thus, at each interim, an increased incidence of seizures in either treatment group with $P < 0.0179$ (for 4 total tests) will be considered a statistically significant evidence of harm that the DSMC can use to recommend suspension of the trial for safety reasons. This same statistical testing will also be conducted for the components of the outpatient composite safety outcome. In addition to the formal safety outcomes, the DSMC will examine other safety outcomes, including all reported SAEs by treatment group in considering a recommendation to suspend the trial for safety reasons.

The Medical Monitor will provide input on safety considerations, evaluate safety trends, and provide oversight throughout the life cycle of the clinical research, in accordance with the approved protocol. This role includes reviewing and monitoring safety events on a regular basis, advising the protocol investigators on trial-related medical questions or problems, reviewing cumulative participant safety data, and making recommendations regarding the data to the DSMC.

Table 7 Expected Rates of Safety Outcomes

**Safety Outcomes**	**Expected Rate**
**Inpatient Safety Composite**	
Seizures[71, 72]	1%
Accidental trauma (i.e., dropped infants)[73, 74]	4 falls per 10,000 births
Respiratory insufficiency due to opioid therapy	0.5%
**Outpatient Safety Composite**	
Acute/urgent care and/or ER visits – 1 visit in first 6 months of life[75]	35%
Hospital Readmissions in the first 6 months of life[75]	7%

SECTION 6. DATA MANAGEMENT

The data management center, RTI International, will:

- Collaborate in the development, implementation, and monitoring of ESC protocol.
- Provide data management, including development of CRFs and appropriate data collection systems
- Supervise data entry activities, including instructing and certifying data entry personnel in software and hardware usage, quality assurance of data entry, etc.
- Manage the Data Safety and Monitoring Committee for the trial. This will include scheduling meetings and the DSMC charter.
- Oversee the receipt and reconciliation of safety data.
- Supervise NRN-site quality assurance efforts, including conducting site visits and remote monitoring of data.
- Prepare and distributes monthly reports, detailing data received, data consistency, missing data and adherence to protocol.
- Disburse capitation payments to clinical centers on the basis of enrolled participants and other study-specific milestone triggers specified in the study protocol.
- Provide the logistical support necessary to run an efficient and productive network.

SECTION 7. PUBLICATION AND DATA SHARING POLICY

This study will comply with the National Institutes of Health (NIH) Public Access Policy, which ensures that the public has access to the published results of NIH-funded research. The study will also comply with the NIH Data Sharing Policy and Policy on the Dissemination of NIH-Funded Clinical Trial Information and the Clinical Trials Registration and Results Information Submission rule.

As such, this study will:

- Register with ClinicalTrials.gov and submit primary outcome results. The ClinicalTrial.gov number is NCT04057820.
- Publish results. The protocol study team will make every attempt to publish results in peer-reviewed journals. The team will submit all final peer-reviewed journal manuscripts from this study to the digital archive PubMed Central upon acceptance for publication.
- Deposit data for data sharing with other researchers. Within the bounds of relevant IRB approvals and guidelines for protection of personally identifiable data, the protocol study team will deposit de-identified data from this study in an appropriate, NIH-approved data repository.

SECTION 8. REFERENCES

1. Brothers KB, Glascoe FP, Robertshaw NS. PEDS: developmental milestones--an accurate brief tool for surveillance and screening. *Clin Pediatr (Phila)* 2008, **47**(3)**:** 271-279.

2. Patrick SW, Davis MM, Lehman CU, Cooper WO. Increasing incidence and geographic distribution of neonatal abstinence syndrome: United States 2009 to 2012. *J Perinatol* 2015, **35**(8)**:** 667.

3. Patrick SW, Schumacher RE, Benneyworth BD, Krans EE, McAllister JM, Davis MM. Neonatal abstinence syndrome and associated health care expenditures: United States, 2000-2009. *JAMA* 2012, **307**(18)**:** 1934-1940.

4. Tolia VN, Patrick SW, Bennett MM, Murthy K, Sousa J, Smith PB*, et al.* Increasing incidence of the neonatal abstinence syndrome in U.S. neonatal ICUs. *The New England journal of medicine* 2015, **372**(22)**:** 2118-2126.

5. Hudak ML, Tan RC, Committee On D, Committee On F, Newborn, American Academy of P. Neonatal drug withdrawal. *Pediatrics* 2012, **129**(2)**:** e540-560.

6. Reddy UM, Davis JM, Ren Z, Greene MF, Opioid Use in Pregnancy NAS, Childhood Outcomes Workshop Invited S. Opioid Use in Pregnancy, Neonatal Abstinence Syndrome, and Childhood Outcomes: Executive Summary of a Joint Workshop by the Eunice Kennedy Shriver National Institute of Child Health and Human Development, American College of Obstetricians and Gynecologists, American Academy of Pediatrics, Society for Maternal-Fetal Medicine, Centers for Disease Control and Prevention, and the March of Dimes Foundation. *Obstet Gynecol* 2017, **130**(1)**:** 10-28.

7. Office USGA. Newborn Health: Federal Action Needed to Address Neonatal Abstinence Syndrome. Washington, D.C.; 2017. Report No.: GAO-18-32.

8. Centers for Disease Control and Prevention. Vital signs: prescription painkiller overdoses: a growing epidemic, especially among women. 2016 [cited 09/25/17]Available from: http://www.cdc.gov/vitalsigns/prescriptionpainkilleroverdoses/

9. International Narcotics Control Board. Report of the International Narcotics Control Board for 2010. 2010 [cited 09/25/17]Available from: https://www.incb.org/documents/Publications/AnnualReports/AR2010/AR_2010_English.pdf

10. Ailes EC, Dawson AL, Lind JN, Gilboa SM, Frey MT, Broussard CS*, et al.* Opioid prescription claims among women of reproductive age--United States, 2008-2012. *MMWR Morb Mortal Wkly Rep* 2015, **64**(2)**:** 37-41.

11. O'Donnell JK, Gladden RM, Seth P. Trends in Deaths Involving Heroin and Synthetic Opioids Excluding Methadone, and Law Enforcement Drug Product Reports, by Census Region - United States, 2006-2015. *MMWR Morb Mortal Wkly Rep* 2017, **66**(34)**:** 897-903.

12. Haight SC, Ko JY, Tong VT, Bohm MK, Callaghan WM. Opioid Use Disorder Documented at Delivery Hospitalization - United States, 1999-2014. *MMWR Morb Mortal Wkly Rep* 2018, **67**(31)**:** 845-849.

13. Quality AfHCRa. Health Care Cost and Utilization Project (HCUP). . Rockville. MD; 2018.

14. Winkelman TNA, Villapiano N, Kozhimannil KB, Davis MM, Patrick SW. Incidence and Costs of Neonatal Abstinence Syndrome Among Infants With Medicaid: 2004-2014. *Pediatrics* 2018, **141**(4).

15. Milliren CE, Gupta M, Graham DA, Melvin P, Jorina M, Ozonoff A. Hospital Variation in Neonatal Abstinence Syndrome Incidence, Treatment Modalities, Resource Use, and Costs Across Pediatric Hospitals in the United States, 2013 to 2016. *Hosp Pediatr* 2018, **8**(1)**:** 15-20.

16. Patrick SW, Faherty LJ, Dick AW, Scott TA, Dudley J, Stein BD. Association Among County-Level Economic Factors, Clinician Supply, Metropolitan or Rural Location, and Neonatal Abstinence Syndrome. *JAMA* 2019, **321**(4)**:** 385-393.

17. Villapiano NL, Winkelman TN, Kozhimannil KB, Davis MM, Patrick SW. Rural and Urban Differences in Neonatal Abstinence Syndrome and Maternal Opioid Use, 2004 to 2013. *JAMA Pediatr* 2017, **171**(2)**:** 194-196.

18. Kraft WK, Stover MW, Davis JM. Neonatal abstinence syndrome: Pharmacologic strategies for the mother and infant. *Semin Perinatol* 2016, **40**(3)**:** 203-212.

19. Gomez Pomar E, Finnegan LP, Devlin L, Bada H, Concina VA, Ibonia KT*, et al.* Simplification of the Finnegan Neonatal Abstinence Scoring System: retrospective study of two institutions in the USA. *BMJ Open* 2017, **7**(9)**:** e016176.

20. Jones HE, Seashore C, Johnson E, Horton E, O'Grady KE, Andringa K*, et al.* Psychometric assessment of the Neonatal Abstinence Scoring System and the MOTHER NAS Scale. *Am J Addict* 2016, **25**(5)**:** 370-373.

21. Mehta A, Forbes KD, Kuppala VS. Neonatal Abstinence Syndrome Management From Prenatal Counseling to Postdischarge Follow-up Care: Results of a National Survey. *Hosp Pediatr* 2013, **3**(4)**:** 317-323.

22. Zimmermann-Baer U, Notzli U, Rentsch K, Bucher HU. Finnegan neonatal abstinence scoring system: normal values for first 3 days and weeks 5-6 in non-addicted infants. *Addiction* 2010, **105**(3)**:** 524-528.

23. Maguire D, Cline GJ, Parnell L, Tai CY. Validation of the Finnegan neonatal abstinence syndrome tool-short form. *Adv Neonatal Care* 2013, **13**(6)**:** 430-437.

24. Jansson LM, Velez M, Harrow C. The opioid-exposed newborn: assessment and pharmacologic management. *J Opioid Manag* 2009, **5**(1)**:** 47-55.

25. Timpson W, Killoran C, Maranda L, Picarillo A, Bloch-Salisbury E. A Quality Improvement Initiative to Increase Scoring Consistency and Accuracy of the Finnegan Tool: Challenges in Obtaining Reliable Assessments of Drug Withdrawal in Neonatal Abstinence Syndrome. *Adv Neonatal Care* 2018, **18**(1)**:** 70-78.

26. Grossman MR, Berkwitt AK, Osborn RR, Xu Y, Esserman DA, Shapiro ED*, et al.* An Initiative to Improve the Quality of Care of Infants With Neonatal Abstinence Syndrome. *Pediatrics* 2017, **139**(6).

27. Grossman MR, Lipshaw MJ, Osborn RR, Berkwitt AK. A Novel Approach to Assessing Infants With Neonatal Abstinence Syndrome. *Hosp Pediatr* 2018, **8**(1)**:** 1-6.

28. Whalen BL MK, Grossman MR, Whatley C, Flanagan V, Minear S, Tosteson T, Verma K, Harris K, Loring C, Zhao W, Wachman EM. . Inter- and Intra-Rater Reliability of the Eating, Sleeping, Consoling (ESC) Care Tool for Neonatal Abstinence Syndrome (NAS). 2018.

29. Wachman EM, Grossman M, Schiff DM, Philipp BL, Minear S, Hutton E*, et al.* Quality improvement initiative to improve inpatient outcomes for Neonatal Abstinence Syndrome. *J Perinatol* 2018.

30. Wachman EM, Schiff DM, Silverstein M. Neonatal Abstinence Syndrome: Advances in Diagnosis and Treatment. *JAMA* 2018, **319**(13)**:** 1362-1374.

31. Holmes AV, Atwood EC, Whalen B, Beliveau J, Jarvis JD, Matulis JC*, et al.* Rooming-In to Treat Neonatal Abstinence Syndrome: Improved Family-Centered Care at Lower Cost. *Pediatrics* 2016, **137**(6).

32. McKnight S, Coo H, Davies G, Holmes B, Newman A, Newton L*, et al.* Rooming-in for Infants at Risk of Neonatal Abstinence Syndrome. *Am J Perinatol* 2016, **33**(5)**:** 495-501.

33. Ko JY, Patrick SW, Tong VT, Patel R, Lind JN, Barfield WD. Incidence of Neonatal Abstinence Syndrome - 28 States, 1999-2013. *MMWR Morb Mortal Wkly Rep* 2016, **65**(31)**:** 799-802.

34. MacMillan KDL. Understanding Family Perspectives: A Follow-Up Qualitative Study of Family Experience with Hospitalization for Neonatal Abstinence Syndrome. Dartmouth School of Medicine; 2018.

35. McRae AD, Weijer C, Binik A, Grimshaw JM, Boruch R, Brehaut JC*, et al.* When is informed consent required in cluster randomized trials in health research? *Trials* 2011, **12:** 202.

36. Brown MS, Hayes MJ, Thornton LM. Methadone versus morphine for treatment of neonatal abstinence syndrome: a prospective randomized clinical trial. *J Perinatol* 2015, **35**(4)**:** 278-283.

37. Davis JM, Shenberger J, Terrin N, Breeze JL, Hudak M, Wachman EM*, et al.* Comparison of Safety and Efficacy of Methadone vs Morphine for Treatment of Neonatal Abstinence Syndrome: A Randomized Clinical Trial. *JAMA Pediatr* 2018.

38. Kraft WK, Adeniyi-Jones SC, Chervoneva I, Greenspan JS, Abatemarco D, Kaltenbach K*, et al.* Buprenorphine for the Treatment of the Neonatal Abstinence Syndrome. *The New England journal of medicine* 2017, **376**(24)**:** 2341-2348.

39. Abdel-Latif ME, Pinner J, Clews S, Cooke F, Lui K, Oei J. Effects of breast milk on the severity and outcome of neonatal abstinence syndrome among infants of drug-dependent mothers. *Pediatrics* 2006, **117**(6)**:** e1163-1169.

40. McQueen KA, Murphy-Oikonen J, Gerlach K, Montelpare W. The impact of infant feeding method on neonatal abstinence scores of methadone-exposed infants. *Adv Neonatal Care* 2011, **11**(4)**:** 282-290.

41. Gartstein MAR, M.K. . Studying infant temperament via the Revised Infant Behavior Questionnaire. *Infant Behavior & Development* 2003, **26:** 64–86.

42. Bosquet Enlow M, White MT, Hails K, Cabrera I, Wright RJ. The Infant Behavior Questionnaire-Revised: Factor structure in a culturally and sociodemographically diverse sample in the United States. *Infant Behav Dev* 2016, **43:** 24-35.

43. Gartstein MA, Marmion J. Fear and positive affectivity in infancy: convergence/discrepancy between parent-report and laboratory-based indicators. *Infant Behav Dev* 2008, **31**(2)**:** 227-238.

44. Goldsmith HH, Campos JJ. The structure of temperamental fear and pleasure in infants: a psychometric perspective. *Child Dev* 1990, **61**(6)**:** 1944-1964.

45. Parade SH, Leerkes EM. The reliability and validity of the Infant Behavior Questionnaire-Revised. *Infant Behav Dev* 2008, **31**(4)**:** 637-646.

46. Putnam SP, Helbig AL, Gartstein MA, Rothbart MK, Leerkes E. Development and assessment of short and very short forms of the infant behavior questionnaire-revised. *J Pers Assess* 2014, **96**(4)**:** 445-458.

47. Sadeh A. A brief screening questionnaire for infant sleep problems: validation and findings for an Internet sample. *Pediatrics* 2004, **113**(6)**:** e570-577.

48. Cella D, Riley W, Stone A, Rothrock N, Reeve B, Yount S*, et al.* The Patient-Reported Outcomes Measurement Information System (PROMIS) developed and tested its first wave of adult self-reported health outcome item banks: 2005-2008. *J Clin Epidemiol* 2010, **63**(11)**:** 1179-1194.

49. Thomas KA, Spieker S. Sleep, Depression, and Fatigue in Late Postpartum. *MCN Am J Matern Child Nurs* 2016, **41**(2)**:** 104-109.

50. Busse M, Stromgren K, Thorngate L, Thomas KA. Parents' responses to stress in the neonatal intensive care unit. *Crit Care Nurse* 2013, **33**(4)**:** 52-59; quiz 60.

51. Condon JT, Corkindale CJ. The assessment of parent-to-infant attachment: Development of a self-report questionnaire instrument. *Journal of Reproductive and Infant Psychology* 1998, **16**(1)**:** 57-76.

52. Rossen L, Hutchinson D, Wilson J, Burns L, C AO, Allsop S*, et al.* Predictors of postnatal mother-infant bonding: the role of antenatal bonding, maternal substance use and mental health. *Arch Womens Ment Health* 2016, **19**(4)**:** 609-622.

53. Codon JTC, C.J. The assessment of parent-to-infant attachement: Development of a self-report questionnaire instrument. *Journal of Reproductive and Infant Psychology* 1998, **16**(1)**:** 57-76.

54. Brown S, Hicks LM, Tracy EM. Parenting Efficacy and Support in Mothers With Dual Disorders in a Substance Abuse Treatment Program. *J Dual Diagn* 2016, **12**(3-4)**:** 227-237.

55. Moos RHM, B.S. *Family Environmental Scale Manual*. Consulting Psychologists Press, INC: Palo Alto California, 1981.

56. Felitti VJ, Anda RF, Nordenberg D, Williamson DF, Spitz AM, Edwards V*, et al.* Relationship of childhood abuse and household dysfunction to many of the leading causes of death in adults. The Adverse Childhood Experiences (ACE) study. *Am J Prev Med* 1998, **14**(4)**:** 245-258.

57. Albers CA, Grieve AJ. Review of Bayley Scales of Infant and Toddler Development--Third Edition. *Journal of Psychoeducational Assessment* 2007, **25**(2)**:** 180-190.

58. Briggs-Gowan MJ, Carter AS, Irwin JR, Wachtel K, Cicchetti DV. The Brief Infant-Toddler Social and Emotional Assessment: screening for social-emotional problems and delays in competence. *J Pediatr Psychol* 2004, **29**(2)**:** 143-155.

59. Gibbons LE, Feldman BJ, Crane HM, Mugavero M, Willig JH, Patrick D*, et al.* Migrating from a legacy fixed-format measure to CAT administration: calibrating the PHQ-9 to the PROMIS depression measures. *Qual Life Res* 2011, **20**(9)**:** 1349-1357.

60. Pilkonis PA, Yu L, Dodds NE, Johnston KL, Maihoefer CC, Lawrence SM. Validation of the depression item bank from the Patient-Reported Outcomes Measurement Information System (PROMIS) in a three-month observational study. *J Psychiatr Res* 2014, **56:** 112-119.

61. Xie T, Waksman J. Design and sample size estimation in clinical trials with clustered survival times as the primary endpoint. *Stat Med* 2003, **22**(18)**:** 2835-2846.

62. Rosner B, Glynn RJ, Lee ML. Incorporation of clustering effects for the Wilcoxon rank sum test: a large-sample approach. *Biometrics* 2003, **59**(4)**:** 1089-1098.

63. Lorenz DJ, Datta S, Harkema SJ. Marginal association measures for clustered data. *Stat Med* 2011, **30**(27)**:** 3181-3191.

64. Grayling MJ, Wason JM, Mander AP. Stepped wedge cluster randomized controlled trial designs: a review of reporting quality and design features. *Trials* 2017, **18**(1)**:** 33.

65. Hemming K, Taljaard M, McKenzie JE, Hooper R, Copas A, Thompson JA*, et al.* Reporting of stepped wedge cluster randomised trials: extension of the CONSORT 2010 statement with explanation and elaboration. *BMJ* 2018, **363:** k1614.

66. Hooper R, Teerenstra S, de Hoop E, Eldridge S. Sample size calculation for stepped wedge and other longitudinal cluster randomised trials. *Stat Med* 2016, **35**(26)**:** 4718-4728.

67. Hussey MA, Hughes JP. Design and analysis of stepped wedge cluster randomized trials. *Contemp Clin Trials* 2007, **28**(2)**:** 182-191.

68. McCullagh P, Nelder, JA *Generalized Linear Models, Second Edition*. Chapman and Hall, 1989.

69. Cohen J. *Statistical power analysis for the behavioral sciences 2nd ed.* Erlbaum: Hillsdale NJ, 1988.

70. Rosnow RLR, R. . Computing contrasts, effect sizes, and counternulls on other people's published data: General procedures for research consumers. *Psychological Methods* 1996, **1**(4)**:** 331-340.

71. Osborn DA, Jeffery HE, Cole MJ. Opiate treatment for opiate withdrawal in newborn infants. *The Cochrane database of systematic reviews* 2010(10)**:** Cd002059.

72. Gomez-Pomar E. Simplification of the Finnegan Neonatal Abstinence Scoring System. Retrospective Study of Two Institutions in the US. *BMJ Open*, **(in press).**

73. Merhar SL, Kline-Fath BM, Nathan AT, Melton KR, Bierbrauer KS. Identification and management of neonatal skull fractures. *J Perinatol* 2016, **36**(8)**:** 640-642.

74. Loyal J, Pettker CM, Raab CA, O'Mara E, Lipkind HS. Newborn Falls in a Large Tertiary Academic Center Over 13 Years. *Hosp Pediatr* 2018, **8**(9)**:** 509-514.

75. Maalouf FI, Cooper WO, Slaughter JC, Dudley J, Patrick SW. Outpatient Pharmacotherapy for Neonatal Abstinence Syndrome. *J Pediatr* 2018, **199:** 151-157 e151.
