# Supplementary material for: Influence of Eat, Sleep, and Console on Infants Pharmacologically Treated for Opioid Withdrawal: A Post Hoc Subgroup Analysis of the ESC-NOW Randomized Clinical Trial
Source: JAMA Pediatr. 2024 Apr 15;178(6):525–32. doi: 10.1001/jamapediatrics.2024.0544 (PMC11019446; doi:10.1001/jamapediatrics.2024.0544)
Supplement: Supplement 2. — eMethods 1. Heterogeneity of intervention effect across individual sites eResults eTable 1. Length of hospital stay: number of infants in each site by intervention groups eFigure 1. Length of hospital stay: unadjusted marginal mean difference of hospital length of stay between usual care and ESC across sites eTable 2. Receipt of adjuvant therapy: number of infants in each site by intervention group eFigure 2. Receipt of adjuvant therapy: differences in unadjusted average probability of receiving adjuvant therapy between usual care and ESC across sites eMethods 2. Statistical analysis outline [file jamapediatr-e240544-s002.pdf]

Supplemental Online Content

Devlin LA, Hu Z, Merhar SL, et al; Eunice Kennedy Shriver NICHD Neonatal Research Network and NIH Environmental Influences on Child Health Outcomes (ECHO) Program Institutional Development Award States Pediatric Clinical Trials Network. Influence of eat, sleep, and console on infants pharmacologically treated for opioid withdrawal: a post hoc subgroup analysis of the ESC-NOW randomized clinical trial. *JAMA Pediatr*. Published online April 15, 2024. doi:10.1001/jamapediatrics.2024.0544

eMethods 1. Heterogeneity of intervention effect across individual sites
eResults
eTable 1. Length of hospital stay: number of infants in each site by intervention groups
eFigure 1. Length of hospital stay: unadjusted marginal mean difference of hospital length of stay between usual care and ESC across sites
eTable 2. Receipt of adjuvant therapy: number of infants in each site by intervention group
eFigure 2. Receipt of adjuvant therapy: differences in unadjusted average probability of receiving adjuvant therapy between usual care and ESC across sites
eMethods 2. Statistical analysis outline

This supplemental material has been provided by the authors to give readers additional information about their work.

eMethods 1. Heterogeneity of intervention effect across individual sites

We conducted sensitivity analyses to assess the robustness of the main findings. We assessed whether the effect of ESC was constant across the 26 study sites. In general, the results of the analyses support the robustness of the findings across site.

To assess for heterogeneity of treatment effect across individual sites, we tested for interactions between treatment and the site fixed effect. We included a variable for site and interaction variable between intervention and site into the regression models, adjusted for the same variables listed in Table 1 and the variable strata that was used for randomization. For those outcomes that having significant interaction effects between intervention and site, we fit the regression models stratified by sites, then plotted the treatment effect at individual sites to allow for visual comparison. Due to small sample size at certain sites, we fitted regression model stratified by sites with more than 30 total infants or more than 5 infants per intervention group. For the remaining sites with small sample sizes, we combined them to create a stratum. It is important to note that these sensitivity analyses are only intended to identify whether heterogeneity in intervention effect exists. These data, which are limited by sample size and distribution of the number of infants in each intervention group, are not intended to allow for site specific inferences to be made.

eResults

After adjusting for period, site strata, race, adequate prenatal care, MOUD, polysubstance exposure, RUCA, sex, gestational age, inborn, postnatal opioid type, the interaction between the treatment and site fixed effect are not statistically significant for the outcomes: time until opioid treatment initiated, peak opioid dose, total number of opioid doses, total postnatal

© 2024 Devlin LA et al. *JAMA Pediatrics.*

opioid treatment, and length of opioid treatment. For the outcomes: receipt of adjuvant therapy and length of hospital stay, that has significant interaction effect between intervention and sites, the results of stratified analysis by site are shown in eFigure 1 and eFigure 2.

© 2024 Devlin LA et al. *JAMA Pediatrics.*

eTable 1. Length of Hospital Stay: Number of infants in each site by intervention groups

Site	Usual Care N	ESC N	Total N
1	0	2	2
3*	6	10	16
4*	6	24	30
5*	6	21	27
6	1	1	2
7	0	2	2
8	9	5	14
9	2	0	2
10	15	3	18
11	7	3	10
12	2	1	3
13	8	3	11
14*	15	8	23
15*	27	14	41
16*	42	25	67
17	13	3	16
18	9	2	11
19	13	1	14
20	4	0	4
21	8	0	8
22	18	2	20
23*	38	6	44
24*	52	6	58
25	4	0	4
26	15	1	16
Total	320	143	463

*Sites with more than 30 infants or more than 5 infants per intervention group, to run regression models within the individual site

© 2024 Devlin LA et al. *JAMA Pediatrics.*

eFigure 1. Length of Hospital Stay: Unadjusted Marginal Mean Difference of Hospital Length of Stay between Usual Care and ESC Across Sites

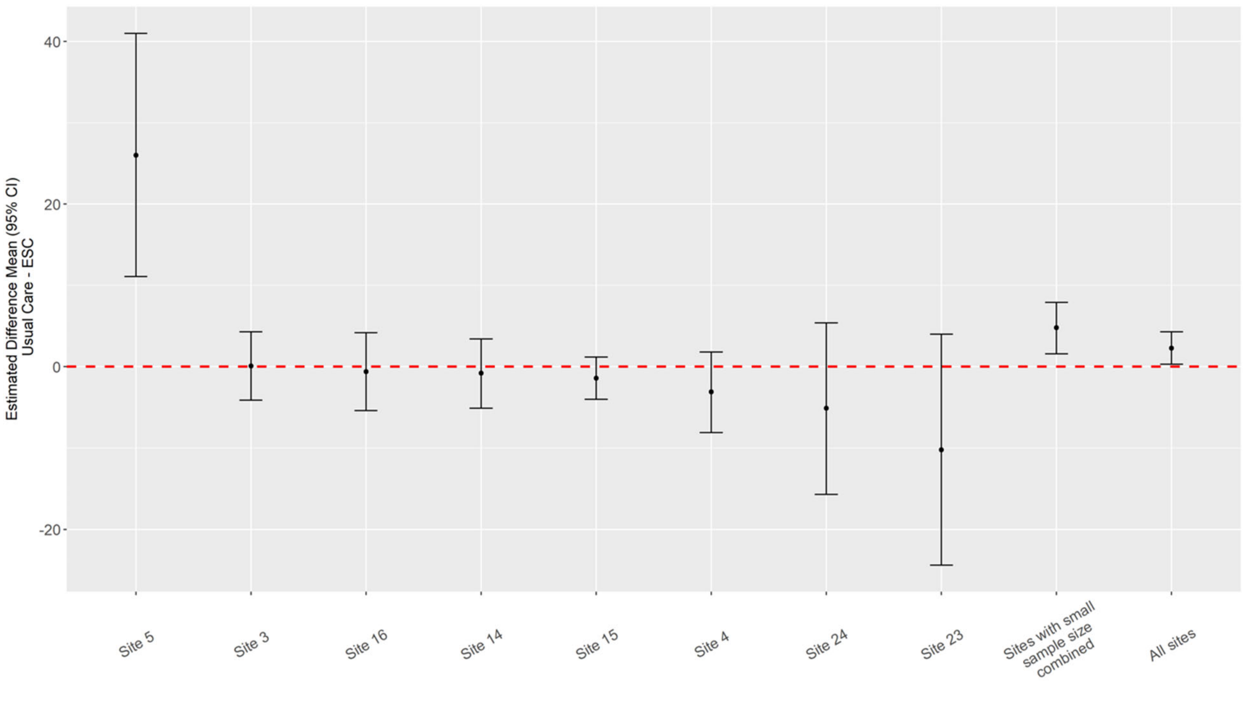

© 2024 Devlin LA et al. *JAMA Pediatrics.*

eTable 2. Receipt of Adjuvant Therapy: Number of Infants in Each Site by Intervention Group

Site	Usual Care N	ESC N	Total N
1	0	2	2
3**	6	10	16
4*	6	24	30
5*	6	21	27
6	1	1	2
7	0	2	2
8	9	5	14
9	2	0	2
10	15	3	18
11	7	3	10
12	2	1	3
13	8	3	11
14**	15	8	23
15*	27	14	41
16*	42	25	67
17	13	3	16
18	9	2	11
19	13	1	14
20	4	0	4
21	8	0	8
22	18	2	20
23*	38	6	44
24*	52	6	58
25	4	0	4
26	15	1	16
Total	320	143	463

*Sites with more than 30 infants or more than 5 infants per intervention group, to run regression models within the individual site
**Sites were excluded from the analysis due to no use of adjuvant therapy

© 2024 Devlin LA et al. *JAMA Pediatrics.*

eFigure 2. Receipt of Adjuvant Therapy: Differences in Unadjusted Average Probability of Receiving Adjuvant Therapy Between Usual Care and ESC Across Sites

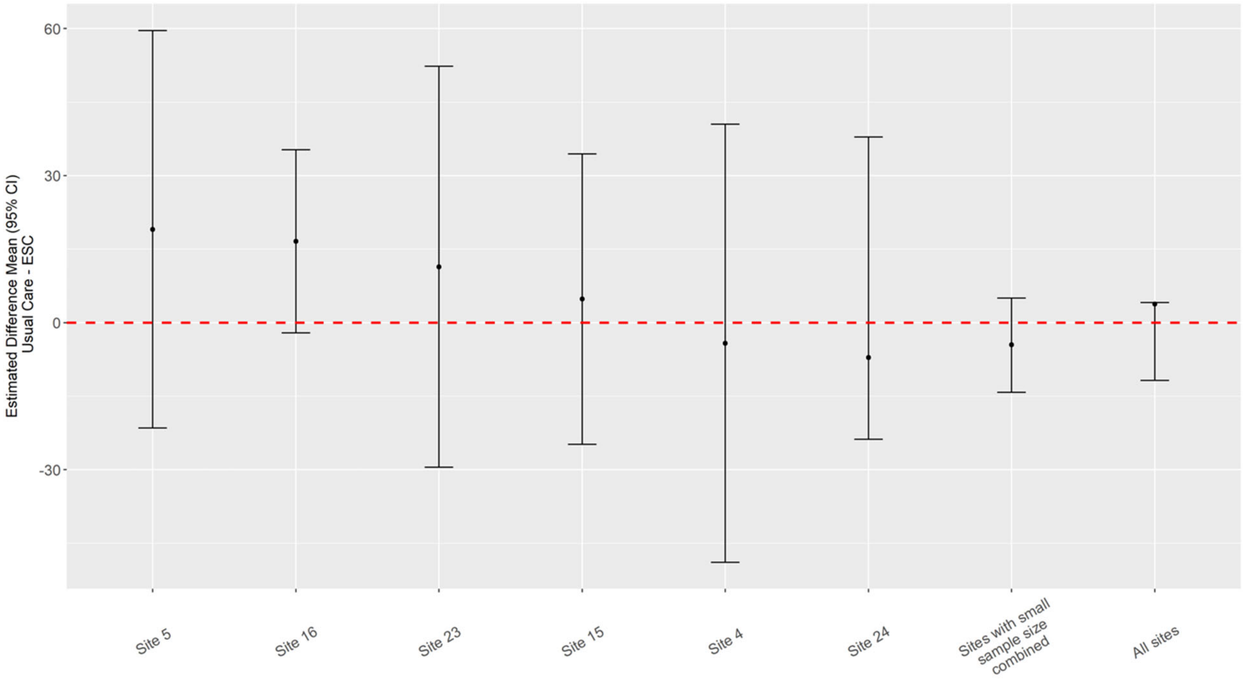

© 2024 Devlin LA et al. *JAMA Pediatrics.*

eMethods 2. Statistical Analysis Outline

We used the following general model-building approach for our analyses to account for the stepped-wedge study design. For each outcome, we follow four analysis steps: 1) an unadjusted before/after of the effect of the ESC care approach (ignoring period/time effect); 2) the time period (i.e., steps) to examine if any potential intervention effect relates only to the intervention or also to an independent effect of calendar time; 3) an adjustment for infant-level and maternal characteristics, stratification indicator (proportion of infants with NOWS treated pharmacologically), and potential site-level confounders, such as hospital volume and rural/urban indicator; 4) the possible interaction between period and intervention effect. The impact of the ESC care approach on the outcome could potentially change over time, as the improvement in outcome could increase with time as the staff gains experience. However, the impact could also decrease after an initial improvement as the level of initial enthusiasm decreases. We aim to explore this question through the inclusion of an interaction between period/time and intervention effect in Model 4.

Model Building

Using the following notation:

- $\beta_j =$ fixed effect of time/period
- $X_{ij} = 1$ if ESC, 0 if Usual Care
- $\theta =$ intervention effect
- $u_i =$ random effect for cluster/site
- $C_l =$ patient-level adjustment variables
- $\omega =$ fixed effects for patient-level demographics
- $\varepsilon_{ijl} =$ residual
- $i = 1,...,k$ clusters/sites
- $j = 1,...,T$ periods
- $l = 1,...,m$ infants per cluster per period (cross-sectional cohort)

First, we will start with an unadjusted before/after analysis of the effect of ESC (ignoring time effect).

MODEL-1 $Y_{il} = \mu + \theta X_i + u_i + \varepsilon_{il}$,
where Y_{il} = the length of time until medically ready for discharge for infant *l* in cluster *i*.

Second, we'll build in the effect of time/period (step) to investigate if any potential treatment effect is related only to the intervention or also to an independent effect of calendar time.

© 2024 Devlin LA et al. *JAMA Pediatrics.*

MODEL-2 $Y_{ijl} = \mu + \theta X_{ij} + \beta_j + u_i + \varepsilon_{ijl}$,

where β_j is the effect of *j*th time/period where *j* = 1,2,…,10. Calendar time could be a potential confounder as other factors/events (e.g., other changes in clinical practice) could influence the outcome measure in both the usual care and ESC infants. As the effect could be anything from absent, or gradual (progressive slow trend) to abrupt, (near simultaneous adoption of a new practice that has an immediate full-strength effect), calendar time will be fitted in the model first as categorical and then as a linear variable, and appropriate fitting will be chose.

Third, we adjust for patient-level characteristics.

MODEL-3 $Y_{ijl} = \mu + \theta X_{ij} + \beta_j + \omega C_{ijl} + u_i + \varepsilon_{ijl}$,

where ω is a vector of coefficients for a matrix of *C* covariates for the infant *l* in site *i* at time/period *j*. The covariates of adjustment used in this analysis include the following: gestational age, birth weight, and race/ethnicity, inborn, adequate prenatal care, medication for opioid use disorder, polysubstance exposures, rural-urban commuting area code (RUCA), pharmacologic treatment medication types. Additionally, we adjust for the randomization scheme stratification indicator (proportion of infants with NOWS treated pharmacologically at each site: lowest 3rd, middle 3rd, highest 3rd).

Finally, time will be fitted as an intervention effect modifier to examine how the impact of the intervention develops over time.

MODEL-4 $Y_{ijl} = \mu + \theta X_{ij} + \beta_j + \omega C_{ijl} + \gamma Q_{ij} + u_i + \varepsilon_{ijl}$,

where γ is the parameter estimate for the interaction between time and intervention (variable *Q*, analyzed as numerical variable (0 = any control period, 1 = 1st step, 2 = 2nd step, etc.) for site *i* at time *j*.

<u>Outcome Specific Modeling</u>

For outcomes including total number of opioid doses, length of opioid treatment, and length of hospital stay, we used generalized linear mixed models (GLMM) with negative binomial distribution.

We used GLMM with gamma distribution to examine the effect of the ESC care approach on peak opioid dose and total postnatal opioid exposure (MME/kg) reporting group means, absolute mean difference, incidence rate ratio (IRR), and 95% confidence intervals (CI).

For receipt of adjuvant therapy, we used mixed-effect Poisson regression with robust error variance, reporting adjusted relative risk ratio (RR) with 95% CI.

© 2024 Devlin LA et al. *JAMA Pediatrics.*

As noted in our general modeling approach described above, all regression models accounted for the stepped-wedge design with intervention and time as fixed effects and sites as random effects along with the strata indicator (proportion of infants treated pharmacologically at each site prior to trial initiation according to lowest third, middle third, and highest third).

© 2024 Devlin LA et al. *JAMA Pediatrics.*
