# Supplementary material for: Influence of Eat, Sleep, and Console on Infants Pharmacologically Treated for Opioid Withdrawal: A Post Hoc Subgroup Analysis of the ESC-NOW Randomized Clinical Trial
Source: JAMA Pediatr. 2024 Apr 15;178(6):525–32. doi: 10.1001/jamapediatrics.2024.0544 (PMC11019446; doi:10.1001/jamapediatrics.2024.0544)
Supplement: Supplement 3. — Nonauthor collaborators [file jamapediatr-e240544-s003.pdf]

Supplemental Online Content: Nonauthor Collaborators

*Indicates required information. Only first name, last name, and suffix will appear in PubMed.

*Group Name(s): Eunice Kennedy Shriver National Institute of Child Health and Human Development Neonatal Research Network and NIH Environmental influences on Child Health Outcomes (ECHO) Program Institutional Development Award States Pediatric Clinical Trials Network							
***First Name and Middle Initial(s)**	***Last Name**	***Suffix (eg, Jr, III)**	Academic Degrees	Institution	Location (city, state/province, country)	Role or Contribution, eg, chair, principal investigator	Group (if more than 1 Group listed in the byline) and/or Subgroup (eg, Steering Committee)
De Ann	Hubberd		MA, CCRP	University of Arkansas	Little Rock, AK	ESC Training Team	NIH Environmental influences on Child Health Outcomes (ECHO) Program Institutional Development Award States Pediatric Clinical Trials Network
Kathy	Edwards		BSN, RN, CCRP, CCRA	University of Arkansas	Little Rock, AK	ESC Training Team	NIH Environmental influences on Child Health Outcomes (ECHO) Program Institutional Development Award States Pediatric Clinical Trials Network
Bonny L.	Whalen		MD	Dartmouth Hitchcock Medical Center	Hanover, NH	ESC Faculty	NIH Environmental influences on Child Health Outcomes (ECHO) Program Institutional Development Award States Pediatric Clinical Trials Network
Kate	MacMillan		MD, MPH	Mass General for Children: Pediatric Hospital	Boston, MA	ESC Faculty	NIH Environmental influences on Child Health Outcomes (ECHO) Program Institutional Development Award States Pediatric Clinical Trials Network

*Indicates required information. Only first name, last name, and suffix will appear in PubMed.

*First Name and Middle Initial(s)	*Last Name	*Suffix (eg, Jr, III)	Academic Degrees	Institution	Location (city, state/province, country)	Role or Contribution, eg, chair, principal investigator	Group (if more than 1 Group listed in the byline) and/or Subgroup (eg, Steering Committee)
Adrienne	Pahl		MD	Department of Pediatrics, University of Vermont Larner College of Medicine	Burlington, VT	ESC Faculty	NIH Environmental influences on Child Health Outcomes (ECHO) Program Institutional Development Award States Pediatric Clinical Trials Network
Farrah	Sheehan Desselle		MSN, RN, IBCLC, CCBE(BFW)	Dartmouth College	Hanover, NH	ESC Faculty	NIH Environmental influences on Child Health Outcomes (ECHO) Program Institutional Development Award States Pediatric Clinical Trials Network
Katie	White		RN	Wentworth-Douglass Hospital	Dover, NH	ESC Faculty	NIH Environmental influences on Child Health Outcomes (ECHO) Program Institutional Development Award States Pediatric Clinical Trials Network
Anna Marie	Hibbs		MD, MSCE, FAAP	Case Western Reserve University, Rainbow Babies & Children's Hospital	Cleveland, OH	Study Site Contributor	NIH Eunice Kennedy Shriver National Institute of Child Health and Human Development Neonatal Research Network
Leslie	Clarke		RN, MS, MBA	Case Western Reserve University, Rainbow Babies & Children's Hospital	Cleveland, OH	Study Site Contributor	NIH Eunice Kennedy Shriver National Institute of Child Health and Human Development Neonatal Research Network

Supplemental Online Content: Nonauthor Collaborators

*Indicates required information. Only first name, last name, and suffix will appear in PubMed.

***First Name and Middle Initial(s)**	***Last Name**	***Suffix (eg, Jr, III)**	Academic Degrees	Institution	Location (city, state/province, country)	Role or Contribution, eg, chair, principal investigator	Group (if more than 1 Group listed in the byline) and/or Subgroup (eg, Steering Committee)
Kathy	Serio		RNC-LRN, BSN	Case Western Reserve University, Rainbow Babies & Children's Hospital	Cleveland, OH	Study Site Contributor	NIH Eunice Kennedy Shriver National Institute of Child Health and Human Development Neonatal Research Network
Maria	Kertesz		RN, MSN	Case Western Reserve University, Rainbow Babies & Children's Hospital	Cleveland, OH	Study Site Contributor	NIH Eunice Kennedy Shriver National Institute of Child Health and Human Development Neonatal Research Network
Ashley	Rodriguez		MSN, APRN, NNP- BC	Case Western Reserve University, Rainbow Babies & Children's Hospital	Cleveland, OH	Study Site Contributor	NIH Eunice Kennedy Shriver National Institute of Child Health and Human Development Neonatal Research Network
Sarah	Driscoll		RNC-LRN, BSN	Case Western Reserve University, Rainbow Babies & Children's Hospital	Cleveland, OH	Study Site Contributor	NIH Eunice Kennedy Shriver National Institute of Child Health and Human Development Neonatal Research Network
Kim	Hammond		RNC-LRN, BSN	Case Western Reserve University, Rainbow Babies & Children's Hospital	Cleveland, OH	Study Site Contributor	NIH Eunice Kennedy Shriver National Institute of Child Health and Human Development Neonatal Research Network

Supplemental Online Content: Nonauthor Collaborators

*Indicates required information. Only first name, last name, and suffix will appear in PubMed.

***First Name and Middle Initial(s)**	***Last Name**	***Suffix (eg, Jr, III)**	Academic Degrees	Institution	Location (city, state/province, country)	Role or Contribution, eg, chair, principal investigator	Group (if more than 1 Group listed in the byline) and/or Subgroup (eg, Steering Committee)
Deborah	Hines		RN, BSN	Case Western Reserve University, Rainbow Babies & Children's Hospital	Cleveland, OH	Study Site Contributor	NIH Eunice Kennedy Shriver National Institute of Child Health and Human Development Neonatal Research Network
Patricia	Kish		RN, BS	Case Western Reserve University, Rainbow Babies & Children's Hospital	Cleveland, OH	Study Site Contributor	NIH Eunice Kennedy Shriver National Institute of Child Health and Human Development Neonatal Research Network
Taylor	Schmidt		RN, BSN	Case Western Reserve University, Rainbow Babies & Children's Hospital	Cleveland, OH	Study Site Contributor	NIH Eunice Kennedy Shriver National Institute of Child Health and Human Development Neonatal Research Network
Jennifer	Stepp		RN, BSN	Case Western Reserve University, Rainbow Babies & Children's Hospital	Cleveland, OH	Study Site Contributor	NIH Eunice Kennedy Shriver National Institute of Child Health and Human Development Neonatal Research Network
Jodi	Jackson		MD	Children's Mercy Hospital and Advent Health – Shawnee Mission	Overland Park, KS	Study Site Contributor	NIH Environmental influences on Child Health Outcomes (ECHO) Program Institutional Development Award States Pediatric Clinical Trials Network

*Indicates required information. Only first name, last name, and suffix will appear in PubMed.

*First Name and Middle Initial(s)	*Last Name	*Suffix (eg, Jr, III)	Academic Degrees	Institution	Location (city, state/province, country)	Role or Contribution, eg, chair, principal investigator	Group (if more than 1 Group listed in the byline) and/or Subgroup (eg, Steering Committee)
Allie	Scott		RN, CCRC	Children's Mercy Hospital and Advent Health – Shawnee Mission	Overland Park, KS	Study Site Contributor	NIH Environmental influences on Child Health Outcomes (ECHO) Program Institutional Development Award States Pediatric Clinical Trials Network
Rochelle	Brown		RN, MSN	Children's Mercy Hospital and Advent Health – Shawnee Mission	Overland Park, KS	Study Site Contributor	NIH Environmental influences on Child Health Outcomes (ECHO) Program Institutional Development Award States Pediatric Clinical Trials Network
Kari	Smith		RN, RNC-OB, C-EFM, MSN	Children's Mercy Hospital and Advent Health – Shawnee Mission	Overland Park, KS	Study Site Contributor	NIH Environmental influences on Child Health Outcomes (ECHO) Program Institutional Development Award States Pediatric Clinical Trials Network
My	Rieper		RN, CPN, MSN	Children's Mercy Hospital and Advent Health – Shawnee Mission	Overland Park, KS	Study Site Contributor	NIH Environmental influences on Child Health Outcomes (ECHO) Program Institutional Development Award States Pediatric Clinical Trials Network
Carrie	Miner		RN	Children's Mercy Hospital and Advent Health – Shawnee Mission	Overland Park, KS	Study Site Contributor	NIH Environmental influences on Child Health Outcomes (ECHO) Program Institutional Development Award States Pediatric Clinical Trials Network

Supplemental Online Content: Nonauthor Collaborators

*Indicates required information. Only first name, last name, and suffix will appear in PubMed.

***First Name and Middle Initial(s)**	***Last Name**	***Suffix (eg, Jr, III)**	Academic Degrees	Institution	Location (city, state/province, country)	Role or Contribution, eg, chair, principal investigator	Group (if more than 1 Group listed in the byline) and/or Subgroup (eg, Steering Committee)
Lynne	Ray			Children's Mercy Hospital and Advent Health – Shawnee Mission	Overland Park, KS	Study Site Contributor	NIH Environmental influences on Child Health Outcomes (ECHO) Program Institutional Development Award States Pediatric Clinical Trials Network
Karen	Kowel		PAC	Christiana Care Health Systems	Newark, DE	Study Site Contributor	NIH Environmental influences on Child Health Outcomes (ECHO) Program Institutional Development Award States Pediatric Clinical Trials Network
Victoria	Reynolds		BA	Christiana Care Health Systems	Newark, DE	Study Site Contributor	NIH Environmental influences on Child Health Outcomes (ECHO) Program Institutional Development Award States Pediatric Clinical Trials Network
Diana	Castellone		BA	Christiana Care Health Systems	Newark, DE	Study Site Contributor	NIH Environmental influences on Child Health Outcomes (ECHO) Program Institutional Development Award States Pediatric Clinical Trials Network
Amy	Mackley			Christiana Care Health Systems	Newark, DE	Study Site Contributor	NIH Environmental influences on Child Health Outcomes (ECHO) Program Institutional Development Award States Pediatric Clinical Trials Network

*Indicates required information. Only first name, last name, and suffix will appear in PubMed.

***First Name and Middle Initial(s)**	***Last Name**	***Suffix (eg, Jr, III)**	Academic Degrees	Institution	Location (city, state/province, country)	Role or Contribution, eg, chair, principal investigator	Group (if more than 1 Group listed in the byline) and/or Subgroup (eg, Steering Committee)
Kurt	Schibler		MD	Cincinnati Children's Hospital Medical Center, University Hospital, St. Elizabeth and Good Samaritan Hospital	Cincinnati, OH	Study Site Contributor	NIH Eunice Kennedy Shriver National Institute of Child Health and Human Development Neonatal Research Network
Traci	Beiersdorfer		RN, BSN	Cincinnati Children's Hospital Medical Center, University Hospital, St. Elizabeth and Good Samaritan Hospital	Cincinnati, OH	Study Site Contributor	NIH Eunice Kennedy Shriver National Institute of Child Health and Human Development Neonatal Research Network
Cathy	Grisby		RN,BSN, CCRC	Cincinnati Children's Hospital Medical Center, University Hospital, St. Elizabeth and Good Samaritan Hospital	Cincinnati, OH	Study Site Contributor	NIH Eunice Kennedy Shriver National Institute of Child Health and Human Development Neonatal Research Network
Kristin	Kirker		CRC	Cincinnati Children's Hospital Medical Center, University Hospital, St. Elizabeth and Good Samaritan Hospital	Cincinnati, OH	Study Site Contributor	NIH Eunice Kennedy Shriver National Institute of Child Health and Human Development Neonatal Research Network
Katherine	McKeown		RNC-NIC, BSN	Cincinnati Children's Hospital Medical Center, University Hospital, St. Elizabeth and Good Samaritan Hospital	Cincinnati, OH	Study Site Contributor	NIH Eunice Kennedy Shriver National Institute of Child Health and Human Development Neonatal Research Network
M. Katherine	Loudermilk		MD	Cincinnati Children's Hospital Medical Center, University Hospital, St. Elizabeth and Good Samaritan Hospital	Cincinnati, OH	Study Site Contributor	NIH Eunice Kennedy Shriver National Institute of Child Health and Human Development Neonatal Research Network

Supplemental Online Content: Nonauthor Collaborators

*Indicates required information. Only first name, last name, and suffix will appear in PubMed.

***First Name and Middle Initial(s)**	***Last Name**	***Suffix (eg, Jr, III)**	Academic Degrees	Institution	Location (city, state/province, country)	Role or Contribution, eg, chair, principal investigator	Group (if more than 1 Group listed in the byline) and/or Subgroup (eg, Steering Committee)
Amy	Carnohan		RN, MSN	Cincinnati Children's Hospital Medical Center, University Hospital, St. Elizabeth and Good Samaritan Hospital	Cincinnati, OH	Study Site Contributor	NIH Eunice Kennedy Shriver National Institute of Child Health and Human Development Neonatal Research Network
Patti	Froese		NNP-BC, DNP	Cincinnati Children's Hospital Medical Center, University Hospital, St. Elizabeth and Good Samaritan Hospital	Cincinnati, OH	Study Site Contributor	NIH Eunice Kennedy Shriver National Institute of Child Health and Human Development Neonatal Research Network
April	Haddix		RN	Cincinnati Children's Hospital Medical Center, University Hospital, St. Elizabeth and Good Samaritan Hospital	Cincinnati, OH	Study Site Contributor	NIH Eunice Kennedy Shriver National Institute of Child Health and Human Development Neonatal Research Network
Virginia	Summe		RN	Cincinnati Children's Hospital Medical Center, University Hospital, St. Elizabeth and Good Samaritan Hospital	Cincinnati, OH	Study Site Contributor	NIH Eunice Kennedy Shriver National Institute of Child Health and Human Development Neonatal Research Network
Clara	Chlon		MD	Cincinnati Children's Hospital Medical Center, University Hospital, St. Elizabeth and Good Samaritan Hospital	Cincinnati, OH	Study Site Contributor	NIH Eunice Kennedy Shriver National Institute of Child Health and Human Development Neonatal Research Network
Rita	Kunk		CNP	Cincinnati Children's Hospital Medical Center, University Hospital, St. Elizabeth and Good Samaritan Hospital	Cincinnati, OH	Study Site Contributor	NIH Eunice Kennedy Shriver National Institute of Child Health and Human Development Neonatal Research Network

*Indicates required information. Only first name, last name, and suffix will appear in PubMed.

***First Name and Middle Initial(s)**	***Last Name**	***Suffix (eg, Jr, III)**	Academic Degrees	Institution	Location (city, state/province, country)	Role or Contribution, eg, chair, principal investigator	Group (if more than 1 Group listed in the byline) and/or Subgroup (eg, Steering Committee)
Jen	Hamilton		RN, VA-BC, BSN	Cincinnati Children's Hospital Medical Center, University Hospital, St. Elizabeth and Good Samaritan Hospital	Cincinnati, OH	Study Site Contributor	NIH Eunice Kennedy Shriver National Institute of Child Health and Human Development Neonatal Research Network
Sadie	Moore			Cincinnati Children's Hospital Medical Center, University Hospital, St. Elizabeth and Good Samaritan Hospital	Cincinnati, OH	Study Site Contributor	NIH Eunice Kennedy Shriver National Institute of Child Health and Human Development Neonatal Research Network
Angela	Ziegelmeyer		RN, BSN	Cincinnati Children's Hospital Medical Center, University Hospital, St. Elizabeth and Good Samaritan Hospital	Cincinnati, OH	Study Site Contributor	NIH Eunice Kennedy Shriver National Institute of Child Health and Human Development Neonatal Research Network
Lucy	Rolfes		RN, BSN	Cincinnati Children's Hospital Medical Center, University Hospital, St. Elizabeth and Good Samaritan Hospital	Cincinnati, OH	Study Site Contributor	NIH Eunice Kennedy Shriver National Institute of Child Health and Human Development Neonatal Research Network
Erica	Schomaker		CLC, BSN	Cincinnati Children's Hospital Medical Center, University Hospital, St. Elizabeth and Good Samaritan Hospital	Cincinnati, OH	Study Site Contributor	NIH Eunice Kennedy Shriver National Institute of Child Health and Human Development Neonatal Research Network

*Indicates required information. Only first name, last name, and suffix will appear in PubMed.

*First Name and Middle Initial(s)	*Last Name	*Suffix (eg, Jr, III)	Academic Degrees	Institution	Location (city, state/province, country)	Role or Contribution, eg, chair, principal investigator	Group (if more than 1 Group listed in the byline) and/or Subgroup (eg, Steering Committee)
Holly	Strike		MD	Cincinnati Children's Hospital Medical Center, University Hospital, St. Elizabeth and Good Samaritan Hospital	Cincinnati, OH	Study Site Contributor	NIH Eunice Kennedy Shriver National Institute of Child Health and Human Development Neonatal Research Network
Sharon	Harvey		RNC-NIC, BSN	Cincinnati Children's Hospital Medical Center, University Hospital, St. Elizabeth and Good Samaritan Hospital	Cincinnati, OH	Study Site Contributor	NIH Eunice Kennedy Shriver National Institute of Child Health and Human Development Neonatal Research Network
Paula	Richards		RNC-OB, C-EFM, MSN	Cincinnati Children's Hospital Medical Center, University Hospital, St. Elizabeth and Good Samaritan Hospital	Cincinnati, OH	Study Site Contributor	NIH Eunice Kennedy Shriver National Institute of Child Health and Human Development Neonatal Research Network
Ashley	Simon		RNC-NIC, MSN	Cincinnati Children's Hospital Medical Center, University Hospital, St. Elizabeth and Good Samaritan Hospital	Cincinnati, OH	Study Site Contributor	NIH Eunice Kennedy Shriver National Institute of Child Health and Human Development Neonatal Research Network
David	Russell		JD	Cincinnati Children's Hospital Medical Center, University Hospital, St. Elizabeth and Good Samaritan Hospital	Cincinnati, OH	Study Site Contributor	NIH Eunice Kennedy Shriver National Institute of Child Health and Human Development Neonatal Research Network

Supplemental Online Content: Nonauthor Collaborators

*Indicates required information. Only first name, last name, and suffix will appear in PubMed.

***First Name and Middle Initial(s)**	***Last Name**	***Suffix (eg, Jr, III)**	Academic Degrees	Institution	Location (city, state/province, country)	Role or Contribution, eg, chair, principal investigator	Group (if more than 1 Group listed in the byline) and/or Subgroup (eg, Steering Committee)
Christine A.	Gleason		MD	University of Washington	Seattle, WA	Data and Safety Monitoring Committee	NIH Eunice Kennedy Shriver National Institute of Child Health and Human Development Neonatal Research Network
Marilee C.	Allen		MD	Johns Hopkins University School of Medicine	Baltimore, MD	Data and Safety Monitoring Committee	NIH Eunice Kennedy Shriver National Institute of Child Health and Human Development Neonatal Research Network
Robert J.	Boyle		MD	University of Virginia Health System	Charlottesville, VA	Data and Safety Monitoring Committee	NIH Eunice Kennedy Shriver National Institute of Child Health and Human Development Neonatal Research Network
Traci	Clemons		PhD	The EMMES Corporation	Fredrick, MD	Data and Safety Monitoring Committee	NIH Eunice Kennedy Shriver National Institute of Child Health and Human Development Neonatal Research Network
Mary E.	D’Alton		MD	Columbia Ob/Gyn Midtown	New York, NY	Data and Safety Monitoring Committee	NIH Eunice Kennedy Shriver National Institute of Child Health and Human Development Neonatal Research Network

*Indicates required information. Only first name, last name, and suffix will appear in PubMed.

***First Name and Middle Initial(s)**	***Last Name**	***Suffix (eg, Jr, III)**	Academic Degrees	Institution	Location (city, state/province, country)	Role or Contribution, eg, chair, principal investigator	Group (if more than 1 Group listed in the byline) and/or Subgroup (eg, Steering Committee)
Robin	Steinhorn		MD	University of California San Diego	San Diego, CA	Data and Safety Monitoring Committee	NIH Eunice Kennedy Shriver National Institute of Child Health and Human Development Neonatal Research Network
Steven J.	Weiner		MS	The George Washington University	Washington, DC	Data and Safety Monitoring Committee	NIH Eunice Kennedy Shriver National Institute of Child Health and Human Development Neonatal Research Network
Carol J.	Blaisdell		MD	Environmental Influence on Child Health Outcomes (ECHO)	Bethesda, MD	Deputy Director ECHO Program	NIH Environmental influences on Child Health Outcomes (ECHO) Program Institutional Development Award States Pediatric Clinical Trials Network
Stephanie	Wilson Archer		MA	*Eunice Kennedy Shriver* National Institute of Child Health and Human Development	Bethesda, MD	Program Analyst	NIH Eunice Kennedy Shriver National Institute of Child Health and Human Development Neonatal Research Network
Annette	Amiotte		BSN	Kapiolani Medical Center for Women and Children	Honolulu, HI	Study Site Contributor	NIH Environmental influences on Child Health Outcomes (ECHO) Program Institutional Development Award States Pediatric Clinical Trials Network

Supplemental Online Content: Nonauthor Collaborators

*Indicates required information. Only first name, last name, and suffix will appear in PubMed.

***First Name and Middle Initial(s)**	***Last Name**	***Suffix (eg, Jr, III)**	Academic Degrees	Institution	Location (city, state/province, country)	Role or Contribution, eg, chair, principal investigator	Group (if more than 1 Group listed in the byline) and/or Subgroup (eg, Steering Committee)
Moara	Santos		PhD	Kapiolani Medical Center for Women and Children	Honolulu, HI	Study Site Contributor	NIH Environmental influences on Child Health Outcomes (ECHO) Program Institutional Development Award States Pediatric Clinical Trials Network
Jill	Taosaka		OTR	Kapiolani Medical Center for Women and Children	Honolulu, HI	Study Site Contributor	NIH Environmental influences on Child Health Outcomes (ECHO) Program Institutional Development Award States Pediatric Clinical Trials Network
Charles	Neal		MD	Kapiolani Medical Center for Women and Children	Honolulu, HI	Study Site Contributor	NIH Environmental influences on Child Health Outcomes (ECHO) Program Institutional Development Award States Pediatric Clinical Trials Network
Andrew M.	Atz		MD	MUSC Health Shawn Jenkins Children’s Hospital	Charleston, SC	Study Site Contributor	NIH Environmental influences on Child Health Outcomes (ECHO) Program Institutional Development Award States Pediatric Clinical Trials Network
Andrea	Summer		MD, MSCR	MUSC Health Shawn Jenkins Children’s Hospital	Charleston, SC	Study Site Contributor	NIH Environmental influences on Child Health Outcomes (ECHO) Program Institutional Development Award States Pediatric Clinical Trials Network

*Indicates required information. Only first name, last name, and suffix will appear in PubMed.

*First Name and Middle Initial(s)	*Last Name	*Suffix (eg, Jr, III)	Academic Degrees	Institution	Location (city, state/province, country)	Role or Contribution, eg, chair, principal investigator	Group (if more than 1 Group listed in the byline) and/or Subgroup (eg, Steering Committee)
Michelle	Amaya		MD, MPH	MUSC Health Shawn Jenkins Children's Hospital	Charleston, SC	Study Site Contributor	NIH Environmental influences on Child Health Outcomes (ECHO) Program Institutional Development Award States Pediatric Clinical Trials Network
Mary	Freeman		CCRP	MUSC Health Shawn Jenkins Children's Hospital	Charleston, SC	Study Site Contributor	NIH Environmental influences on Child Health Outcomes (ECHO) Program Institutional Development Award States Pediatric Clinical Trials Network
Madison	Johnson		CCRP	MUSC Health Shawn Jenkins Children's Hospital	Charleston, SC	Study Site Contributor	NIH Environmental influences on Child Health Outcomes (ECHO) Program Institutional Development Award States Pediatric Clinical Trials Network
Layla Al	Sarraf		CCRP	MUSC Health Shawn Jenkins Children's Hospital	Charleston, SC	Study Site Contributor	NIH Environmental influences on Child Health Outcomes (ECHO) Program Institutional Development Award States Pediatric Clinical Trials Network
Rosemary	Ros-Demarize		PhD	MUSC Health Shawn Jenkins Children's Hospital	Charleston, SC	Study Site Contributor	NIH Environmental influences on Child Health Outcomes (ECHO) Program Institutional Development Award States Pediatric Clinical Trials Network

*Indicates required information. Only first name, last name, and suffix will appear in PubMed.

***First Name and Middle Initial(s)**	***Last Name**	***Suffix (eg, Jr, III)**	Academic Degrees	Institution	Location (city, state/province, country)	Role or Contribution, eg, chair, principal investigator	Group (if more than 1 Group listed in the byline) and/or Subgroup (eg, Steering Committee)
Pablo J.	Sánchez		MD	Nationwide Children’s Hospital, The Abigail Wexner Research Institute at Nationwide Children’s Hospital, Center for Perinatal [illegible]	Columbus, OH	Study Site Contributor	NIH Eunice Kennedy Shriver National Institute of Child Health and Human Development Neonatal Research Network
Jonathan L.	Slaughter		MD	Nationwide Children’s Hospital, The Abigail Wexner Research Institute at Nationwide Children’s Hospital, Center for Perinatal Research, The Ohio State University College of Medicine	Columbus, OH	Study Site Contributor	NIH Eunice Kennedy Shriver National Institute of Child Health and Human Development Neonatal Research Network
Kristen	Benninger		MD	Nationwide Children’s Hospital, The Abigail Wexner Research Institute at Nationwide Children’s Hospital, Center for Perinatal Research, The Ohio State University College of Medicine	Columbus, OH	Study Site Contributor	NIH Eunice Kennedy Shriver National Institute of Child Health and Human Development Neonatal Research Network
Patricia	Luzader		RN	Nationwide Children’s Hospital, The Abigail Wexner Research Institute at Nationwide Children’s Hospital, Center for Perinatal Research, The Ohio State University College of Medicine	Columbus, OH	Study Site Contributor	NIH Eunice Kennedy Shriver National Institute of Child Health and Human Development Neonatal Research Network

Supplemental Online Content: Nonauthor Collaborators

*Indicates required information. Only first name, last name, and suffix will appear in PubMed.

***First Name and Middle Initial(s)**	***Last Name**	***Suffix (eg, Jr, III)**	Academic Degrees	Institution	Location (city, state/province, country)	Role or Contribution, eg, chair, principal investigator	Group (if more than 1 Group listed in the byline) and/or Subgroup (eg, Steering Committee)
Laura	Marzec		IMG	Nationwide Children's Hospital, The Abigail Wexner Research Institute at Nationwide Children's Hospital, Center for Perinatal Research, The Ohio State University College of Medicine	Columbus, OH	Study Site Contributor	NIH Eunice Kennedy Shriver National Institute of Child Health and Human Development Neonatal Research Network
Brittany	DeSantis		BS	Nationwide Children's Hospital, The Abigail Wexner Research Institute at Nationwide Children's Hospital, Center for Perinatal Research, The Ohio State University College of Medicine	Columbus, OH	Study Site Contributor	NIH Eunice Kennedy Shriver National Institute of Child Health and Human Development Neonatal Research Network
Kristi	Small		BS	Nationwide Children's Hospital, The Abigail Wexner Research Institute at Nationwide Children's Hospital, Center for Perinatal Research, The Ohio State University College of Medicine	Columbus, OH	Study Site Contributor	NIH Eunice Kennedy Shriver National Institute of Child Health and Human Development Neonatal Research Network

*Indicates required information. Only first name, last name, and suffix will appear in PubMed.

***First Name and Middle Initial(s)**	***Last Name**	***Suffix (eg, Jr, III)**	Academic Degrees	Institution	Location (city, state/province, country)	Role or Contribution, eg, chair, principal investigator	Group (if more than 1 Group listed in the byline) and/or Subgroup (eg, Steering Committee)
Julia	Newton		MPH	Nationwide Children's Hospital, The Abigail Wexner Research Institute at Nationwide Children's Hospital, Center for Perinatal Research, The Ohio State University College of Medicine	Columbus, OH	Study Site Contributor	NIH Eunice Kennedy Shriver National Institute of Child Health and Human Development Neonatal Research Network
Jessica	Purnell		CCRC, BS	Nationwide Children's Hospital, The Abigail Wexner Research Institute at Nationwide Children's Hospital, Center for Perinatal Research, The Ohio State University College of Medicine	Columbus, OH	Study Site Contributor	NIH Eunice Kennedy Shriver National Institute of Child Health and Human Development Neonatal Research Network
Rachel	Reedy		RN	Nationwide Children's Hospital, The Abigail Wexner Research Institute at Nationwide Children's Hospital, Center for Perinatal Research, The Ohio State University College of Medicine	Columbus, OH	Study Site Contributor	NIH Eunice Kennedy Shriver National Institute of Child Health and Human Development Neonatal Research Network

Supplemental Online Content: Nonauthor Collaborators

*Indicates required information. Only first name, last name, and suffix will appear in PubMed.

***First Name and Middle Initial(s)**	***Last Name**	***Suffix (eg, Jr, III)**	Academic Degrees	Institution	Location (city, state/province, country)	Role or Contribution, eg, chair, principal investigator	Group (if more than 1 Group listed in the byline) and/or Subgroup (eg, Steering Committee)
Cory	Hanlon		BS	Nationwide Children's Hospital, The Abigail Wexner Research Institute at Nationwide Children's Hospital, Center for Perinatal Research, The Ohio State University College of Medicine	Columbus, OH	Study Site Contributor	NIH Eunice Kennedy Shriver National Institute of Child Health and Human Development Neonatal Research Network
Tanvi	Naik		BS	Nationwide Children's Hospital, The Abigail Wexner Research Institute at Nationwide Children's Hospital, Center for Perinatal Research, The Ohio State University College of Medicine	Columbus, OH	Study Site Contributor	NIH Eunice Kennedy Shriver National Institute of Child Health and Human Development Neonatal Research Network
Jordan	Knox		BS	Nationwide Children's Hospital, The Abigail Wexner Research Institute at Nationwide Children's Hospital, Center for Perinatal Research, The Ohio State University College of Medicine	Columbus, OH	Study Site Contributor	NIH Eunice Kennedy Shriver National Institute of Child Health and Human Development Neonatal Research Network

*Indicates required information. Only first name, last name, and suffix will appear in PubMed.

***First Name and Middle Initial(s)**	***Last Name**	***Suffix (eg, Jr, III)**	Academic Degrees	Institution	Location (city, state/province, country)	Role or Contribution, eg, chair, principal investigator	Group (if more than 1 Group listed in the byline) and/or Subgroup (eg, Steering Committee)
Aishwarya	Tallikar		BS	Nationwide Children's Hospital, The Abigail Wexner Research Institute at Nationwide Children's Hospital, Center for Perinatal Research, The Ohio State University College of Medicine	Columbus, OH	Study Site Contributor	NIH Eunice Kennedy Shriver National Institute of Child Health and Human Development Neonatal Research Network
Chelsea	Cobe		BA	Nationwide Children's Hospital, The Abigail Wexner Research Institute at Nationwide Children's Hospital, Center for Perinatal Research, The Ohio State University College of Medicine	Columbus, OH	Study Site Contributor	NIH Eunice Kennedy Shriver National Institute of Child Health and Human Development Neonatal Research Network
Megan	Resetar		RN	Nationwide Children's Hospital, The Abigail Wexner Research Institute at Nationwide Children's Hospital, Center for Perinatal Research, The Ohio State University College of Medicine	Columbus, OH	Study Site Contributor	NIH Eunice Kennedy Shriver National Institute of Child Health and Human Development Neonatal Research Network

*Indicates required information. Only first name, last name, and suffix will appear in PubMed.

***First Name and Middle Initial(s)**	***Last Name**	***Suffix (eg, Jr, III)**	Academic Degrees	Institution	Location (city, state/province, country)	Role or Contribution, eg, chair, principal investigator	Group (if more than 1 Group listed in the byline) and/or Subgroup (eg, Steering Committee)
Jacqueline	McCool			Nationwide Children's Hospital, The Abigail Wexner Research Institute at Nationwide Children's Hospital, Center for Perinatal Research, The Ohio State University College of Medicine	Columbus, OH	Study Site Contributor	NIH Eunice Kennedy Shriver National Institute of Child Health and Human Development Neonatal Research Network
Erin	Frailey		RN	Nationwide Children's Hospital, The Abigail Wexner Research Institute at Nationwide Children's Hospital, Center for Perinatal Research, The Ohio State University College of Medicine	Columbus, OH	Study Site Contributor	NIH Eunice Kennedy Shriver National Institute of Child Health and Human Development Neonatal Research Network
Stephanie	Jones		RN	Nationwide Children's Hospital, The Abigail Wexner Research Institute at Nationwide Children's Hospital, Center for Perinatal Research, The Ohio State University College of Medicine	Columbus, OH	Study Site Contributor	NIH Eunice Kennedy Shriver National Institute of Child Health and Human Development Neonatal Research Network

Supplemental Online Content: Nonauthor Collaborators

*Indicates required information. Only first name, last name, and suffix will appear in PubMed.

*First Name and Middle Initial(s)	*Last Name	*Suffix (eg, Jr, III)	Academic Degrees	Institution	Location (city, state/province, country)	Role or Contribution, eg, chair, principal investigator	Group (if more than 1 Group listed in the byline) and/or Subgroup (eg, Steering Committee)
Stacy	Williams		RN	Nationwide Children's Hospital, The Abigail Wexner Research Institute at Nationwide Children's Hospital, Center for Perinatal Research, The Ohio State University College of Medicine	Columbus, OH	Study Site Contributor	NIH Eunice Kennedy Shriver National Institute of Child Health and Human Development Neonatal Research Network
Taise	Staton		RN	Nationwide Children's Hospital, The Abigail Wexner Research Institute at Nationwide Children's Hospital, Center for Perinatal Research, The Ohio State University College of Medicine	Columbus, OH	Study Site Contributor	NIH Eunice Kennedy Shriver National Institute of Child Health and Human Development Neonatal Research Network
Jacqueline N.	Palmer		PA-C	Nationwide Children's Hospital, The Abigail Wexner Research Institute at Nationwide Children's Hospital, Center for Perinatal Research, The Ohio State University College of Medicine	Columbus, OH	Study Site Contributor	NIH Eunice Kennedy Shriver National Institute of Child Health and Human Development Neonatal Research Network

*Indicates required information. Only first name, last name, and suffix will appear in PubMed.

*First Name and Middle Initial(s)	*Last Name	*Suffix (eg, Jr, III)	Academic Degrees	Institution	Location (city, state/province, country)	Role or Contribution, eg, chair, principal investigator	Group (if more than 1 Group listed in the byline) and/or Subgroup (eg, Steering Committee)
Jana L.	Erner		MD	Nationwide Children's Hospital, The Abigail Wexner Research Institute at Nationwide Children's Hospital, Center for Perinatal Research, The Ohio State University College of Medicine	Columbus, OH	Study Site Contributor	NIH Eunice Kennedy Shriver National Institute of Child Health and Human Development Neonatal Research Network
Jason B.	Kovalcik		MD	Nationwide Children's Hospital, The Abigail Wexner Research Institute at Nationwide Children's Hospital, Center for Perinatal Research, The Ohio State University College of Medicine	Columbus, OH	Study Site Contributor	NIH Eunice Kennedy Shriver National Institute of Child Health and Human Development Neonatal Research Network
Erin L.	Keels		DNP, APRN-CNP, NNP-BC	Nationwide Children's Hospital, The Abigail Wexner Research Institute at Nationwide Children's Hospital, Center for Perinatal Research, The Ohio State University College of Medicine	Columbus, OH	Study Site Contributor	NIH Eunice Kennedy Shriver National Institute of Child Health and Human Development Neonatal Research Network

*Indicates required information. Only first name, last name, and suffix will appear in PubMed.

***First Name and Middle Initial(s)**	***Last Name**	***Suffix (eg, Jr, III)**	Academic Degrees	Institution	Location (city, state/province, country)	Role or Contribution, eg, chair, principal investigator	Group (if more than 1 Group listed in the byline) and/or Subgroup (eg, Steering Committee)
Teri	McCarty		PharmD	Nationwide Children's Hospital, The Abigail Wexner Research Institute at Nationwide Children's Hospital, Center for Perinatal Research, The Ohio State University College of Medicine	Columbus, OH	Study Site Contributor	NIH Eunice Kennedy Shriver National Institute of Child Health and Human Development Neonatal Research Network
Katherine	Harris		CCRC, MA, MPH	Norton Children's Hospital affiliated with the University of Louisville School of Medicine	Louisville, KY	Study Site Contributor	NIH Environmental influences on Child Health Outcomes (ECHO) Program Institutional Development Award States Pediatric Clinical Trials Network
Meagan	DeSpain		CCLS, MS	Norton Children's Hospital affiliated with the University of Louisville School of Medicine	Louisville, KY	Study Site Contributor	NIH Environmental influences on Child Health Outcomes (ECHO) Program Institutional Development Award States Pediatric Clinical Trials Network
Jackie	Boyd		RN, BSN, CHPE, CCRC	Norton Children's Hospital affiliated with the University of Louisville School of Medicine	Louisville, KY	Study Site Contributor	NIH Environmental influences on Child Health Outcomes (ECHO) Program Institutional Development Award States Pediatric Clinical Trials Network

*Indicates required information. Only first name, last name, and suffix will appear in PubMed.

*First Name and Middle Initial(s)	*Last Name	*Suffix (eg, Jr, III)	Academic Degrees	Institution	Location (city, state/province, country)	Role or Contribution, eg, chair, principal investigator	Group (if more than 1 Group listed in the byline) and/or Subgroup (eg, Steering Committee)
Sara	Watson		MD, MS	Norton Children's Hospital affiliated with the University of Louisville School of Medicine	Louisville, KY	Study Site Contributor	NIH Environmental influences on Child Health Outcomes (ECHO) Program Institutional Development Award States Pediatric Clinical Trials Network
Jan	Sullivan		MD	Norton Children's Hospital affiliated with the University of Louisville School of Medicine	Louisville, KY	Study Site Contributor	NIH Environmental influences on Child Health Outcomes (ECHO) Program Institutional Development Award States Pediatric Clinical Trials Network
Timothy	Walsh		MPH	Oklahoma University Health Sciences Center	Oklahoma City, OK	Study Site Contributor	NIH Environmental influences on Child Health Outcomes (ECHO) Program Institutional Development Award States Pediatric Clinical Trials Network
Brianna	Anderson			Oklahoma University Health Sciences Center	Oklahoma City, OK	Study Site Contributor	NIH Environmental influences on Child Health Outcomes (ECHO) Program Institutional Development Award States Pediatric Clinical Trials Network
Devon	Hahn		MD	Oklahoma University Health Sciences Center	Oklahoma City, OK	Study Site Investigator	NIH Environmental influences on Child Health Outcomes (ECHO) Program Institutional Development Award States Pediatric Clinical Trials Network

Supplemental Online Content: Nonauthor Collaborators

*Indicates required information. Only first name, last name, and suffix will appear in PubMed.

***First Name and Middle Initial(s)**	***Last Name**	***Suffix (eg, Jr, III)**	Academic Degrees	Institution	Location (city, state/province, country)	Role or Contribution, eg, chair, principal investigator	Group (if more than 1 Group listed in the byline) and/or Subgroup (eg, Steering Committee)
Christi	Madden		MPA	Oklahoma University Health Sciences Center	Oklahoma City, OK	Study Site Contributor	NIH Environmental influences on Child Health Outcomes (ECHO) Program Institutional Development Award States Pediatric Clinical Trials Network
Anne	Nascimbeni		RN	Oklahoma University Health Sciences Center	Oklahoma City, OK	Study Site Contributor	NIH Environmental influences on Child Health Outcomes (ECHO) Program Institutional Development Award States Pediatric Clinical Trials Network
Shannon	Wilson		RN	Oklahoma University Health Sciences Center	Oklahoma City, OK	Study Site Contributor	NIH Environmental influences on Child Health Outcomes (ECHO) Program Institutional Development Award States Pediatric Clinical Trials Network
Theresa	Gibson		RN	Oklahoma University Health Sciences Center	Oklahoma City, OK	Study Site Contributor	NIH Environmental influences on Child Health Outcomes (ECHO) Program Institutional Development Award States Pediatric Clinical Trials Network
John	Karlin		RN	Oklahoma University Health Sciences Center	Oklahoma City, OK	Study Site Contributor	NIH Environmental influences on Child Health Outcomes (ECHO) Program Institutional Development Award States Pediatric Clinical Trials Network

*Indicates required information. Only first name, last name, and suffix will appear in PubMed.

***First Name and Middle Initial(s)**	***Last Name**	***Suffix (eg, Jr, III)**	Academic Degrees	Institution	Location (city, state/province, country)	Role or Contribution, eg, chair, principal investigator	Group (if more than 1 Group listed in the byline) and/or Subgroup (eg, Steering Committee)
Carla M.	Bann		PhD	RTI International	Research Triangle Park, NC	Data Coordinating Center Member	NIH Eunice Kennedy Shriver National Institute of Child Health and Human Development Neonatal Research Network
Jeanette	O’Donnell Auman		BS	RTI International	Research Triangle Park, NC	Data Coordinating Center Member	NIH Eunice Kennedy Shriver National Institute of Child Health and Human Development Neonatal Research Network
Eugene	Turner		BS	RTI International	Research Triangle Park, NC	Data Coordinating Center Member	NIH Eunice Kennedy Shriver National Institute of Child Health and Human Development Neonatal Research Network
Emily	Smith		MPH	RTI International	Research Triangle Park, NC	Data Coordinating Center Member	NIH Eunice Kennedy Shriver National Institute of Child Health and Human Development Neonatal Research Network
Lauren	Bradley		MPH	RTI International	Research Triangle Park, NC	Data Coordinating Center Member	NIH Eunice Kennedy Shriver National Institute of Child Health and Human Development Neonatal Research Network
Lillian	Trochinski		RN, BSN	RTI International	Research Triangle Park, NC	Data Coordinating Center Member	NIH Eunice Kennedy Shriver National Institute of Child Health and Human Development Neonatal Research Network

*Indicates required information. Only first name, last name, and suffix will appear in PubMed.

***First Name and Middle Initial(s)**	***Last Name**	***Suffix (eg, Jr, III)**	Academic Degrees	Institution	Location (city, state/province, country)	Role or Contribution, eg, chair, principal investigator	Group (if more than 1 Group listed in the byline) and/or Subgroup (eg, Steering Committee)
Jenna	Gabrio		MPH	RTI International	Research Triangle Park, NC	Data Coordinating Center Member	NIH Eunice Kennedy Shriver National Institute of Child Health and Human Development Neonatal Research Network
David	Leblond		BS	RTI International	Research Triangle Park, NC	Data Coordinating Center Member	NIH Eunice Kennedy Shriver National Institute of Child Health and Human Development Neonatal Research Network
James	Pickett		BS	RTI International	Research Triangle Park, NC	Data Coordinating Center Member	NIH Eunice Kennedy Shriver National Institute of Child Health and Human Development Neonatal Research Network
Jamie E.	Newman		PhD, MPH	RTI International	Research Triangle Park, NC	Data Coordinating Center Member	NIH Eunice Kennedy Shriver National Institute of Child Health and Human Development Neonatal Research Network
Kristin M.	Zaterka-Baxter		RN, BSN, CCRP	RTI International	Research Triangle Park, NC	Data Coordinating Center Member	NIH Eunice Kennedy Shriver National Institute of Child Health and Human Development Neonatal Research Network

*Indicates required information. Only first name, last name, and suffix will appear in PubMed.

*First Name and Middle Initial(s)	*Last Name	*Suffix (eg, Jr, III)	Academic Degrees	Institution	Location (city, state/province, country)	Role or Contribution, eg, chair, principal investigator	Group (if more than 1 Group listed in the byline) and/or Subgroup (eg, Steering Committee)
Michelle L.	Baack		MD	Sanford Health	Sioux Falls, SD	Study Site Contributor	NIH Environmental influences on Child Health Outcomes (ECHO) Program Institutional Development Award States Pediatric Clinical Trials Network
Laurie A.	Hogden		MD	Sanford Health	Sioux Falls, SD	Study Site Contributor	NIH Environmental influences on Child Health Outcomes (ECHO) Program Institutional Development Award States Pediatric Clinical Trials Network
Chelsey	Elenkiwich		NNP	Sanford Health	Sioux Falls, SD	Study Site Contributor	NIH Environmental influences on Child Health Outcomes (ECHO) Program Institutional Development Award States Pediatric Clinical Trials Network
Megan M.	Henning		RN	Sanford Health	Sioux Falls, SD	Study Site Contributor	NIH Environmental influences on Child Health Outcomes (ECHO) Program Institutional Development Award States Pediatric Clinical Trials Network
Sarah	Van Muyden		RN, BSN	Sanford Health	Sioux Falls, SD	Study Site Contributor	NIH Environmental influences on Child Health Outcomes (ECHO) Program Institutional Development Award States Pediatric Clinical Trials Network

Supplemental Online Content: Nonauthor Collaborators

*Indicates required information. Only first name, last name, and suffix will appear in PubMed.

*First Name and Middle Initial(s)	*Last Name	*Suffix (eg, Jr, III)	Academic Degrees	Institution	Location (city, state/province, country)	Role or Contribution, eg, chair, principal investigator	Group (if more than 1 Group listed in the byline) and/or Subgroup (eg, Steering Committee)
Anna-Lisa	Martino		MD	Sanford Health	Sioux Falls, SD	Study Site Contributor	NIH Environmental influences on Child Health Outcomes (ECHO) Program Institutional Development Award States Pediatric Clinical Trials Network
Allison	Lutz		RN	Sanford Health	Sioux Falls, SD	Study Site Contributor	NIH Environmental influences on Child Health Outcomes (ECHO) Program Institutional Development Award States Pediatric Clinical Trials Network
Jeralyn	Nelson		RN	Sanford Health	Sioux Falls, SD	Study Site Contributor	NIH Environmental influences on Child Health Outcomes (ECHO) Program Institutional Development Award States Pediatric Clinical Trials Network
Bette	Schumacher		CNS	Sanford Health	Sioux Falls, SD	Study Site Contributor	NIH Environmental influences on Child Health Outcomes (ECHO) Program Institutional Development Award States Pediatric Clinical Trials Network
Aimee	Brodkorb		RNC, BSN	Sanford Health	Sioux Falls, SD	Study Site Contributor	NIH Environmental influences on Child Health Outcomes (ECHO) Program Institutional Development Award States Pediatric Clinical Trials Network

Supplemental Online Content: Nonauthor Collaborators

*Indicates required information. Only first name, last name, and suffix will appear in PubMed.

***First Name and Middle Initial(s)**	***Last Name**	***Suffix (eg, Jr, III)**	Academic Degrees	Institution	Location (city, state/province, country)	Role or Contribution, eg, chair, principal investigator	Group (if more than 1 Group listed in the byline) and/or Subgroup (eg, Steering Committee)
Deborah	Pritchett		PharmD	Sanford Health	Sioux Falls, SD	Study Site Contributor	NIH Environmental influences on Child Health Outcomes (ECHO) Program Institutional Development Award States Pediatric Clinical Trials Network
Kristen	Ford		BSN, MSN	Spartanburg Regional Medical Center	Spartanburg, SC	Study Site Contributor	NIH Environmental influences on Child Health Outcomes (ECHO) Program Institutional Development Award States Pediatric Clinical Trials Network
Darla	Howard		RN	Spartanburg Regional Medical Center	Spartanburg, SC	Study Site Contributor	NIH Environmental influences on Child Health Outcomes (ECHO) Program Institutional Development Award States Pediatric Clinical Trials Network
Ryan	Orland		MD	Spartanburg Regional Medical Center	Spartanburg, SC	Study Site Contributor	NIH Environmental influences on Child Health Outcomes (ECHO) Program Institutional Development Award States Pediatric Clinical Trials Network
Monique	Diles		RN, BSN	Tulane Lakeside Hospital, Tulane University School of Medicine	Metaire, LA	Study Site Contributor	NIH Environmental influences on Child Health Outcomes (ECHO) Program Institutional Development Award States Pediatric Clinical Trials Network

Supplemental Online Content: Nonauthor Collaborators

*Indicates required information. Only first name, last name, and suffix will appear in PubMed.

***First Name and Middle Initial(s)**	***Last Name**	***Suffix (eg, Jr, III)**	Academic Degrees	Institution	Location (city, state/province, country)	Role or Contribution, eg, chair, principal investigator	Group (if more than 1 Group listed in the byline) and/or Subgroup (eg, Steering Committee)
Kay L.	Shuttleworth		PhD	University of Arkansas	Little Rock, AK	Data Coordinating Center Member	NIH Environmental influences on Child Health Outcomes (ECHO) Program Institutional Development Award States Pediatric Clinical Trials Network
DeAnn E.	Hubberd		CCRP, MA	University of Arkansas	Little Rock, AK	Data Coordinating Center Member	Child Health Outcomes (ECHO) Program Institutional Development Award States Pediatric Clinical Trials Network
Kathy D.	Edwards		RN, CCRP, CCRA, BSN	University of Arkansas	Little Rock, AK	Data Coordinating Center Member	NIH Environmental influences on Child Health Outcomes (ECHO) Program Institutional Development Award States Pediatric Clinical Trials Network
Sherry L.	Lloyd		BA	University of Arkansas	Little Rock, AK	Data Coordinating Center Member	Child Health Outcomes (ECHO) Program Institutional Development Award States Pediatric Clinical Trials Network
Catrice	Banks-Johnson		BBA, MPA	University of Arkansas	Little Rock, AK	Data Coordinating Center Member	Child Health Outcomes (ECHO) Program Institutional Development Award States Pediatric Clinical Trials Network
Patrina	Robinson		BA	University of Arkansas	Little Rock, AK	Data Coordinating Center Member	Child Health Outcomes (ECHO) Program Institutional Development Award States Pediatric Clinical Trials Network

*Indicates required information. Only first name, last name, and suffix will appear in PubMed.

***First Name and Middle Initial(s)**	***Last Name**	***Suffix (eg, Jr, III)**	Academic Degrees	Institution	Location (city, state/province, country)	Role or Contribution, eg, chair, principal investigator	Group (if more than 1 Group listed in the byline) and/or Subgroup (eg, Steering Committee)
Melissa	Griffith		RHIT	University of Arkansas	Little Rock, AK	Data Coordinating Center Member	Child Health Outcomes (ECHO) Program Institutional Development Award States Pediatric Clinical Trials Network
Amie	Slaughter		NP	University of Kansas Medical Center	Kansas City, KS	Study Site Contributor	NIH Environmental influences on Child Health Outcomes (ECHO) Program Institutional Development Award States Pediatric Clinical Trials Network
Teresa	Kilkenny		NP	University of Kansas Medical Center	Kansas City, KS	Study Site Contributor	NIH Environmental influences on Child Health Outcomes (ECHO) Program Institutional Development Award States Pediatric Clinical
Kristina	Foster		RN, MS, APRN-BC, CCRP	University of Kansas Medical Center	Kansas City, KS	Study Site Contributor	NIH Environmental influences on Child Health Outcomes (ECHO) Program Institutional Development Award States Pediatric Clinical Trials Network
Morgan	Cross		RN, BSN	University of Kansas Medical Center	Kansas City, KS	Study Site Contributor	NIH Environmental influences on Child Health Outcomes (ECHO) Program Institutional Development Award States Pediatric Clinical Trials Network
Jessica	Gier		RN, BSN	University of Kansas Medical Center	Kansas City, KS	Study Site Contributor	NIH Environmental influences on Child Health Outcomes (ECHO) Program Institutional Development Award States Pediatric Clinical

*Indicates required information. Only first name, last name, and suffix will appear in PubMed.

*First Name and Middle Initial(s)	*Last Name	*Suffix (eg, Jr, III)	Academic Degrees	Institution	Location (city, state/province, country)	Role or Contribution, eg, chair, principal investigator	Group (if more than 1 Group listed in the byline) and/or Subgroup (eg, Steering Committee)
Natalie	Roberts		RN, BSN	University of Kansas Medical Center	Kansas City, KS	Study Site Contributor	NIH Environmental influences on Child Health Outcomes (ECHO) Program Institutional Development Award States Pediatric Clinical Trials Network
Jessica	Wadman		RN	University of Kansas Medical Center	Kansas City, KS	Study Site Contributor	NIH Environmental influences on Child Health Outcomes (ECHO) Program Institutional Development Award States Pediatric Clinical Trials Network
Kathy	Cathey		RN	University of Kansas Medical Center	Kansas City, KS	Study Site Contributor	NIH Environmental influences on Child Health Outcomes (ECHO) Program Institutional Development Award States Pediatric Clinical Trials Network
Jenna	Todd		RN	University of Kansas Medical Center	Kansas City, KS	Study Site Contributor	NIH Environmental influences on Child Health Outcomes (ECHO) Program Institutional Development Award States Pediatric Clinical Trials Network
Emily	Fratesi		RNC	University of Mississippi Medical Center	Jackson, MS	Study Site Contributor	Child Health Outcomes (ECHO) Program Institutional Development Award States Pediatric Clinical Trials Network

*Indicates required information. Only first name, last name, and suffix will appear in PubMed.

***First Name and Middle Initial(s)**	***Last Name**	***Suffix (eg, Jr, III)**	Academic Degrees	Institution	Location (city, state/province, country)	Role or Contribution, eg, chair, principal investigator	Group (if more than 1 Group listed in the byline) and/or Subgroup (eg, Steering Committee)
Lacy	Malloch		BS	University of Mississippi Medical Center	Jackson, MS	Study Site Contributor	NIH Environmental influences on Child Health Outcomes (ECHO) Program Institutional Development Award States Pediatric Clinical Trials Network
Radha	Alur		MD	University of Mississippi Medical Center	Jackson, MS	Study Site Contributor	NIH Environmental influences on Child Health Outcomes (ECHO) Program Institutional Development Award States Pediatric Clinical Trials Network
Joseph Marc	Majure		MD	University of Mississippi Medical Center	Jackson, MS	Study Site Contributor	Child Health Outcomes (ECHO) Program Institutional Development Award States Pediatric Clinical Trials Network
Courtney	Walker		PhD	University of Mississippi Medical Center	Jackson, MS	Study Site Contributor	NIH Environmental influences on Child Health Outcomes (ECHO) Program Institutional Development Award States Pediatric Clinical Trials Network
Catherine	Powers		MEd	University of Mississippi Medical Center	Jackson, MS	Study Site Contributor	NIH Environmental influences on Child Health Outcomes (ECHO) Program Institutional Development Award States Pediatric Clinical Trials Network

Supplemental Online Content: Nonauthor Collaborators

*Indicates required information. Only first name, last name, and suffix will appear in PubMed.

***First Name and Middle Initial(s)**	***Last Name**	***Suffix (eg, Jr, III)**	Academic Degrees	Institution	Location (city, state/province, country)	Role or Contribution, eg, chair, principal investigator	Group (if more than 1 Group listed in the byline) and/or Subgroup (eg, Steering Committee)
Robert D.	Annett		PhD	University of Mississippi Medical Center	Jackson, MS	Study Site Contributor	NIH Environmental influences on Child Health Outcomes (ECHO) Program Institutional Development Award States Pediatric Clinical Trials Network
Rachel	Wellman		RN	University of Nebraska Medical Center	Omaha, NE	Study Site Contributor	NIH Environmental influences on Child Health Outcomes (ECHO) Program Institutional Development Award States Pediatric Clinical Trials Network
Kerry	Miller		MD	University of Nebraska Medical Center	Omaha, NE	Study Site Contributor	NIH Environmental influences on Child Health Outcomes (ECHO) Program Institutional Development Award States Pediatric Clinical Trials Network
Ann	Anderson-Berry		MD, PhD	University of Nebraska Medical Center	Omaha, NE	Study Site Contributor	NIH Environmental influences on Child Health Outcomes (ECHO) Program Institutional Development Award States Pediatric Clinical Trials Network
Hengameh	Raissy		PharmD	University of New Mexico Health Sciences Center	Albuquerque, NM	Study Site Contributor	NIH Environmental influences on Child Health Outcomes (ECHO) Program Institutional Development Award States Pediatric Clinical Trials Network

Supplemental Online Content: Nonauthor Collaborators

*Indicates required information. Only first name, last name, and suffix will appear in PubMed.

*First Name and Middle Initial(s)	*Last Name	*Suffix (eg, Jr, III)	Academic Degrees	Institution	Location (city, state/province, country)	Role or Contribution, eg, chair, principal investigator	Group (if more than 1 Group listed in the byline) and/or Subgroup (eg, Steering Committee)
Alberta	Kong		MD, MPH	University of New Mexico Health Sciences Center	Albuquerque, NM	Study Site Contributor	NIH Environmental influences on Child Health Outcomes (ECHO) Program Institutional Development Award States Pediatric Clinical Trials Network
Kate	McCalmont		MD	University of New Mexico Health Sciences Center	Albuquerque, NM	Study Site Contributor	NIH Environmental influences on Child Health Outcomes (ECHO) Program Institutional Development Award States Pediatric Clinical Trials Network
Sandra	Sundquist Beauman		RNC, MSN	University of New Mexico Health Sciences Center	Albuquerque, NM	Study Site Contributor	NIH Environmental influences on Child Health Outcomes (ECHO) Program Institutional Development Award States Pediatric Clinical Trials Network
Sarah	Sanders		RN, BSN, MSESS	University of New Mexico Health Sciences Center	Albuquerque, NM	Study Site Contributor	NIH Environmental influences on Child Health Outcomes (ECHO) Program Institutional Development Award States Pediatric Clinical Trials Network
Eric C.	Eichenwald		MD	Children's Hospital of Philadelphia	Philadelphia, PA	Study Site Contributor	NIH Eunice Kennedy Shriver National Institute of Child Health and Human Development Neonatal Research Network

Supplemental Online Content: Nonauthor Collaborators

*Indicates required information. Only first name, last name, and suffix will appear in PubMed.

***First Name and Middle Initial(s)**	***Last Name**	***Suffix (eg, Jr, III)**	Academic Degrees	Institution	Location (city, state/province, country)	Role or Contribution, eg, chair, principal investigator	Group (if more than 1 Group listed in the byline) and/or Subgroup (eg, Steering Committee)
Toni	Mancini		RN, BSN, CCRC	Children's Hospital of Philadelphia	Philadelphia, PA	Study Site Contributor	NIH Eunice Kennedy Shriver National Institute of Child Health and Human Development Neonatal Research Network
Kristina	Ziolkowski			Children's Hospital of Philadelphia	Philadelphia, PA	Study Site Contributor	NIH Eunice Kennedy Shriver National Institute of Child Health and Human Development Neonatal Research Network
Megan	Dhawan		CRNP, BSN, MSN	Children's Hospital of Philadelphia	Philadelphia, PA	Study Site Contributor	NIH Eunice Kennedy Shriver National Institute of Child Health and Human Development Neonatal Research Network
Mary Kay	Gambacorta		RN	Children's Hospital of Philadelphia	Philadelphia, PA	Study Site Contributor	NIH Eunice Kennedy Shriver National Institute of Child Health and Human Development Neonatal Research Network
Allie	Pulsifer		CRNP-PNP, MSN	Children's Hospital of Philadelphia	Philadelphia, PA	Study Site Contributor	NIH Eunice Kennedy Shriver National Institute of Child Health and Human Development Neonatal Research Network

Supplemental Online Content: Nonauthor Collaborators

*Indicates required information. Only first name, last name, and suffix will appear in PubMed.

***First Name and Middle Initial(s)**	***Last Name**	***Suffix (eg, Jr, III)**	Academic Degrees	Institution	Location (city, state/province, country)	Role or Contribution, eg, chair, principal investigator	Group (if more than 1 Group listed in the byline) and/or Subgroup (eg, Steering Committee)
Marisa	Brant		MD	Children's Hospital of Philadelphia	Philadelphia, PA	Study Site Contributor	NIH Eunice Kennedy Shriver National Institute of Child Health and Human Development Neonatal Research Network
Aida	Schumacher		RN, CNL, MSN	Children's Hospital of Philadelphia	Philadelphia, PA	Study Site Contributor	NIH Eunice Kennedy Shriver National Institute of Child Health and Human Development Neonatal Research Network
Sydney	Gocial		RN	Children's Hospital of Philadelphia	Philadelphia, PA	Study Site Contributor	NIH Eunice Kennedy Shriver National Institute of Child Health and Human Development Neonatal Research Network
Kristen	Azuma		RN	Children's Hospital of Philadelphia	Philadelphia, PA	Study Site Contributor	NIH Eunice Kennedy Shriver National Institute of Child Health and Human Development Neonatal Research Network
Elizabeth	Quigley		RN, MSN	Children's Hospital of Philadelphia	Philadelphia, PA	Study Site Contributor	NIH Eunice Kennedy Shriver National Institute of Child Health and Human Development Neonatal Research Network

Supplemental Online Content: Nonauthor Collaborators

*Indicates required information. Only first name, last name, and suffix will appear in PubMed.

*First Name and Middle Initial(s)	*Last Name	*Suffix (eg, Jr, III)	Academic Degrees	Institution	Location (city, state/province, country)	Role or Contribution, eg, chair, principal investigator	Group (if more than 1 Group listed in the byline) and/or Subgroup (eg, Steering Committee)
Whitney	Zachritz		RN, CPNP-PC, MSN	Children's Hospital of Philadelphia	Philadelphia, PA	Study Site Contributor	NIH Eunice Kennedy Shriver National Institute of Child Health and Human Development Neonatal Research Network
Nicole	Pastore		RN	Children's Hospital of Philadelphia	Philadelphia, PA	Study Site Contributor	NIH Eunice Kennedy Shriver National Institute of Child Health and Human Development Neonatal Research Network
Jessica	Barber		CRNP, MSN	Children's Hospital of Philadelphia	Philadelphia, PA	Study Site Contributor	NIH Eunice Kennedy Shriver National Institute of Child Health and Human Development Neonatal Research Network
Abby	McDowell		CRNP	Children's Hospital of Philadelphia	Philadelphia, PA	Study Site Contributor	NIH Eunice Kennedy Shriver National Institute of Child Health and Human Development Neonatal Research Network
Alissa	Ferri		RN, BSN	Children's Hospital of Philadelphia	Philadelphia, PA	Study Site Contributor	NIH Eunice Kennedy Shriver National Institute of Child Health and Human Development Neonatal Research Network

Supplemental Online Content: Nonauthor Collaborators

*Indicates required information. Only first name, last name, and suffix will appear in PubMed.

***First Name and Middle Initial(s)**	***Last Name**	***Suffix (eg, Jr, III)**	Academic Degrees	Institution	Location (city, state/province, country)	Role or Contribution, eg, chair, principal investigator	Group (if more than 1 Group listed in the byline) and/or Subgroup (eg, Steering Committee)
Joanna	Parga-Belinkie		MD	Children's Hospital of Philadelphia	Philadelphia, PA	Study Site Contributor	NIH Eunice Kennedy Shriver National Institute of Child Health and Human Development Neonatal Research Network
Ronnie	Guillet		MD, PhD	Women's and Children's Hospital of Buffalo	Buffalo, NY	Study Site Contributor	NIH Eunice Kennedy Shriver National Institute of Child Health and Human Development Neonatal Research Network
Rosemary L.	Jensen			Women's and Children's Hospital of Buffalo	Buffalo, NY	Study Site Contributor	NIH Eunice Kennedy Shriver National Institute of Child Health and Human Development Neonatal Research Network
Alison	Kent		MD FRACP, BMBS	Women's and Children's Hospital of Buffalo	Buffalo, NY	Study Site Contributor	NIH Eunice Kennedy Shriver National Institute of Child Health and Human Development Neonatal Research Network
Ann Marie	Scorsone		CCRC, MS	Women's and Children's Hospital of Buffalo	Buffalo, NY	Study Site Contributor	NIH Eunice Kennedy Shriver National Institute of Child Health and Human Development Neonatal Research Network

*Indicates required information. Only first name, last name, and suffix will appear in PubMed.

***First Name and Middle Initial(s)**	***Last Name**	***Suffix (eg, Jr, III)**	Academic Degrees	Institution	Location (city, state/province, country)	Role or Contribution, eg, chair, principal investigator	Group (if more than 1 Group listed in the byline) and/or Subgroup (eg, Steering Committee)
Satyan	Lakshminrusimha		MD	Women's and Children's Hospital of Buffalo	Buffalo, NY	Study Site Contributor	NIH Eunice Kennedy Shriver National Institute of Child Health and Human Development Neonatal Research Network
Michelle E.	Hartley-McAndrews		MD	Women's and Children's Hospital of Buffalo	Buffalo, NY	Study Site Contributor	NIH Eunice Kennedy Shriver National Institute of Child Health and Human Development Neonatal Research Network
Emily	Li		BA	Women's and Children's Hospital of Buffalo	Buffalo, NY	Study Site Contributor	NIH Eunice Kennedy Shriver National Institute of Child Health and Human Development Neonatal Research Network
Jennifer	Donato		BS	Women's and Children's Hospital of Buffalo	Buffalo, NY	Study Site Contributor	NIH Eunice Kennedy Shriver National Institute of Child Health and Human Development Neonatal Research Network
Kimberly G.	McKee		BS	Women's and Children's Hospital of Buffalo	Buffalo, NY	Study Site Contributor	NIH Eunice Kennedy Shriver National Institute of Child Health and Human Development Neonatal Research Network

*Indicates required information. Only first name, last name, and suffix will appear in PubMed.

***First Name and Middle Initial(s)**	***Last Name**	***Suffix (eg, Jr, III)**	Academic Degrees	Institution	Location (city, state/province, country)	Role or Contribution, eg, chair, principal investigator	Group (if more than 1 Group listed in the byline) and/or Subgroup (eg, Steering Committee)
Carl	D'Angio		MD	Women's and Children's Hospital of Buffalo	Buffalo, NY	Study Site Contributor	NIH Eunice Kennedy Shriver National Institute of Child Health and Human Development Neonatal Research Network
Rachel	Jones			Women's and Children's Hospital of Buffalo	Buffalo, NY	Study Site Contributor	NIH Eunice Kennedy Shriver National Institute of Child Health and Human Development Neonatal Research Network
Dacia	Terrano		RN	Women's and Children's Hospital of Buffalo	Buffalo, NY	Study Site Contributor	NIH Eunice Kennedy Shriver National Institute of Child Health and Human Development Neonatal Research Network
Stephanie	Solpietro		PNP	Women's and Children's Hospital of Buffalo	Buffalo, NY	Study Site Contributor	NIH Eunice Kennedy Shriver National Institute of Child Health and Human Development Neonatal Research Network
Lisa	Brei		RN	Women's and Children's Hospital of Buffalo	Buffalo, NY	Study Site Contributor	NIH Eunice Kennedy Shriver National Institute of Child Health and Human Development Neonatal Research Network

*Indicates required information. Only first name, last name, and suffix will appear in PubMed.

*First Name and Middle Initial(s)	*Last Name	*Suffix (eg, Jr, III)	Academic Degrees	Institution	Location (city, state/province, country)	Role or Contribution, eg, chair, principal investigator	Group (if more than 1 Group listed in the byline) and/or Subgroup (eg, Steering Committee)
Lisa	Krokovich		RN	Women's and Children's Hospital of Buffalo	Buffalo, NY	Study Site Contributor	NIH Eunice Kennedy Shriver National Institute of Child Health and Human Development Neonatal Research Network
Sema	Hart		MD	Women's and Children's Hospital of Buffalo	Buffalo, NY	Study Site Contributor	NIH Eunice Kennedy Shriver National Institute of Child Health and Human Development Neonatal Research Network
John	Pastore		MD	Women's and Children's Hospital of Buffalo	Buffalo, NY	Study Site Contributor	NIH Eunice Kennedy Shriver National Institute of Child Health and Human Development Neonatal Research Network
Ashley	Kern		RN	Women's and Children's Hospital of Buffalo	Buffalo, NY	Study Site Contributor	NIH Eunice Kennedy Shriver National Institute of Child Health and Human Development Neonatal Research Network
Margaret	Mars		RN	Women's and Children's Hospital of Buffalo	Buffalo, NY	Study Site Contributor	NIH Eunice Kennedy Shriver National Institute of Child Health and Human Development Neonatal Research Network

*Indicates required information. Only first name, last name, and suffix will appear in PubMed.

***First Name and Middle Initial(s)**	***Last Name**	***Suffix (eg, Jr, III)**	Academic Degrees	Institution	Location (city, state/province, country)	Role or Contribution, eg, chair, principal investigator	Group (if more than 1 Group listed in the byline) and/or Subgroup (eg, Steering Committee)
Patricia	Volker		RN, MSN	Women's and Children's Hospital of Buffalo	Buffalo, NY	Study Site Contributor	NIH Eunice Kennedy Shriver National Institute of Child Health and Human Development Neonatal Research Network
Annette	Mioneo Brady		RN, MSN	Women's and Children's Hospital of Buffalo	Buffalo, NY	Study Site Contributor	NIH Eunice Kennedy Shriver National Institute of Child Health and Human Development Neonatal Research Network
Kelsey	Voelker		BS	Women's and Children's Hospital of Buffalo	Buffalo, NY	Study Site Contributor	NIH Eunice Kennedy Shriver National Institute of Child Health and Human Development Neonatal Research Network
Emily	Horn		MS	Women's and Children's Hospital of Buffalo	Buffalo, NY	Study Site Contributor	NIH Eunice Kennedy Shriver National Institute of Child Health and Human Development Neonatal Research Network
Melissa	Moreland		MPH	Women's and Children's Hospital of Buffalo	Buffalo, NY	Study Site Contributor	NIH Eunice Kennedy Shriver National Institute of Child Health and Human Development Neonatal Research Network

*Indicates required information. Only first name, last name, and suffix will appear in PubMed.

***First Name and Middle Initial(s)**	***Last Name**	***Suffix (eg, Jr, III)**	Academic Degrees	Institution	Location (city, state/province, country)	Role or Contribution, eg, chair, principal investigator	Group (if more than 1 Group listed in the byline) and/or Subgroup (eg, Steering Committee)
Marcia	Kneusel		RNC, CCRC	University of South Florida	Tampa, FL	Study Site Contributor	NIH Environmental influences on Child Health Outcomes (ECHO) Program Institutional Development Award States Pediatric Clinical Trials Network
Maya	Balakrishnan		MD	University of South Florida	Tampa, FL	Study Site Contributor	NIH Environmental influences on Child Health Outcomes (ECHO) Program Institutional Development Award States Pediatric Clinical Trials Network
Jillian	Childres		PhD	University of South Florida	Tampa, FL	Study Site Contributor	NIH Environmental influences on Child Health Outcomes (ECHO) Program Institutional Development Award States Pediatric Clinical Trials Network
Melissa	Hanniman		RN, BSN	University of South Florida	Tampa, FL	Study Site Contributor	NIH Environmental influences on Child Health Outcomes (ECHO) Program Institutional Development Award States Pediatric Clinical Trials Network
Rebecca	White		RN	University of South Florida	Tampa, FL	Study Site Contributor	NIH Environmental influences on Child Health Outcomes (ECHO) Program Institutional Development Award States Pediatric Clinical Trials Network

Supplemental Online Content: Nonauthor Collaborators

*Indicates required information. Only first name, last name, and suffix will appear in PubMed.

***First Name and Middle Initial(s)**	***Last Name**	***Suffix (eg, Jr, III)**	Academic Degrees	Institution	Location (city, state/province, country)	Role or Contribution, eg, chair, principal investigator	Group (if more than 1 Group listed in the byline) and/or Subgroup (eg, Steering Committee)
Robin K.	Ohls		MD	University of Utah Medical Center	Salt Lake City, UT	Study Site Contributor	NIH Eunice Kennedy Shriver National Institute of Child Health and Human Development Neonatal Research Network
Mariana	Baserga		MD, MSCI	University of Utah Medical Center	Salt Lake City, UT	Study Site Contributor	NIH Eunice Kennedy Shriver National Institute of Child Health and Human Development Neonatal Research Network
Bhanu	Muniyappa		MD	University of Utah Medical Center	Salt Lake City, UT	Study Site Contributor	NIH Eunice Kennedy Shriver National Institute of Child Health and Human Development Neonatal Research Network
Julie	Shakib		DO, MS, MPH	University of Utah Medical Center	Salt Lake City, UT	Study Site Contributor	NIH Eunice Kennedy Shriver National Institute of Child Health and Human Development Neonatal Research Network
Carrie A.	Rau		RN, CCRC, BSN	University of Utah Medical Center	Salt Lake City, UT	Study Site Contributor	NIH Eunice Kennedy Shriver National Institute of Child Health and Human Development Neonatal Research Network
Shawna	Baker		RN	University of Utah Medical Center	Salt Lake City, UT	Study Site Contributor	NIH Eunice Kennedy Shriver National Institute of Child Health and Human Development Neonatal Research Network

*Indicates required information. Only first name, last name, and suffix will appear in PubMed.

***First Name and Middle Initial(s)**	***Last Name**	***Suffix (eg, Jr, III)**	Academic Degrees	Institution	Location (city, state/province, country)	Role or Contribution, eg, chair, principal investigator	Group (if more than 1 Group listed in the byline) and/or Subgroup (eg, Steering Committee)
Kathie	Coleman		RN	University of Utah Medical Center	Salt Lake City, UT	Study Site Contributor	NIH Eunice Kennedy Shriver National Institute of Child Health and Human Development Neonatal Research Network
Kandace M.	McGrath		BS	University of Utah Medical Center	Salt Lake City, UT	Study Site Contributor	NIH Eunice Kennedy Shriver National Institute of Child Health and Human Development Neonatal Research Network
Brandy	Petersen		RN	University of Utah Medical Center	Salt Lake City, UT	Study Site Contributor	NIH Eunice Kennedy Shriver National Institute of Child Health and Human Development Neonatal Research Network
Jael	Waldvogel		RN	University of Utah Medical Center	Salt Lake City, UT	Study Site Contributor	NIH Eunice Kennedy Shriver National Institute of Child Health and Human Development Neonatal Research Network
Monika	Bracken		RN	University of Utah Medical Center	Salt Lake City, UT	Study Site Contributor	NIH Eunice Kennedy Shriver National Institute of Child Health and Human Development Neonatal Research Network
Meredith	Ferrin		RN	University of Utah Medical Center	Salt Lake City, UT	Study Site Contributor	NIH Eunice Kennedy Shriver National Institute of Child Health and Human Development Neonatal Research Network

*Indicates required information. Only first name, last name, and suffix will appear in PubMed.

***First Name and Middle Initial(s)**	***Last Name**	***Suffix (eg, Jr, III)**	Academic Degrees	Institution	Location (city, state/province, country)	Role or Contribution, eg, chair, principal investigator	Group (if more than 1 Group listed in the byline) and/or Subgroup (eg, Steering Committee)
Holly	DeSantis		RN	University of Utah Medical Center	Salt Lake City, UT	Study Site Contributor	NIH Eunice Kennedy Shriver National Institute of Child Health and Human Development Neonatal Research Network
Traci	Perkins		RN	University of Utah Medical Center	Salt Lake City, UT	Study Site Contributor	NIH Eunice Kennedy Shriver National Institute of Child Health and Human Development Neonatal Research Network
Manndi C.	Loertscher		BS CCRP	University of Utah Medical Center	Salt Lake City, UT	Study Site Contributor	NIH Eunice Kennedy Shriver National Institute of Child Health and Human Development Neonatal Research Network
Karen	McAlmon		MD, FAAP	Winchester Hospital	Winchester, MA	Study Site Contributor	NIH Environmental influences on Child Health Outcomes (ECHO) Program Institutional Development Award States Pediatric Clinical Trials Network
Justin	Goldstein		MD, FAAP	Winchester Hospital	Winchester, MA	Study Site Contributor	NIH Environmental influences on Child Health Outcomes (ECHO) Program Institutional Development Award States Pediatric Clinical Trials Network

Supplemental Online Content: Nonauthor Collaborators

*Indicates required information. Only first name, last name, and suffix will appear in PubMed.

***First Name and Middle Initial(s)**	***Last Name**	***Suffix (eg, Jr, III)**	Academic Degrees	Institution	Location (city, state/province, country)	Role or Contribution, eg, chair, principal investigator	Group (if more than 1 Group listed in the byline) and/or Subgroup (eg, Steering Committee)
Asimenia	Angelidou		MD, FAAP	Winchester Hospital	Winchester, MA	Study Site Contributor	NIH Environmental influences on Child Health Outcomes (ECHO) Program Institutional Development Award States Pediatric Clinical Trials Network
Nancy	Donahue			Winchester Hospital	Winchester, MA	Study Site Contributor	NIH Environmental influences on Child Health Outcomes (ECHO) Program Institutional Development Award States Pediatric Clinical Trials Network
