# Supplementary material for: Influence of Eat, Sleep, and Console on Infants Pharmacologically Treated for Opioid Withdrawal: A Post Hoc Subgroup Analysis of the ESC-NOW Randomized Clinical Trial
Source: JAMA Pediatr. 2024 Apr 15;178(6):525–32. doi: 10.1001/jamapediatrics.2024.0544 (PMC11019446; doi:10.1001/jamapediatrics.2024.0544)
Supplement: Supplement 4. — Data sharing statement [file jamapediatr-e240544-s004.pdf]

# Data Sharing Statement

Devlin. Influence of Eat, Sleep, and Console on Infants Pharmacologically Treated for Opioid Withdrawal. *JAMA Pediatr*. Published April 15, 2024. doi:10.1001/jamapediatrics.2024.0544

**Data**
**Data available:** Yes
**Data types:** Deidentified participant data, Other (please specify)
**Additional Information:** Study protocol, data collection instruments, code-book, and de-identified method
**How to access data:** The deidentified dataset including outcomes through 3-months of age for the ESC- NOW trial on which this article is based has been uploaded into the Eunice Kennedy Shriver National Institute of Child Health and Human Development (NICHD) Data and Specimen Hub (DASH) repository and is publicly accessible from https://dash.nichd.nih.gov. Any variables not included in this dataset can be obtained by contacting the corresponding author. Complete URL: https://dash.nichd.nih.gov/study/424634
**When available:** beginning date: 08-10-2023

**Supporting Documents**
**Document types:** Statistical/analytic code
**How to access documents:** The Statistical/analytic code is available at the Data and Specimen Hub (DASH)repository and is publicly accessible from https://dash.nichd.nih.gov as noted above.
**When available:** beginning date: 08-10-2023

**Additional Information**
**Who can access the data:** There are no restrictions on the data
**Types of analyses:** There are no restrictions on the data
**Mechanisms of data availability:** Any variable not included in this dataset can be obtained by contacting the corresponding author
